# Tree ring segmentation performance in highly disturbed trees using deep learning

**DOI:** 10.1371/journal.pone.0321841

**Published:** 2026-06-18

**Authors:** Joe David Zambrano-Suárez, Jorge Pérez-Martín, Alberto Muñoz-Torrero Manchado, Juan Antonio Ballesteros Cánovas

**Affiliations:** 1 Department of Geology, National Natural Science Museum, Spanish Research Council, Madrid, Spain; 2 Research Institute of Water and Environmental Engineering, Universitat Politècnica de València, Valencia, Spain; 3 Department of Artificial Intelligence, National University of Distance Education, Madrid, Spain; King Saud University, SAUDI ARABIA

## Abstract

Dendrogeomorphology provides valuable insights into the dating of geomorphic events but requires complex analyses of tree-ring records from highly disturbed trees. While deep learning algorithms have been successfully applied to detect boundaries in normally developed growth rings, their performance under severely disturbed growth conditions remains largely unexplored. This study evaluates whether deep learning can effectively segment tree rings exhibiting abnormal growth patterns commonly observed in dendrogeomorphological contexts. Increment cores were collected from a debris-flow-affected area. High-resolution images were subsequently acquired and manually annotated to identify tree rings boundaries and growth disturbances. A series of experiments was conducted using different neural network architectures, image resolutions, and filtering techniques to examine the relationship between convolutional neural network (CNN)–based models and the level of cellular detail represented in the images. Our results indicate that segmentation performance declines in growth disturbances characterised by pronounced changes in colour and texture relative to normal growth patterns. Nevertheless, the proposed framework successfully identified sets of narrow ring boundaries spaced more than 200 μm apart when colour remained consistent, correctly segmenting most rings associated with the most severe growth suppressions in our dataset. Notably, models relying primarily on simple features such as colour variation performed comparably to those incorporating finer cellular details. We also found that, within a patch-based processing framework, performance decreased when growth direction was not specified in advance. Overall, this study provides a systematic evaluation of CNN-based methods under highly disturbed growth conditions, highlighting both their potential and current limitations in dendrogeomorphological applications.

## 1 Introduction

Dendrochronology is an absolute dating method that uses the structure of tree rings to determine the age of trees and analyse past climate conditions [1], making it a valuable tool in scientific fields such as climatology, ecology, archaeology, hydrology, and geomorphology, among others [2–5]. In particular, the use of tree rings to determine the spatial and temporal patterns of geomorphic processes is known as dendrogeomorphology [6]. Extreme events such as floods, debris flows, landslides, or snow avalanches frequently affect existing vegetation, disrupting the normal growth patterns of trees and shrubs [4,5,7,8]. As a result, affected trees often exhibit growth disturbances characterised by unusual ring patterns, such as changes in growth rates, variations in tissue coloration, or differences in texture [9]. Dating and identifying these growth disturbances is challenging and heavily reliant on expert analysis. In *Pinus sylvestris* L., the main geomorphological signals investigated include injuries, callus tissue, reaction wood, growth suppression, and growth release [10]. Growth suppression and growth release are characterised by abrupt changes in tree-ring growth patterns that affect the width of consecutive rings. Reaction wood is visually identified by rings that are considerably wider and slightly darker than the surrounding tissue, as well as by a higher proportion of latewood [9]. Injuries and callus tissue are closely linked [10,11]. When the cambium is disrupted by a geomorphological process, cambial activity ceases locally, and the wound margins initiate the formation of callus tissue to close the injury [11]. Callus tissue is characterised by alterations in the spatial arrangement of tree rings, including ring deformation and pronounced cellular disorganisation [9]. Injuries therefore exhibit both the callus tissue produced during wound closure and the original area where the cambial break occurred.

Classical tree-ring dating methods involve the use of binocular microscopes attached to a measuring table operated by an expert [12]. This optical approach is highly accurate but, even with mechanically assisted techniques [13,14], it requires considerable time, and discrepancies between experts’ interpretations may arise [15]. Recently, the use of digital imaging and microelectronic technologies has become increasingly popular due to their efficiency and favourable cost–benefit ratio [16–20]. These digital approaches may incorporate semi-automatic algorithms to detect ring boundaries based on changes in pixel intensity [17,18,20], but they still require expert supervision to correct measurement errors and misinterpretations. Deep learning (DL) and convolutional neural networks (CNNs) have been successfully applied across many fields to identify visual patterns and generate predictions [21–23]. CNNs extract complex contextual information from pixels and are capable of learning abstract representations [24]. For a comprehensive explanation of the foundational principles of CNNs, we refer the reader to LeCun et al. [25]. In semantic segmentation, where the objective is pixel-level classification, the encoder–decoder architecture is one of the most widely adopted approaches [26,27]. This category includes well-known models such as U-Net, V-Net, DeepLab, and fully convolutional networks (FCNs) [28–31]. U-Net demonstrates remarkable capabilities in image analysis [32], providing highly accurate image segmentation and serving as the foundation for numerous state-of-the-art DL architectures specifically developed and trained for segmentation tasks [33,34].

In recent years, CNN-based systems have been employed for the segmentation of tree-growth rings in dendrochronology [12,15,16,35–41]. García-Hidalgo et al. [41] designed a system that achieves promising results in automatic segmentation; however, it was specifically developed for wood anatomical images from thin microsections, making it unsuitable for studies involving full increment cores. To date, detection systems have successfully segmented tree-ring boundaries in clearly defined and normally developed growth patterns from increment cores and cross-sections. Nevertheless, a knowledge gap remains regarding the accurate segmentation of tree-ring boundaries in trees exhibiting abnormal growth patterns or densely packed ring sets at the macroscopic level [35,37–40]. Moreover, although systems operating at different levels of cellular detail have shown robust performance, the interaction between network architecture and cellular detail—and its impact on segmentation effectiveness—remains largely unexplored. To the best of our knowledge, only two studies have analysed these interactions [35,38]. However, these studies focus exclusively on well-defined tree-ring boundaries, deliberately avoiding irregular rings, pith regions, and other sources of noise such as mechanical disturbances. Finally, it remains unclear whether systems trained on increment core images with cellular detail at the macroscopic level can operate effectively without incorporating prior knowledge of tissue growth direction.

Here, we investigate the use of deep learning systems to distinguish tree-ring growth patterns in highly disturbed trees. As a distinctive feature, we introduce a dataset composed entirely of increment cores affected by past mass-movement processes. We then conduct a series of experiments that include (i) neural network architectures of varying complexity and (ii) diverse image preprocessing techniques. Additionally, we evaluate tree-ring segmentation performance across the main categories of disturbed tissues, corresponding to the typical disturbances generated by geomorphological processes in medium- and high-mountain environments. The experiments on architecture and image preprocessing were also designed to address, as a secondary objective, open questions in the literature regarding the relationship between network architecture and cellular-level detail, as well as the necessity of prior knowledge of tissue growth direction. By analysing the resulting segmentation, both quantitatively and qualitatively, we aim to address several key research questions (RQs): **RQ1:** What is the influence of cellular-level detail in increment cores on the effectiveness of the system for segmenting tree-ring boundaries? **RQ2:** Does system performance change when images are not adjusted to ensure a consistent growth direction? **RQ3:** Does the performance of a system for segmenting tree-ring boundaries in abnormal growth patterns improve when a greater number of samples with growth disturbances are included in the dataset? Through these research questions, we seek to gain a deeper understanding of tree-ring boundary segmentation in disturbed growth patterns, while also addressing open questions in the field of tree-ring segmentation.

## 2 Materials and methods

### 2.1 Data

#### 2.1.1 Data collection.

**Fig 1:**
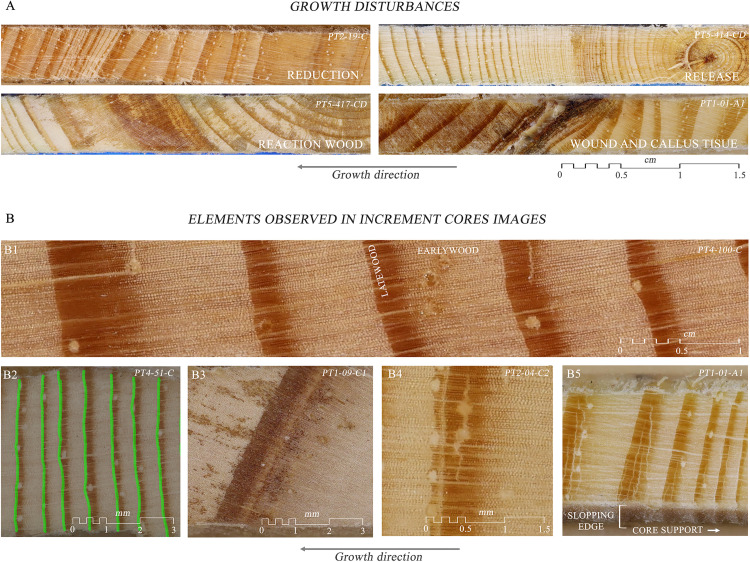
Summary of information in increment core images. **(A)** Summary of the growth disturbances presented in the images of increment cores, including injury and callus tissue, growth release, and reaction wood. **(B)** Summary of elements present in the images of increment cores: (B1) Transitions from earlywood to latewood; (B2) Typical case of a ring border; (B3) Case not labelled as a ring border due to no reduction in cell size; (B4) Case not labelled as a ring border due to a junction at a specific point between two rings; (B5) Case showing the presence of sloping edges with ring borders and regions with core support.

We used increment borers to extract wood samples from *Pinus sylvestris* individuals growing on a debris-flow cone in the Pineta Valley (Spanish Pyrenees) (S1 Appendix). Sampled trees showed external evidence of past debris-flow activity, such as decapitation, tilting, scarring, or burial [7]. Consequently, the samples included anomalous growth patterns such as abrupt suppressions and releases, reaction wood, callus tissue, and injuries [9] (Fig 1). After field collection, the samples were sanded and polished using progressively finer sandpaper (from 120 to 800 grit) to enhance tree-ring visibility. All samples were then digitised using the CaptuRING system [42]. This device was equipped with an EOS R8 digital camera fitted with a 100 mm EF macro lens, specifically designed to capture fine textures and details in close-up images. The system was complemented by an MF18 Macro Flash, which provided uniform and controlled illumination. A stitching process was subsequently applied to the sequential images using Image Composite Editor (ICE) software (Microsoft Research™). Additionally, three images from three new increment cores were reserved as part of a test dataset; however, these were photographed under lamps emitting warm yellowish light rather than using the Macro Flash. Although the samples were carefully sanded and mounted on their supports, variations in the distance between the sample surface and the camera persisted, both within and between samples. Combined with the extremely shallow depth of field inherent to macro-level imaging, this required slight adjustments to camera height to achieve optimal focus for each sample. Consequently, there is no direct and uniform correspondence between image pixels and the micrometric dimensions of the samples. Nevertheless, the high-resolution macro-imaging system enables analyses to be conducted at the micrometric scale (µm).

Regarding the level of cellular detail achieved, earlywood cells and their boundaries were generally distinguishable due to their larger lumen sizes. In contrast, latewood cells were identifiable only in certain regions, primarily during their early developmental stages, becoming indistinct at later stages (Fig 1).

#### 2.1.2 Labelling protocol.

We used a Pixel Annotation Tool [43] on a digital drawing tablet to label visible tree-ring boundaries, following a consistent direction from the outer edge of the sample towards the pith. All annotations were performed directly on the original images at their native resolution. In our dataset, ring boundaries were defined as transitions marked by a light-coloured band with larger cells preceding a latewood phase characterised by darker, smaller cells (Fig 1). False rings, being inherently indistinguishable from true rings at the macroscopic level and not clearly visible in the anatomical structure, were not annotated and were therefore excluded from this study.

Easily identifiable tree rings were annotated by members of the research team, whereas more complex cases—either difficult to delineate or requiring consistency across ambiguous regions (Fig 1)—were labelled by an expert dendrogeomorphologist [44–47]. These challenging situations included: (a) cases in which a visible colour transition was present but cell dimensions remained relatively uniform, in which case no ring boundary was annotated; (b) situations where two earlywood–latewood transitions occurred in close proximity without intervening earlywood along part of the circumference, in which only one transition was considered a true boundary; (c) partially broken increment cores, where ring boundaries were annotated if the discontinuity occurred near one side of the sample rather than close to the pith; (d) cases in which a central break extended across less than one third of the ring-boundary length, where the two separated segments were interpreted as a single ring; conversely, when the gap exceeded this proportion, the segments were treated as distinct rings and the pixels spanning the gap were not annotated; (e) image regions captured at oblique angles that occasionally displayed apparent ring boundaries or portions of the sample support, which were not annotated as true boundaries; and (f) growth disturbances producing colour shifts resembling ring transitions, which were not labelled as ring boundaries after considering the disturbance type, colour patterns, ring morphology, and spacing. The same criteria for defining tree rings were applied consistently throughout the experiment. The implications of these labelling protocols are discussed in the *Future perspectives* section.

#### 2.1.3 Description of the datasets.

We compiled two datasets with clearly distinct characteristics: a primary dataset and a focused evaluation dataset. The primary dataset comprised images from 64 increment cores of varying dimensions and tree-ring counts (S1 Table), encompassing a wide diversity of growth patterns across a total of 11,947 tree rings (Table 1). From these 64 increment cores, three groups were initially defined, and this partitioning was consistently maintained throughout the study.

**Table 1:** Summary of the characteristics of datasets and subdatasets. N Inst refers to the number of identified instances of the corresponding growth disturbance; N Tr indicates the total number of tree rings encompassed within those. ^a^Not all tree rings were annotated, as only those clearly and fully affected by the growth disturbance were labelled.

Name dataset	Full increment core	Data partition	N images	N Tree rings	Callus tissue		Reaction wood		Injuries		Growth reduction	
					N Inst	N Tr	N Inst	N Tr	N Inst	N Tr	N Inst	N Tr
Primary dataset	Yes	Training, validation, internal test	55	10,608	12	198	24	442	18	278	85	1872
		External test	6	904	1	13	1	43	1	21	4	75
		Coloured external test	3	435	2	52	0	0	0	0	6	132
Focused evaluation dataset	No	Callus tissue	10	–	10	37^a^	–	–	–	–	–	–
		Reaction wood tissue	10	–	–	–	10	57 ^a^	–	–	–	–
		Injured tissue	10	–	–	–	–	–	10	62 ^a^	–	–
		Growth suppression tissue	10	–	–	–	–	–	–	–	10	95

The focused evaluation dataset consisted of 40 images, with 10 images representing each type of growth disturbance: callus tissue (CT), reaction wood (RW), injuries (IN), and growth suppression (GS) (Table 1). These images contained only the disturbed tissue rather than full increment cores and were obtained from 36 increment cores not included in the primary dataset. Furthermore, within each disturbance category, all 10 images originated from different increment cores. All visible tree rings were annotated in both the primary dataset and the focused evaluation dataset. However, in the focused evaluation dataset, certain tree rings were intentionally excluded from the evaluation in images corresponding to specific growth-disturbance categories. In images containing callus tissue, reaction wood, or injuries, only those tree rings whose visual characteristics were clearly and substantially altered by the presence of disturbed tissue were considered for evaluation. This selective strategy was adopted because establishing a clear and consistent boundary delineating the onset and spatial extent of the morphological influence exerted by disturbance tissue was not feasible. Finally, in the 10 images reserved for evaluating growth suppression tissues, the distances between adjacent tree rings were measured to assess model performance in densely packed ring structures. These measurements were obtained using a LINTAB system (Rinntech, Heidelberg, Germany). We manually measured the distance to the nearest neighbouring ring on both sides in order to quantify ring proximity. The measurements are provided in S2 Table.

### 2.2 Dataset preprocessing

#### 2.2.1 Preprocessing methodology.

The preprocessing steps were applied for two main purposes. The first group comprised procedures associated with the patchify framework, including the use of the patchify approach itself, resizing of increment-core images, and standardisation of growth direction. The second group consisted of image signal-processing filters, such as Gaussian blur and Contrast-Limited Adaptive Histogram Equalization (CLAHE), introduced to extract information relevant to the research questions.

The following sections describe the preprocessing steps in the order in which they were applied within the data-processing workflow. For additional details, see S3 Table.

**Positioning of core-growth.** Increment-core images exhibited growth in both directions along the x-axis, which posed a challenge for the neural network in a patch-based prediction context. In a patchify setting, the main difficulty lay in distinguishing the target transition—from latewood to earlywood—from confounding patterns such as the earlywood-to-latewood transition. To minimise this ambiguity, images in most datasets were standardised by aligning the growth direction towards the left, splitting increment-core images when necessary to ensure consistent orientation. An exception was made for one dataset, in which core images intentionally retained growth in both directions. This configuration was included to address one of the research questions.

**Resizing.** Datasets in the different experiments were resampled to a specific pixel height while preserving the original aspect ratio (S1 Fig). Experiments were conducted using power-of-two dimensions within the range of image sizes employed in previous patchify-based systems [39,40]: 128 × 128, 256 × 256, 512 × 512, and 1024 × 1024 pixels. This approach satisfied the requirements of the patchify framework, which slices windows exclusively along the x-axis without introducing uncertainty associated with information loss along the y-axis. However, when datasets were resized to smaller heights, increment-core images were downsampled, resulting in a loss of detail—particularly at the cellular level—although overall colour contrast at tree-ring boundaries was preserved (S1 Fig).

**Gaussian blur filter.** Gaussian blurring was applied as the image-smoothing technique. This method reduces high-frequency content—corresponding to fine cellular detail—by convolving each image with a low-pass Gaussian kernel [45]. The kernel size was set to 15 pixels, and sigma values were automatically computed from this size using default parameters [45]. This configuration was validated through multiple inspections of the reserved image sets used for training, validation, and testing. We confirmed that it effectively degraded cellular-level detail and blurred colour boundaries between earlywood and latewood across most sample regions (S2 Fig).

**CLAHE.** Contrast-Limited Adaptive Histogram Equalization (CLAHE) was applied to enhance cellular boundaries within increment-core images [46,48]. Because this method performs histogram equalization in local regions while limiting contrast amplification, it enhances local contrast without substantially increasing noise [49]. After repeated inspections of the reserved training, validation, and test images, we determined that the default contrast-limiting threshold of 40 and a grid size of 8 pixels [45] effectively highlighted cellular edges across most tissue regions (S2 Fig).

**Fig 2:**
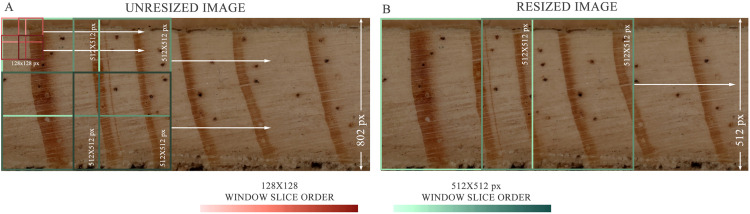
Example of two patchify processes applied to an unresized image and a resized image. Purple boxes represent windows of size 128 × 128 pixels, and green boxes represent windows of size 512 × 512 pixels. **(a)** Display using the original core image with a height of 802 pixels. **(b)** Display after resizing the core image to a height of 512 pixels while maintaining its aspect ratio.

**Patchify process.** The large dimensions of the images and the marked disproportion between their height and width (S1 Table) required the use of a patchify approach for tree-ring boundary segmentation [50,51]. Prior to each training session, increment cores assigned to the training and validation sets were divided into patches. After model calibration, predictions were generated for increment-core images in the test set using a sliding-window approach with overlapping patches. The window advanced in steps corresponding to 50% of its total size, producing a 50% overlap between consecutive patches. The final pixel probability was computed as the average prediction across overlapping patches (Fig 2).

#### 2.2.2 Derived datasets.

From the original dataset, comprising the primary dataset and the focused evaluation dataset, different combinations of preprocessing methods were applied, resulting in seven distinct dataset configurations (S3 Table). It is important to note that, throughout the study, model training and validation were conducted exclusively on the primary dataset, each time using its corresponding preprocessing configuration. For evaluation, both the primary dataset and the focused evaluation dataset were used. The former was employed to assess overall model performance on increment cores affected by growth disturbances, whereas the latter was specifically designed to evaluate performance within disturbed tissues.

### 2.3 Deep learning architectures

#### 2.3.1 U-Net architecture.

U-Net is a deep learning architecture originally developed for image segmentation in biomedical imaging [28] and subsequently extended to multiple fields due to its strong performance [50,52,53]. The architecture consists of two main components arranged in a symmetric U-shaped structure, where the input is the image to be analysed and the output is a probability map indicating, for each pixel, the likelihood of belonging to each target class.

**Fig 3:**
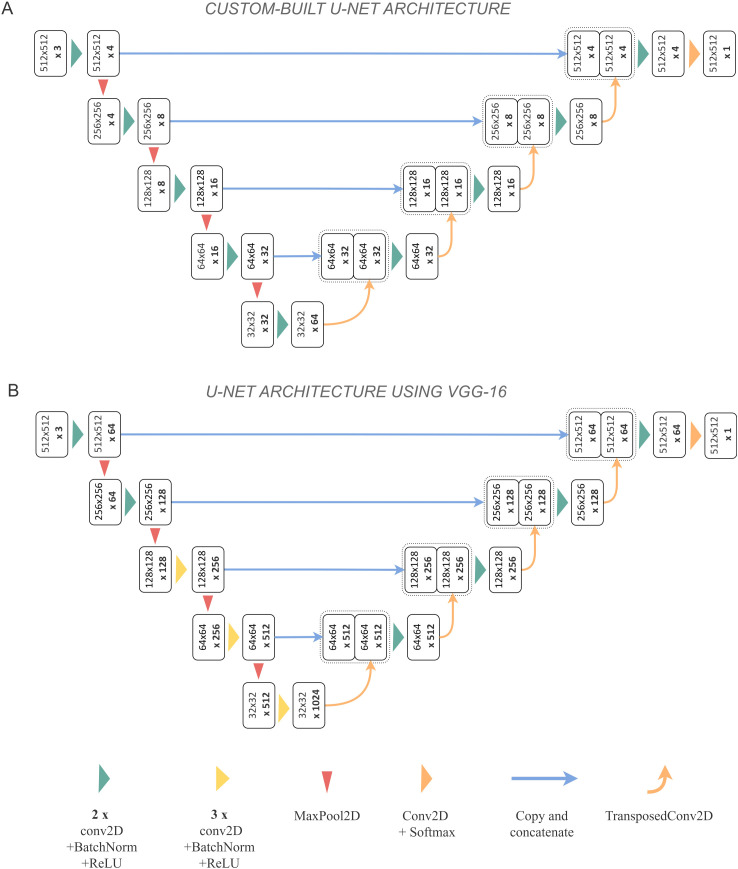
U-Net architectures used in this work. **(a)** Custom-built U-Net architecture used in this study, based on [39]. **(b)** U-Net architecture using VGG-16 as the backbone, employing transfer learning and fine-tuning.

The first component is the left-side subnetwork, or encoder, which performs downsampling operations to capture contextual information and transform the input image into multi-scale feature representations [32]. The second component is the right-side subnetwork, or decoder, which functions as the upsampling path. It reconstructs the spatial resolution by mapping the encoded representations back to pixel space through transposed convolutions, thereby facilitating precise localisation [54] (Fig 3).

The encoder is composed of sequential blocks, each formed by repeated convolution–batch normalization–Rectified Linear Unit (ReLU) activation operations. Convolutional layers extract spatial and contextual features from the input tensors, capturing patterns and generating feature maps that contribute to minimizing the loss function [55]. Batch normalization standardizes neuron activations within each batch, thereby stabilizing and accelerating training. Finally, the ReLU activation function introduces non-linearity into the network, enabling it to learn complex relationships [25]. After one or more convolution–normalization–ReLU operations, max pooling is applied to reduce the spatial resolution of the feature maps (i.e., their width and height) while retaining the most relevant information [56]. Progressively more abstract and complex feature representations are obtained along the contracting path until reaching the bottleneck, where the expansive path begins [25].

In the decoder, each processing block begins with a transposed convolution that increases the spatial resolution of the feature map. The upsampled feature map is then concatenated, via skip connections, with the corresponding feature map from the encoder [28], allowing the integration of fine-grained spatial information from the contracting path. Convolutional layers are subsequently applied to the concatenated tensor to refine and process the combined features.

Thus, owing to its symmetric U-shaped design—comprising encoder and decoder paths connected through skip connections—U-Net effectively integrates both local and global image information [57]. This property accounts for its widespread adoption in applications focused on tree-ring detection [12,35–37,39,41]. However, other studies have also reported strong performance using instance segmentation architectures [40,38], an aspect further discussed in the *Future perspectives* section.

#### 2.3.2 Proposed U-Net and variant architectures.

Several modifications of the original U-Net architecture [28] have been proposed, including Attention U-Net [33] and Attention Residual U-Net [58], which were selected in this study due to their specific advantages, as detailed in the descriptions of the custom-built architectures.

The design of these custom architectures—such as the number of filters per convolutional layer, the number of convolutional blocks at each level, and the total network depth—followed the guidelines provided by Fabijańska and Cahalan [39]. In their work, the authors progressively reduced the number of filters per convolutional layer in the original Attention U-Net architecture [33], while monitoring model performance until a decline in capacity was observed relative to the original configuration. This strategy reduced the number of parameters and computational cost, but also limited the network’s ability to capture complex features and abstract patterns. The custom-built models were initialized with random weights and trained using a learning rate (lr) of 1 × 10^–^³. Their specific architectures are outlined as follows.

**Custom-built U-Net (CU-Net).** A U-Net architecture (Fig 3) was employed. The convolutional blocks consisted of two 3 × 3 convolutions, starting with 4 filters in the first block and increasing to 64 in the deepest block, with a 2 × 2 max pooling applied after each block.

**Custom-built Attention U-Net (CAU-Net).** This architecture was originally proposed by Oktay et al. [33] and incorporates modifications in the skip connections between the feature maps of the decoder and the encoder. Instead of directly concatenating both layers, an attention mechanism is introduced to weight the incoming information from the encoder feature maps, determining which information is relevant and should pass to the decoding stage. Thus, the model learns to suppress irrelevant regions and highlight the important features useful for the segmentation task [33]. In this study, soft attention gates were applied, where the value at each point of the attention map is between 0 and 1, allowing the encoder features to be weighted smoothly.

**Custom-built Attention Residual U-Net (CARU-Net).** Attention U-Net was modified to incorporate residual convolutional blocks instead of standard convolutional blocks [58,59]. In this modification, a 1 × 1 convolution followed by normalization was applied to the input of the block, which was then added to the block output through a residual connection. The resulting feature maps were summed, and a ReLU activation function was applied to the result. In this way, the residual blocks helped maintain and improve the quality of the feature representations.

#### 2.3.3 U-Net using pre-trained models.

The custom-built U-Net architectures were optimized to work with image patches containing one or two tree rings [39], whereas the datasets used in this study often include multiple growth rings within each patch (Fig 2). Therefore, we employed more complex architectures capable of learning more intricate representations and handling data with greater variability (Table 2). To this end, transfer learning and fine-tuning techniques were applied, which consist of adapting previously trained models to address a different task.

**Table 2:** Trainable parameters in each architecture.

Model	Trainable parameters
**CU-Net**	123,763
**CAU-Net**	147,165
**CARU-Net**	153,537
**TLU-Net**	14,097,153
**FTU-Net**	21,179,649

The employed architecture incorporated the VGG-16 model [60] as the encoder subnetwork, excluding the dense layers of the original architecture, while the decoder symmetrically reconstructed the encoder subnetwork with randomly initialized weights. Therefore, VGG-16 functioned as a feature extractor, as the model had been trained on a large dataset, ImageNet [61], and had learned robust representations of low-level features such as edges, spatial patterns, rotation, lighting, and shapes [62]. The architecture started with a first convolutional block containing 64 filters and reached 1024 filters at the deepest level. It included convolutional blocks composed of three convolutional layers (Fig 3).

**U-Net with transfer learning (TLU-Net).** The encoder path had frozen weights throughout training, while the decoder weights were trainable using a learning rate of 1 × 10^–^³. This configuration implied that the features previously learned by VGG-16 were sufficient to perform the new task.

**U-Net with fine-tuning (FTU-Net).** This approach involved unfreezing part of the pre-trained model weights for training. Initially, only the decoder was trainable, with a learning rate of 1 × 10^–^³. From epoch 40 onward, the weights of the last convolutional block of the encoder were also made trainable, with a new learning rate set to 1 × 10^–^⁴. This strategy allowed for the adaptation of more complex and abstract features to the target dataset.

### 2.4 Experimental design

The experimental design was based on seven datasets generated after the preprocessing stage (S3 Table and S3 Fig). Each dataset comprised the 64 increment cores from the primary dataset and the 40 images from the focused evaluation dataset, the latter reserved exclusively for evaluation. Of the 64 increment cores, 55 were allocated to training, validation, and internal testing (inTEST) according to the defined partitioning strategies. Six representative images were reserved as the external test dataset (exTEST), and three images acquired without Macro Flash were used as the coloured external test dataset (colexTEST). The assignment of increment cores to each subset was consistent across all experiments. The seven datasets differed only in the preprocessing applied, while maintaining identical train, validation, inTEST, exTEST, and colexTEST splits. For each dataset, all test images were processed using the same preprocessing pipeline.

Two partitioning strategies were implemented. In the first, applied only in the initial experiments, three models were trained using a random selection of 40 increment cores for training, 10 for validation, and 5 for inTEST. In the second strategy, used in the remaining experiments, two rounds of 10-fold cross-validation were performed, with 40 cores assigned to training, 10 to validation, and 5 to inTEST in each run. This design reduced computational demands, as performing two full rounds of cross-validation in the first experimental group would have substantially increased resource requirements. Accordingly, evaluation with the focused evaluation dataset and Seg-Grad-CAM analysis were conducted only in the second, third, and fourth experimental groups. The limitations of this design are discussed in the *Future perspectives* section.

A threshold of 0.5 was applied to prediction outputs. Given the highly polarized prediction histograms, with few intermediate values, this cutoff was considered appropriate and stable. However, intermediate probabilities may represent either true ring boundaries or non-boundary regions. Although these opposing effects were assumed to balance each other, such ambiguities warrant further investigation. Finally, multiple architecture–dataset combinations were examined, each corresponding to training and testing a specific architecture on a given dataset. These combinations are presented below according to their experimental stage.

**First experiments.** The five architectures considered here were trained on two different datasets, in which the images were resized to a height of 512 pixels (referred to as 512pxData) in one case and 1024 pixels (referred to as 1024pxData) in the other. Since each architecture was tested on both datasets, this setup resulted in a total of ten architecture–dataset combinations. This set of experiments aimed to determine which types of architectures were most suitable and whether using the larger and more computationally demanding 1024pxData provided sufficient benefits to justify its use.

**Second experiments.** TLU-Net and FTU-Net were trained on 512pxData. This set of experiments examined whether transfer learning or fine-tuning constituted the more effective training approach.

**Third experiments.** In these experiments, datasets with image heights of 256 and 128 pixels were generated, referred to as 256pxData and 128pxData, respectively. FTU-Net was trained on 256pxData and 128pxData. The objective of these experiments was to determine whether segmentation performance was preserved as the image size, and therefore the amount of available information, is progressively reduced.

**Fourth experiments.** Three datasets were generated from 512pxData: one by applying CLAHE, referred to as 512pxCLData; another by applying a Gaussian blur filter, resulting in 512pxGData; and a third exhibiting growth in both directions, referred to as 512pxBData. FTU-Net was trained on 512pxCLData, 512pxGData, and 512pxBData. The experiments using 512pxGData and 512pxCLData investigated how the architecture behaved under lower and higher levels of available visual information, respectively. The combination with 512pxBData examined whether standardizing the growth direction of the increment cores was necessary.

### 2.5 Evaluation

#### 2.5.1 Metrics.

An evaluation procedure was designed to assess segmentation performance in both normal and abnormal growth patterns across experiments. In samples with normal growth, predicted tree-ring borders typically overlap with the ground-truth labels, whereas precise overlap is less common under disturbed growth conditions. Previous studies focusing on normal growth patterns have employed instance-level evaluation [35,37–40], grouping predicted border pixels into entities and comparing them with ground-truth instances. In contrast, Ge et al. [12] addressed abnormal growth patterns using pixel-level evaluation, enabling a more fine-grained assessment of segmentation performance.

In the present study, evaluation was conducted primarily at the pixel level and complemented by visual inspection. Pixel-level assessment was preferred because instance-level evaluation requires a prior categorization step that may obscure subtle segmentation variations, particularly in abnormal growth patterns. Furthermore, downsampling to 128pxData introduced minor artefacts into the ground-truth masks of very thin and densely packed ring bundles, potentially affecting instance-level metrics. A detailed analysis of these effects is provided in the Results and Discussion sections. Nevertheless, instance-level evaluation was performed as a complementary analysis.

For both pixel- and instance-level evaluations, a filtering step was applied prior to metric computation. Predicted regions labelled as rings that were shorter than 5% of the image height were removed to eliminate scattered predictions and prevent inflation of false positives in instance-level metrics. Consequently, rings with a relative image height below 5% could not be correctly predicted. However, only 2% of the rings in the primary dataset fell within this range. Percentile analysis (S4 Table) indicates that these rings correspond either to the first year at the core centre or to rings located in broken or fragmented core sections. No additional post-processing steps were applied.

Model performance was evaluated using precision, recall, Dice score, and F1 score. True positives (TP) denote correctly classified positive pixels or instances; false positives (FP) correspond to negative pixels or instances incorrectly identified as positive; true negatives (TN) represent correctly identified negatives; and false negatives (FN) indicate positives incorrectly classified as negative. At the instance level, predictions were considered correct when the Intersection over Union (IoU) [63] exceeded 0.5; otherwise, they were classified as false positives.

**Precision.** Measures the proportion of correctly predicted positive pixels or instances (TP) out of all pixels or instances predicted as positive by the model (TP + FP) (Eq 1).


$$\begin{array}{c} \text{Precision}=\frac{\text{TP}}{\text{TP}+\text{FP}} \end{array}$$ (1)

**Recall.** Measures the proportion of correctly predicted positive pixels or instances (TP) out of all actual positive pixels or instances (TP + FN) (Eq 2).


$$\begin{array}{c} \text{Recall}=\frac{\text{TP}}{\text{TP}+\text{FN}} \end{array}$$ (2)

**Dice similarity coefficient.** Quantifies the similarity or the overlap between two sets (Eq 3).


$$\begin{array}{c} \text{DICE}=\frac{\text{2}|\text{A }\cap \text{ B}|}{\left|\text{A}|+|\text{B}\right|}=\frac{\text{2}\text{ TP}}{\text{2}\text{ TP}+\text{FP}+\text{FN}} \end{array}$$ (3)

**F1 score**. The harmonic mean of precision and recall, balancing false positives and false negatives (Eq 4).


$$\begin{array}{c} \text{F1 score}=\frac{\text{2}\cdot \text{ Precision }\cdot \text{ Recall }}{\text{Precision}+\text{Recall}}=\frac{\text{2}\text{ TP}}{\text{2}\text{ TP}+\text{FP}+\text{FN}} \end{array}$$ (4)

where, in the case of the Dice similarity coefficient, |A| denotes the number of pixels in the predicted mask, |B| represents the number of pixels in the ground-truth label, and |A ∩ B| corresponds to the number of pixels in the overlapping region.

The images used in this study are highly imbalanced. Therefore, the Dice coefficient, which disregards the majority background class, is appropriate for evaluating performance on this dataset. The F1 score was selected to ensure comparability with previous studies, as it is commonly used in tree-ring segmentation research. Furthermore, in this binary setting, the F1 score is mathematically equivalent to the Dice coefficient and therefore also accounts for class imbalance. All evaluation metrics used in this study range from 0 to 1 and are dimensionless.

#### 2.5.2 Focused evaluation dataset.

In the focused evaluation dataset, all tree rings in each image were examined for growth suppression, whereas for other types of growth disturbances only the clearly affected tree rings were considered. To enable pixel-level and instance-level assessment in these cases, a mask was defined to delimit the area to be analysed according to specific criteria. This consisted of including the earlywood of the tree ring immediately preceding the youngest affected ring, all affected tree rings, and extending the mask to the end of the oldest affected tree ring.

Another important aspect of the focused evaluation dataset concerns the measurement of the minimal distance between locations where the neural network performs poorly. As previously noted, downsampling of the original labelling introduces artefacts in 128pxData, which can compromise the reliability of instance-level evaluation. Because this evaluation is critical to the present study, each prediction in densely packed regions—particularly with respect to minimal distance—was manually classified by the authors as correct or incorrect.

The applied criteria were as follows: a prediction had to correctly delineate at least half of the tree-ring border; it could not touch any neighbouring predicted tree-ring border; and it could not overlap a tree-ring border that remained unpredicted. Visual interpretations of the ten growth-suppression images, together with additional discussion, are provided in the following sections. For full transparency, all predictions and their corresponding classifications are included in the Supplementary Data.

Evaluating the predictions produced by the 20 models for each of the three architecture–dataset combinations would have been time-consuming. Therefore, for each combination, the mean predicted probability at each pixel across the 20 models was computed, and this averaged prediction was evaluated using a threshold of 0.5.

#### 2.5.3 Seg-Grad-CAM.

Interpretability plays a fundamental role in machine learning, as it enables a deeper understanding of a model’s internal decision-making processes [64]. Nonetheless, several techniques grounded in diverse methodological approaches have been proposed to approximate and evaluate the interpretability of deep learning models [64–71].

Here, Seg-Grad-CAM was used to assess whether the architecture–dataset combinations exploited cellular details. This technique was selected because it was specifically designed to interpret segmentation models based on CNNs and because of its relative simplicity of use [70]. Seg-Grad-CAM is based on Gradient-weighted Class Activation Mapping (Grad-CAM), a technique that derives a spatial importance map by analysing how gradients associated with a target concept propagate back to the convolutional feature maps of the layer under study [66]. Adapted to the segmentation task, Seg-Grad-CAM uses as its target concept a group of pixels by integrating the model’s class responses within a selected region of interest to form a single scalar value, whose gradients are then propagated back to the convolutional features of the studied layer.

Two configuration choices were made for the Seg-Grad-CAM analysis. First, the target concepts were defined as the pixels predicted as tree rings. Second, the technique was applied across all model layers (a detailed explanation is provided in S2 Appendix). For this analysis, one model from each architecture–dataset combination was selected, specifically those trained using the first split of the second cross-validation process. This selection was random, and the chosen models were assumed to be representative of the others. Finally, the images used in this analysis belonged to the focused evaluation dataset.

### 2.6 Implementation details

The training sessions were conducted on the HPC cluster Drago, part of the Spanish National Research Council (CSIC). Two of its nodes are equipped with 512 GB of memory, dual Intel Xeon Gold 6248R processors (24 cores each, running at 3.0 GHz), and four NVIDIA Ampere A100 GPUs (80 GB). The storage configuration includes two 240 GB SSDs and a 10 TB NVMe PCIe 4.0 volume. The operating system was Rocky Linux, and the software environment consisted of Python 3.6.8, TensorFlow 2.11, and Keras 2.11. The GPU software stack included CUDA 11.7 and cuDNN 8.3.2.

The training hyperparameters for each architecture–dataset combination were established empirically based on preliminary experiments and are detailed in S5 Table. The data augmentation strategy applied to the training patches is also reported there. For each architecture–dataset combination, these parameters were kept constant throughout the cross-validation procedure. S3 Appendix reports the number of epochs, as well as the training and prediction times required for each model.

Due to class imbalance, the Dice loss function was employed, defined as Dice loss = 1 − Dice coefficient. The batch size was adjusted according to the available GPU memory, and all models were trained using the Adam optimizer with default parameters. A dropout rate of 0.3 was applied. The maximum number of training epochs was set to 200; however, most models converged before reaching this limit (S3 Appendix). To mitigate overfitting, early stopping based on the Dice metric on the validation set was implemented with a patience of 20 epochs. Additionally, all learning rates were initially set to 1 × 10^–^³.

For FTU-Net, training began with a transfer-learning phase. After 40 epochs, the last encoder block was unfrozen, and to prevent substantial parameter updates in this layer, a learning rate decay factor of 0.1 was applied.

## 3 Results

Comparison of segmentation performance between resolution maintenance and downscaling. The metric results of the models from the first experiments are fully reported in S4 Appendix. Here, they were grouped into two categories: models trained on the 512pxData and 1024pxData datasets (Table 3). The Dice values of the models trained on 512pxData and 1024pxData were of similar magnitude, with those trained on 512pxData being slightly higher in inTEST, considerably higher in exTEST, and marginally higher in colTEST, with Dice score differences of 0.012, 0.025, and 0.007, respectively. The models trained on 512pxData required 215 ± 64.4 minutes, while those trained on 1024pxData took approximately three times longer, requiring 684.8 ± 241.9 minutes (S3 Appendix). Furthermore, in inTEST and exTEST, the models trained on 1024pxData showed less variance than those trained on 512pxData.

**Table 3:** Evaluation pixel metrics resulting from models in the first, second, third, and fourth experiments, grouped by dataset and/or architecture.

			Metrics	inTEST	exTEST	colexTEST
**1ST SET OF EXPERIMENTS**	**DATASETS**	**512pxData**	Dice	0.691 + /- 0.0604	0.706 + /- 0.0657	0.554 + /- 0.1311
			Precision	0.650 + /- 0.0679	0.713 + /- 0.0609	0.639 + /- 0.0581
			Recall	0.742 + /- 0.0776	0.707 + /- 0.0951	0.518 + /- 0.1731
		**1024pxData**	Dice	0.679 + /- 0.0675	0.681 + /- 0.1118	0.547 + /- 0.1036
			Precision	0.679 + /- 0.0594	0.738 + /- 0.0418	0.645 + /- 0.0550
			Recall	0.695 + /- 0.1204	0.654 + /- 0.1611	0.501 + /- 0.1551
	**ARCHITECTURES**	**Custom-built algorithms**	Dice	0.655 + /- 0.0609	0.650 + /- 0.0950	0.482 + /- 0.1023
			Precision	0.647 + /- 0.0728	0.702 + /- 0.0577	0.627 + /- 0.0650
			Recall	0.682 + /- 0.1165	0.629 + /- 0.1508	0.420 + /- 0.1507
		**Pre-trained algorithms**	Dice	0.730 + /- 0.0337	0.758 + /- 0.0109	0.653 + /- 0.0150
			Precision	0.691 + /- 0.0384	0.760 + /- 0.0075	0.665 + /- 0.0265
			Recall	0.773 + /- 0.0343	0.757 + /- 0.0246	0.644 + /- 0.0327
**2ND SET OF EXPERIMENTS**	**ARCHITECTURES**	**TLU-Net**	Dice	0.732 + /- 0.0254	0.767 + /- 0.0032	0.669 + /- 0.0078
			Precision	0.713 + /- 0.0329	**0.762 + /- 0.0105**	0.672 + /- 0.0207
			Recall	0.754 + /- 0.0404	0.771 + /- 0.0125	0.668 + /- 0.0244
		**FTU-Net**	Dice	0.735 + /- 0.0243	**0.772 + /- 0.0017**	0.674 + /- 0.0067
			Precision	0.709 + /- 0.0298	0.759 + /- 0.0038	0.680 + /- 0.0131
			Recall	0.765 + /- 0.0422	0.786 + /- 0.0044	0.669 + /- 0.0226
**3 RD SET OF EXPERIMENTS**	**DATASETS**	**256pxData**	Dice	0.733 + /- 0.0223	0.768 + /- 0.0029	**0.692 + /- 0.0053**
			Precision	0.663 + /- 0.0303	0.714 + /- 0.0108	0.656 + /- 0.0116
			Recall	0.820 + /- 0.0377	0.832 + /- 0.0117	0.732 + /- 0.0147
		**128pxData**	Dice	0.683 + /- 0.0306	0.718 + /- 0.0096	0.666 + /- 0.0076
			Precision	0.576 + /- 0.0477	0.614 + /- 0.0276	0.562 + /- 0.0156
			Recall	**0.846 + /- 0.0489**	**0.866 + /- 0.0284**	**0.819 + /- 0.0238**
**4TH SET EXPERIMENTS**	**DATASETS**	**512pxGData**	Dice	0.720 + /- 0.0272	0.754 + /- 0.0045	0.626 + /- 0.0210
			Precision	0.712 + /- 0.0356	0.758 + /- 0.0062	**0.712 + /- 0.0300**
			Recall	0.729 + /- 0.0388	0.750 + /- 0.0106	0.560 + /- 0.0350
		**512pxCLData**	Dice	**0.738 + /- 0.0236**	0.772 + /- 0.0023	0.687 + /- 0.0042
			Precision	**0.713 + /- 0.0300**	0.757 + /- 0.0047	0.662 + /- 0.0088
			Recall	0.766 + /- 0.0352	0.786 + /- 0.0066	0.714 + /- 0.0142
		**512pxBData**	Dice	0.659 + /- 0.1539	0.719 + /- 0.0060	0.578 + /- 0.0128
			Precision	0.652 + /- 0.1522	0.732 + /- 0.0086	0.590 + /- 0.0210
			Recall	0.667 + /- 0.1603	0.707 + /- 0.0103	0.567 + /- 0.0185

All custom-built U-Net models demonstrated lower predictive performance compared to the pre-trained models (Table 3). CU-Net, CAU-Net, and CARU-Net showed, in the majority of cases, improvements in Dice scores when trained on 512pxData compared to 1024pxData (S4 Appendix). Similarly, pre-trained models performed worse when the image resolution was maintained.

Pre-trained models demonstrated superior segmentation capability (Dice score = 0.758) compared to the custom-built models (Dice score = 0.650) in exTEST (Table 3). Our analysis suggests that models from the pre-trained group required less training time (352.638 ± 187.354 minutes) compared to the custom-built group (515.151 ± 338.977 minutes) (S3 Appendix). Pre-trained models also showed better performance in both precision and recall, with improvements of 0.058 and 0.128, respectively, indicating lower commission and omission errors in exTEST. In inTEST and colexTEST, the pre-trained models also exhibited notably better Dice scores, with differences of 0.075 and 0.171, respectively.

### 3.1 Comparison performance between fine-tuning and transfer learning techniques

The metrics obtained from exTEST, colexTEST, and inTEST for the results of the second set of experiments are provided in S4 Appendix and are summarized in Table 3. The paired t-test (p-value < 0.001) supported the hypothesis that TLU-Net (Dice score = 0.767) and FTU-Net (Dice score = 0.772) performed differently in exTEST according to the Dice metric (Table 4). Similarly, the paired t-test (p-value = 0.006) confirmed a statistically significant difference in colexTEST, where FTU-Net (Dice score = 0.674) achieved a positive difference of 0.005 with respect to TLU-Net. Likewise, the paired t-test (p-value = 0.0081) indicated a significant difference in the Dice metric in inTEST, with FTU-Net achieving the higher score.

**Table 4:** Results of the pairwise Dice score comparison of FTU-Net models on test datasets across different resized datasets in the third and fourth sets of experiments. The mean Dice score column values represent the result of the operation: reference models − proposed models.

	Compared architecture–dataset combinations	Test dataset	Type test	Test statistic (value)	p-value	Significance	Mean Dice score difference
**2ND SET OF EXPERIMENTS**		inTEST	Paired t-test	t (2.9593)	0.0081	Significant (*, p < 0.05)	0.003
	FTU-Net						
	(reference)						
	vs	colexTEST	Paired t-test	t (3.0493)	0.0066	Significant (*, p < 0.05)	0.005
	TLU-Net	exTEST	Paired t-test	t (6.5431)	2.89 x 10^−6^	Significant (*, p < 0.05)	0.006
**3RD SET OF EXPERIMENTS**	512pxData (reference)	inTEST	Paired t-test	t (1.0411)	0.3109	Not Significant	0.002
	vs	colexTEST	Paired t-test	t (−10.2376)	3.60 x 10^−9^	Significant (*, p < 0.05)	**−0.018**
	256pxData	exTEST	Paired t-test	t (6.1497)	6.54 x 10^−6^	Significant (*, p < 0.05)	0.004
	256pxData (reference)	inTEST	Paired t-test	t (13.9858)	1.87 x 10–11	Significant (*, p < 0.05)	0.05
	vs	colexTEST	Paired t-test	t (15.8559)	2.07 x 10^–12^	Significant (*, p < 0.05)	0.026
	128pxData	exTEST	Paired t-test	t (23.1271)	2.24 x 10^–15^	Significant (*, p < 0.05)	0.051
**4TH SET OF EXPERIMENTS**	512pxData (reference)	inTEST	Paired t-test	t (7.3113)	6.21 x 10^−7^	Significant (*, p < 0.05)	0.016
	vs	colexTEST	Paired t-test	t (11.4320)	5.85 x 10−10	Significant (*, p < 0.05)	0.048
	512pxGData	exTEST	Paired t-test	t (15.9909)	1.78 x 10^–12^	Significant (*, p < 0.05)	0.018
	512pxData (reference)	inTEST	Paired t-test	t (−2.0837)	0.0509	Not Significant	**−0.003**
	vs	colexTEST	Paired t-test	t (−8.4789)	6.99 x 10^−8^	Significant (*, p < 0.05)	−0.013
	512pxCLData	exTEST	Wilcoxon signed rank exact test	V (92)	0.6477	Not Significant	**0.001**
	512pxData (reference)	inTEST	Wilcoxon signed rank exact test	V (0)	1.91 x 10^−6^	Significant (*, p < 0.05)	0.076
	vs	colexTEST	Paired t-test	t (24.7979)	6.19 x 10^–16^	Significant (*, p < 0.05)	0.096
	512pxBData	exTEST	Paired t-test	t (38.1706)	2.00 x 10^–19^	Significant (*, p < 0.05)	0.053

At the instance level, FTU-Net exhibited a negligible improvement of 0.005 and a minimal decrease of 0.001 in F1 score compared to TLU-Net on the inTEST and colexTEST datasets, respectively, with no statistically significant differences in central tendency (S4 Appendix). Additionally, according to the F-test, there were no significant differences in variance in either the inTEST dataset (p-value = 0.763) or the colexTEST dataset (p-value = 0.707), indicating that both models exhibited similar behaviour. In the exTEST dataset, FTU-Net showed a minor improvement of 0.0014 over TLU-Net in F1 score, which, despite its small magnitude, was statistically significant (S4 Appendix). Therefore, although some differences in the predictive metrics of the two algorithms were statistically significant in certain test datasets, their magnitude was negligible.

### 3.2 Model performance under image-resolution downscaling

Performance metrics and test results for the third experimental set are reported in S4 Appendix, with a summary provided in Table 3. Models trained on 256pxData exhibited negligible reductions in Dice score for both inTEST (–0.002) and exTEST (–0.004), while showing an improvement in colexTEST (+0.018). However, Dice scores declined for models trained on 128pxData compared with 256pxData, with decreases of 0.05, 0.051, and 0.026 for inTEST, exTEST, and colexTEST, respectively.

A statistical analysis was conducted to assess whether reductions in image size led to significant changes in the Dice score (Table 4). The results indicate differences in the central tendencies of Dice scores in inTEST, colexTEST, and exTEST when comparing models trained on 512pxData with those trained on 256pxData, as well as when comparing 256pxData with 128pxData (Table 4). The only exception was inTEST, which showed no difference when comparing 512pxData and 256pxData, as indicated by both the t-test (p-value = 0.3109) and the F-test (p-value = 0.7097).

All models trained on the three different datasets exhibited lower precision than recall, with differences of 0.027 for 512pxData, 0.128 for 256pxData, and 0.252 for 128pxData in exTEST. The gap between these metrics became more pronounced as image resolution decreased, due to a reduction in precision and an increase in recall. At the instance level, using 256pxData instead of 512pxData led to notable increases in inTEST, colexTEST, and exTEST, with gains of 0.037, 0.087, and 0.022, respectively. These improvements were statistically significant in exTEST, inTEST, and colexTEST (S4 Appendix). Conversely, when comparing 256pxData with 128pxData, the latter showed a substantial decline in F1 score, decreasing by 0.177 in inTEST and 0.157 in exTEST, along with a slight decline of 0.067 in colexTEST. All three reductions were statistically significant (S4 Appendix).

### 3.3 Effects of using filtered imagery on model capabilities

Using the most complex architecture (FTU-Net) and the dataset with the second-highest resolution (512pxData), which was also efficient in terms of training time, as the reference system, part of the fourth set of experiments was designed to evaluate the application of different filters to the image data.

Applying a Gaussian filter to 512pxData (512pxGData) resulted in a statistically significant deterioration in Dice score across all test datasets (Table 4). Furthermore, in inTEST, exTEST, and colexTEST, the decreases were substantial, with values of 0.016, 0.018, and 0.048, respectively. Instance-level evaluation showed the same trend, with statistically significant and considerable decreases in inTEST, exTEST, and colexTEST (S4 Appendix). Regarding the use of CLAHE as a preprocessing filter (512pxCLData), at the pixel level, the observed differences in Dice score showed only a marginal improvement of 0.003 in inTEST and a slight increase of 0.013 in colexTEST when 512pxCLData was employed (Table 4). The paired t-test indicated that the models did not exhibit different central tendencies in Dice scores for inTEST (p-value = 0.0509). Additionally, no statistically significant differences in variance were found (S4 Appendix). In contrast, for exTEST, an increase of 0.001 was observed, which did not constitute a statistically significant difference (p-value = 0.6477). Instance-level evaluation indicated considerable improvements of 0.016 and 0.056 in inTEST and colexTEST, respectively, while exTEST showed only a slight improvement of 0.005 when using 512pxCLData. Statistically significant differences in the central tendency of the Dice score were found for both inTEST and colexTEST (S4 Appendix). In contrast, exTEST showed no significant differences in either central tendency or variance (S4 Appendix).

### 3.4 Performance without only one growth direction

In the fourth set of experiments, FTU-Net was also trained using 512pxBData, a dataset in which the models were trained and evaluated on increment cores exhibiting growth on both sides. Training and evaluating the architecture on 512pxBData resulted in predictions with noticeably lower Dice scores compared to the reference dataset, with differences of 0.076, 0.053, and 0.096 in inTEST, exTEST, and colexTEST, respectively. These differences were statistically significant across all test datasets (Table 4). Additionally, 512pxBData showed lower precision and recall values compared to 512pxData, indicating that the decline in performance was associated with increases in omission and commission errors (Table 3). At the instance level (S4 Appendix), statistically significant and substantial decreases of 0.141, 0.206, and 0.179 in F1 score were observed for inTEST, exTEST, and colexTEST, respectively.

### 3.5 Model performance in growth disturbance tissues

The results of the models from the second, third, and fourth experiments on the focused evaluation dataset are summarized in Tables 5, 6, and 7. Full results are provided in S4 Appendix.

**Table 5:** Pixel-level evaluation metrics for the models from the second, third, and fourth experiments, organized by dataset and architecture for the focused evaluation dataset.

			Metrics	RW	CT	IN	GS	Mean
**2ND SET OF EXPERIMENTS**	**ARCHITECTURES**	**TLU-Net**	**Dice**	0.602 + /- 0.0137	0.617 + /- 0.0142	0.591 + /- 0.0068	0.681 + /- 0.0089	0.600 + /- 0.0082
			**Precision**	0.694 + /- 0.0269	0.720 + /- 0.0312	0.635 + /- 0.0178	0.551 + /- 0.0139	0.658 + /- 0.0159
			**Recall**	0.583 + /- 0.0274	0.574 + /- 0.0269	0.597 + /- 0.0173	0.908 + /- 0.0135	0.552 + /- 0.0206
		**FTU-Net**	**Dice**	0.616 + /- 0.0110	0.634 + /- 0.0113	0.598 + /- 0.0060	0.681 + /- 0.0050	0.609 + /- 0.0067
			**Precision**	0.696 + /- 0.0153	0.717 + /- 0.0214	0.638 + /- 0.0138	0.550 + /- 0.0090	0.661 + /- 0.0112
			**Recall**	0.602 + /- 0.0197	0.597 + /- 0.0236	0.609 + /- 0.0138	0.908 + /- 0.0106	0.566 + /- 0.0165
**3 RD SET OF EXPERIMENTS**	**DATASETS**	**256pxData**	**Dice**	**0.634 + /- 0.0072**	**0.663 + /- 0.0112**	0.590 + /- 0.0063	0.648 + /- 0.0059	**0.621 + /- 0.0060**
			**Precision**	0.631 + /- 0.0229	0.679 + /- 0.0183	0.581 + /- 0.0166	0.503 + /- 0.0083	0.612 + /- 0.0138
			**Recall**	0.680 + /- 0.0191	0.679 + /- 0.0266	0.637 + /- 0.0182	**0.939 + /- 0.0081**	0.631 + /- 0.0196
		**128pxData**	**Dice**	0.556 + /- 0.0183	0.596 + /- 0.0169	0.548 + /- 0.0108	0.574 + /- 0.0112	0.538 + /- 0.0183
			**Precision**	0.481 + /- 0.0455	0.551 + /- 0.0535	0.500 + /- 0.0365	0.425 + /- 0.0161	0.462 + /- 0.0477
			**Recall**	**0.719 + /- 0.0625**	**0.694 + /- 0.0511**	**0.654 + /- 0.0564**	0.918 + /- 0.0332	**0.658 + /- 0.0555**
**4TH SET EXPERIMENTS**	**DATASETS**	**512pxGData**	**Dice**	0.588 + /- 0.0147	0.574 + /- 0.0187	0.535 + /- 0.0107	0.659 + /- 0.0071	0.565 + /- 0.0100
			**Precision**	**0.720 + /- 0.0139**	**0.738 + /- 0.0173**	**0.681 + /- 0.0105**	0.539 + /- 0.0109	**0.675 + /- 0.0079**
			**Recall**	0.559 + /- 0.0238	0.528 + /- 0.0249	0.479 + /- 0.0177	0.869 + /- 0.0120	0.487 + /- 0.0168
		**512pxCLData**	**Dice**	0.562 + /- 0.0112	0.602 + /- 0.0112	**0.600 + /- 0.0062**	**0.689 + /- 0.0047**	0.579 + /- 0.0064
			**Precision**	0.574 + /- 0.0148	0.615 + /- 0.0171	0.596 + /- 0.0087	**0.558 + /- 0.0075**	0.590 + /- 0.0107
			**Recall**	0.587 + /- 0.0151	0.613 + /- 0.0136	0.642 + /- 0.0120	0.916 + /- 0.0083	0.569 + /- 0.0108
		**512pxBData**	**Dice**	0.438 + /- 0.0127	0.499 + /- 0.0198	0.519 + /- 0.0108	0.662 + /- 0.0043	0.531 + /- 0.0087
			**Precision**	0.566 + /- 0.0180	0.636 + /- 0.0250	0.581 + /- 0.0173	0.535 + /- 0.0080	0.608 + /- 0.0100
			**Recall**	0.401 + /- 0.0174	0.460 + /- 0.0229	0.515 + /- 0.0127	0.879 + /- 0.0111	0.471 + /- 0.0131

**Table 6:** Pairwise comparisons of Dice scores for FTU-Net models across test datasets under different input datasets in the third and fourth experiments, for the focused evaluation dataset. The mean Dice score column values represent the result of the operation: reference models − proposed models.

	Compared architecture–dataset combinations	Test dataset	Type test	Test statistic (value)	p-value	Significance	Mean Dice score difference
**2ND SET OF EXPERIMENTS**	FTU-Net (reference) vs TLU-Net	FocTEST	Paired t-test	t (4.7423)	1.42 x 10^-4	Significant (*, p < 0.05)	0.009
**3RD SET OF EXPERIMENTS**	512pxData (reference) vs 256pxData	FocTEST	Paired t-test	t (−6.4518)	3.49 x 10^-6	Significant (*, p < 0.05)	**−0.011**
	256pxData (reference) vs 128pxData	FocTEST	Paired t-test	t (17.1923)	4.88 x 10^-13	Significant (*, p < 0.05)	0.083
**4TH SET OF EXPERIMENTS**	512pxData (reference) vs 512pxGData	FocTEST	Paired t-test	t (16.6647)	8.53 x 10^-13	Significant (*, p < 0.05)	0.044
	512pxData (reference) vs 512pxCLData	FocTEST	Paired t-test	t (20.1451)	2.79 x 10^-14	Significant (*, p < 0.05)	0.03
	512pxData (reference) vs 512pxBData	FocTEST	Wilcoxon signed rank exact test	V (0)	1.91 x 10^-6	Significant (*, p < 0.05)	0.079

**Table 7:** Comparative analysis of Dice scores for architecture–dataset configurations across growth-disturbance levels. The values shown in the table are the result of the operation: reference models − proposed models.

	Compared architecture–dataset combinations	RW	CT	IN	GS
**2ND SET OF EXPERIMENTS**	**FTU-Net** **(reference)** **vs** **TLU-Net**	0.013	0.017	0.007	0
**3RD SET OF EXPERIMENTS**	**512pxData (reference)** **vs** **256pxData**	**−0.018**	**−0.029**	0.008	0.032
	**512pxData (reference)** **vs** **128pxData**	0.06	0.038	0.05	0.107
**4TH SET OF EXPERIMENTS**	**512pxData (reference)** **vs** **512pxGData**	0.028	0.06	0.063	0.022
	**512pxData (reference)** **vs** **512pxCLData**	0.054	0.032	**−0.002**	**−0.008**
	**512pxData (reference)** **vs** **512pxBData**	0.178	0.135	0.079	0.019

At the global growth-disturbance level, the differences between transfer learning and fine-tuning were slight (0.009), with fine-tuning yielding a marginal improvement in Dice score (Table 6). In addition, the use of 256pxData produced a small increase of 0.011 compared with 512pxData. In contrast, when comparing 128pxData with 256pxData, a substantial decrease in Dice score was observed (0.083). All these differences were statistically significant according to the t-tests (Table 6).

At the growth-disturbance level, the use of 512pxGData and 512pxCLData resulted in considerable and statistically significant decreases in Dice score, with magnitudes of 0.044 and 0.030, respectively (Table 6). The use of 512pxBData also led to a substantial and statistically significant decrease of 0.079. The same patterns were observed at the instance level (S4 Appendix).

Table 7 shows the different responses to preprocessing depending on the specific growth disturbance. Regarding model architecture, FTU-Net produced slight improvements in Dice score for tissues affected by RW (0.013) and CT (0.017). With 256pxData, RW and CT responded positively, with improvements of 0.018 and 0.029, respectively. However, with 128pxData compared with 512pxData, RW and CT showed declines of 0.06 and 0.038 in Dice score, respectively. For both datasets, growth suppressions (GS) experienced substantial decreases in Dice score, with magnitudes of 0.032 for 256pxData and 0.107 for 128pxData.

In the fourth group of experiments, the use of 512pxGData resulted in notable performance reductions across all growth disturbances, all greater than 0.02. Likewise, with 512pxCLData, RW and CT showed clear degradations of 0.054 and 0.032, respectively, while GS and IN presented slight gains (0.008 and 0.002). Finally, the use of 512pxBData produced marked decreases in Dice score across all growth disturbances, with especially severe drops for RW, CT, and IN (0.178, 0.135, and 0.079, respectively).

### 3.6 Effects of downscaling images in densely packed tree rings

**Fig 4:**
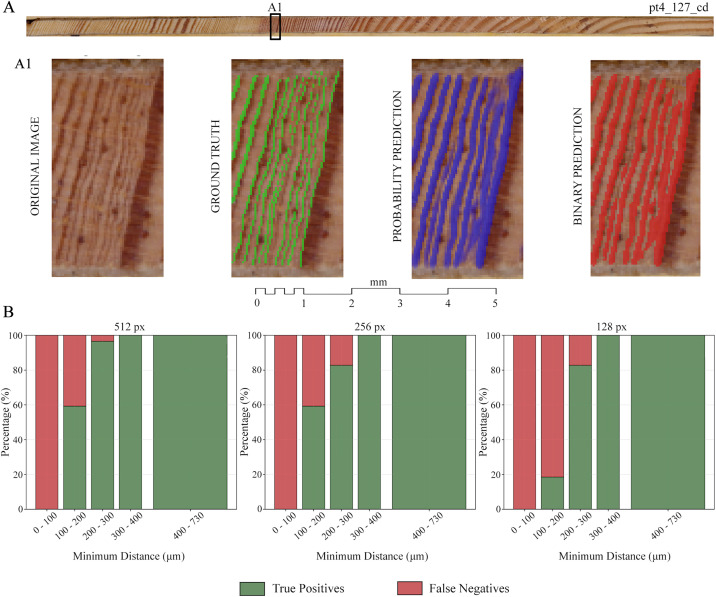
Summary of results on downscaling pre-processed images in densely packed tree rings. **(a)** One example of artefacts created by the downsampling. **(b)** Histogram comparison between datasets.

The results of the instance evaluation for the different combinations of FTU-Net with 512pxData, 256pxData, and 128pxData are presented in Fig 4; the raw information is also provided in S5 Appendix. This section shows the results for the 10 images of growth reduction belonging to the focused evaluation dataset.

One of the effects of downscaling is the alteration of the ground-truth mask labels in the dataset. When examining the 10 growth-suppression samples in the focused evaluation dataset, it was observed that 512pxData and 256pxData were free of such artefacts, whereas four images in 128pxData displayed them. As shown in Fig 4, downscaling induces gaps between tree-ring instances or causes rings that were originally very close to each other to merge. All these issues in the four affected images occur in growth-suppression regions where certain tree rings with inter-ring distances below 250 µm are present.

Fig 4 also shows that recall for distances below 300 µm is zero, while for distances above 300 µm, all configurations achieve perfect recall. In the 100–200 µm range, recall reaches 0.592 for both 512pxData and 256pxData, whereas 128pxData shows a markedly lower value of 0.182. In the 200–300 µm range, recall is 0.965 for 512pxData and 0.827 for both 256pxData and 128pxData. Beyond 300 µm, recall reaches 1.0, which is unsurprising given that the growth-suppression samples were selected to minimise confounding sources of noise, such as abrupt colour transitions.

## 4 Discussion

### 4.1 Identification of the reference models

The first and second sets of experiments were designed to determine the appropriate level of architectural complexity and the optimal degree of cellular detail at which the models should operate. Using the first set, our results indicated that the custom U-Net models demonstrated lower performance compared to the pre-trained U-Net models. This discrepancy is likely attributable to the design of the custom U-Net architectures, which were optimized for processing small patches of the increment core containing a limited number of tree rings [39]. Consequently, they may have lacked sufficient capacity to effectively handle full-height core images, which contain more information and potential sources of noise. Additionally, in this experiment, the cellular detail was greater than in the study by Fabijańska and Cahalan [39]. However, as shown in Tables 3 and 4, the custom U-Net models demonstrated a slight improvement when image resolution was maintained. This observation contradicts the initial assumption that higher-resolution images would introduce excessive noise for a simple neural network to handle effectively.

A further observation was that U-Net did not fully capture contextual information [72,73]. This may be related to the fact that modifications of U-Net, such as attention gates and residual convolutional blocks, led to improvements in Dice scores (S4 Appendix). Together, these findings suggest that there is potential to outperform the large architectures currently applied in tree-ring segmentation research [12,37,38,40,41], including the pre-trained U-Net used in this study. Higher performance could potentially be achieved by slightly increasing the number of filters in the convolutional layers of the architecture proposed by Fabijańska and Cahalan [39] or by incorporating additional modifications, such as adding a transformer component [73]. Our results indicate that, for our datasets, the pre-trained U-Net architecture achieved better performance than the simpler architecture proposed by Fabijańska and Cahalan [39], as long as the number of parameters in the latter remained unchanged. Moreover, based on the first set of experiments, the use of the 1024pxData resolution—which preserves nearly the full resolution of all increment core images (S1 Table)—did not lead to improved predictive performance (Table 4 and S4 Appendix) and in some cases even produced worse results. In addition, it requires substantially greater computational resources (S3 Appendix), which would considerably increase the cost of its use in subsequent experiments. Therefore, this resolution was discarded, and 512pxData was selected as the reference.

From the second set of experiments, it is observed that the performance of the pre-trained U-Net models falls within the range of F1 scores reported in previous tree-ring segmentation studies (Table 8), where Mask R-CNN architectures or enhanced U-Net variants were applied [12,37,38,40]. In this set of experiments, U-Net with transfer learning and U-Net with fine-tuning showed similar performance, indicating that adapting more complex features to this dataset did not noticeably improve the ability to segment tree-ring borders. This suggests either that the proposed pre-trained U-Net architectures are already operating near their performance ceiling or that further improvements may depend on refining lower-level features. Given its slightly superior performance and comparable prediction time, FTU-Net was selected as the reference architecture.

**Table 8:** Instance segmentation results and evaluation metrics in tree-ring segmentation studies. N trained models indicates the number of models of the proposed architecture trained and evaluated.

	N trained models	IoU Overlapped criteria (%)	Precision	Recall	F1
**Marichal and Randall** [35]	1	–	0.755	0.797	0.775
**Wu et al.** [37]	1	25	0.6592	0.8527	0.7348
**Kim et al.** [38]	1	90	–	0.905	–
**Fabijańska and Cahalan** [39]	3	–	**0.99**	0.92	0.9537
**Poláček et al.** [40]	1	50	0.96	**0.98**	**0.9699**
**FTU-Net with 512pxData**	20	50	0.663 + /- 0.0096	0.780 + /- 0.0067	0.716 + /- 0.0078

### 4.2 Visual evaluation of the reference model on increment cores

**Fig 5:**
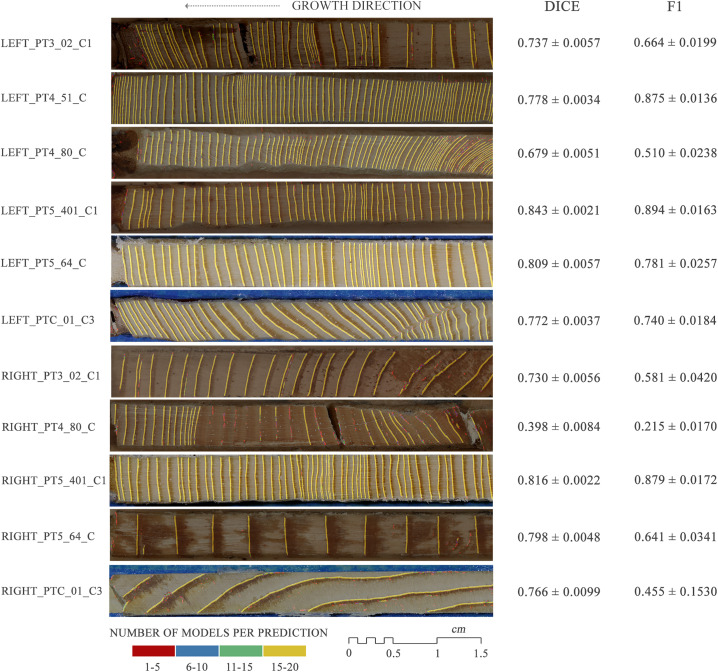
Visual results on tree-ring boundary segmentation and per-image Dice coefficient and F1 score mean and standard deviation for the FTU-Net model across exTEST. The colour mapping shows the range of ring border predictions, based on the sum of models without height-based filtering.

**Fig 6:**
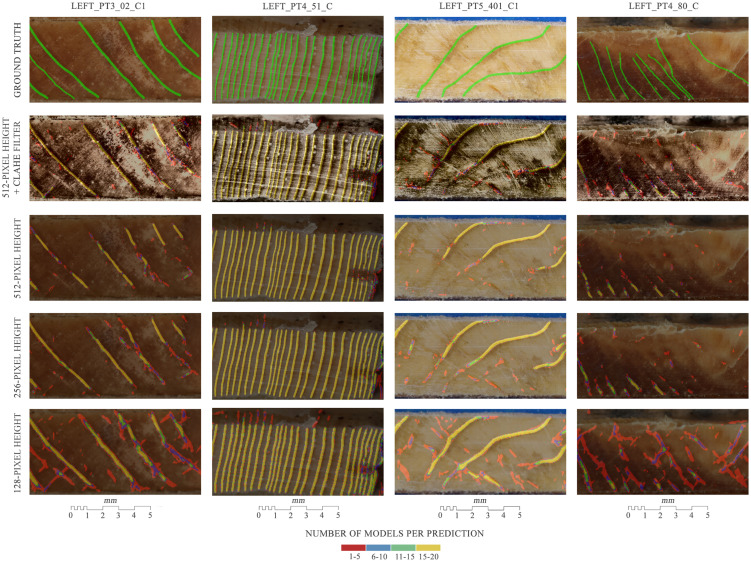
Segmentation of rings in abnormal growth patterns performed by fine-tuned U-Net models trained on datasets with different heights and filters in exTEST. The colour mapping shows the range of ring border predictions, based on the sum of models without height-based filtering.

The FTU-Net models trained on 512pxData demonstrated competent segmentation performance in detecting tree-ring borders within normal growth patterns, generating continuous and well-separated entities (Fig 5). However, in abnormal growth patterns—particularly when the contrast between latewood and earlywood was very subtle or in the presence of growth disturbances such as callus tissue or compression wood—the model segmented only partial borders or none at all (Fig 6). Furthermore, the presence of dark brown stains crossing the tree rings horizontally (see example LEFT_PT4_51_C in Fig 6) generally hindered the generation of continuous segmented entities. Nevertheless, when the stain crossed the latewood to a lesser extent and its colouration was lighter, segmentation was more likely to remain continuous.

**Fig 7:**
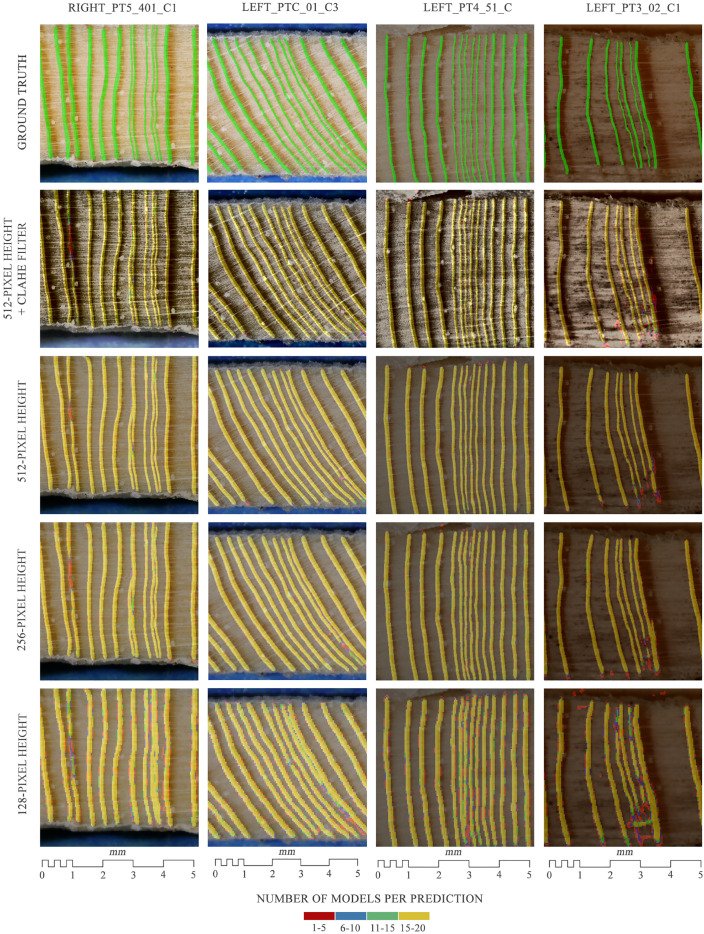
Segmentation of the most closely spaced rings performed by fine-tuned U-Net models trained on datasets with different heights and filters in the external test dataset. The colour mapping shows the range of ring border predictions, based on the sum of models without height-based filtering.

Regarding sets of narrow tree rings, the models consistently demonstrated the ability to predict continuous and well-separated tree-ring borders when no additional sources of noise were present (512-pixel height in Fig 7). As shown in Fig 5, a higher prevalence of abnormal growth patterns was associated with lower Dice and F1 scores.

### 4.3 Influence of image resolution on segmentation performance

This subsection addresses RQ1 by examining how variations in image resolution and cellular detail influence segmentation performance. Macroscopic studies of tree-ring segmentation have revealed a wide range of cellular detail, with cell boundaries clearly visible [40], moderately visible [39], or not visible [12,35,37,38]. Generally, image quality is positively correlated with the performance of CNNs [74,75]. However, in many machine learning paradigms, reducing the number of inputs or features is often preferred, as it decreases the number of parameters to be optimized and simplifies the model [76,77]. The use of highly detailed images, while potentially beneficial, can increase model complexity, which may necessitate a more sophisticated architecture.

Neural networks with a very large number of layers often face the challenge of higher training error (loss) compared with their shallower counterparts, as the growing number of parameters makes optimization less tractable [59,76]. This is consistent with our results from the first set of experiments, where pre-trained U-Net models showed worse performance when maintaining full image resolution (1024pxData) compared with models using downsampled data. In this context, detailed information could be better exploited by architectures incorporating mechanisms such as Attention U-Net, which improve segmentation performance [33,59]. However, increasing image size and architectural complexity involves trade-offs, such as GPU memory limitations. These constraints often necessitate a reduction in batch size, which can negatively impact gradient estimation for the loss function and, ultimately, model performance [76].

The performance of CNNs, beyond image resolution, depends on the visual information and specific characteristics of the predicted object [75,76]. For instance, Sabottke and Spieler [76] found that different diagnostic tasks required different optimal image sizes, and Boom et al. [78] did not identify the highest available resolution as optimal. In contrast, Thambawita et al. [79] achieved peak segmentation performance using the highest resolution. The results from the first and third sets of experiments showed that the segmentation system remained robust to image downsampling, with the main impact observed in the segmentation of densely packed tree-ring borders.

In the fourth set of experiments, applying a CLAHE filter enhanced cellular detail but did not notably improve model performance. However, better performance was observed in colexTEST, suggesting that this filter could be beneficial when images exhibit varying illumination conditions. Furthermore, a Gaussian blur filter was applied, blurring both cellular details and the contrast between earlywood and latewood, which led to a notable deterioration in model performance. By contrast, downsampling removed a substantial amount of information but preserved contrast between wood types. However, reduced resolution gradually compresses sets of narrow tree rings, diminishing the model’s ability to correctly predict tree-ring borders within these sets, with the minimum consistently detectable distance increasing from 200 μm to 300 μm.

It is important to note that the results obtained with 128pxData may be limited by the fact that the original 512pxData labels were downsampled to create 128pxData, introducing artefacts in densely packed tree rings. Nevertheless, this limitation reinforces the notion that neural networks trained on this dataset could achieve even better performance if annotations were generated directly at that resolution.

Quantitatively, it was shown that the loss of cellular detail in 128pxData and 256pxData did not lead to a substantial reduction in segmentation performance when analyzing increment cores. Deep neural networks exploit the hierarchical structure of many natural signals, in which high-level representations arise from the composition of simpler, lower-level elements [25]. In images, small edge patterns combine into local motifs; these motifs assemble into object parts, and the parts ultimately form complete objects [25]. Therefore, if 512pxData enables the neural network to capture cellular information in the first encoder layer, this indicates that the model is making use of cellular detail during processing. Conversely, in 128pxData and 256pxData, where cellular structures are not present, the extraction of such features is not possible.

**Fig 8:**
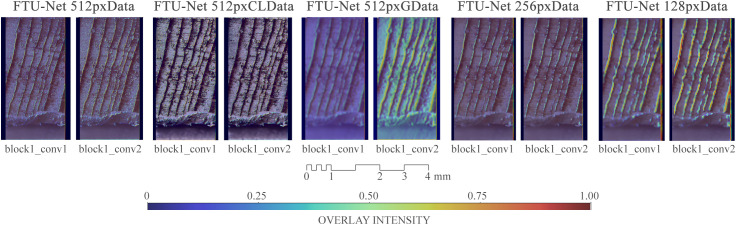
Seg-Grad-CAM applied in GS_2 by different architecture-dataset combinations in the first block of the convolutional decoder. **(a)** FTU-Net 512pxData **(b)** FTU-Net 512pxCLData **(c)** FTU-Net 512pxGData **(d)** FTU-Net 256pxCLData **(e)** FTU-Net 128pxCLData.

The Seg-Grad-CAM analysis, presented in S6 Appendix, uses heat maps that highlight the relevance of individual pixels [70], providing further insight into whether different levels of detail are exploited in 512pxData. As shown in Fig 8, when cellular information is available, as in 512pxData and 512pxCLData, the models effectively leverage these features in addition to relying on the simpler colour-contrast cue. By contrast, 512pxGData, 256pxData, and 128pxData, which lack cellular detail, do not extract information of this type. Thus, although 512pxData and 512pxCLData make use of fine-grained morphological features, quantitative evaluation indicates that the performance improvement is not substantial.

The generally maintained segmentation capacity despite downsampling suggests that when CNNs are optimized to rely primarily on simple features, such as colour gradients, they can achieve performance comparable to CNNs that also exploit more complex cellular structures. This indicates that, at the macro scale, capturing cellular-level detail may not be necessary to accurately segment tree rings spaced at approximately 300 μm.

### 4.4 Implications of using non-standardised growth directions

**Fig 9:**
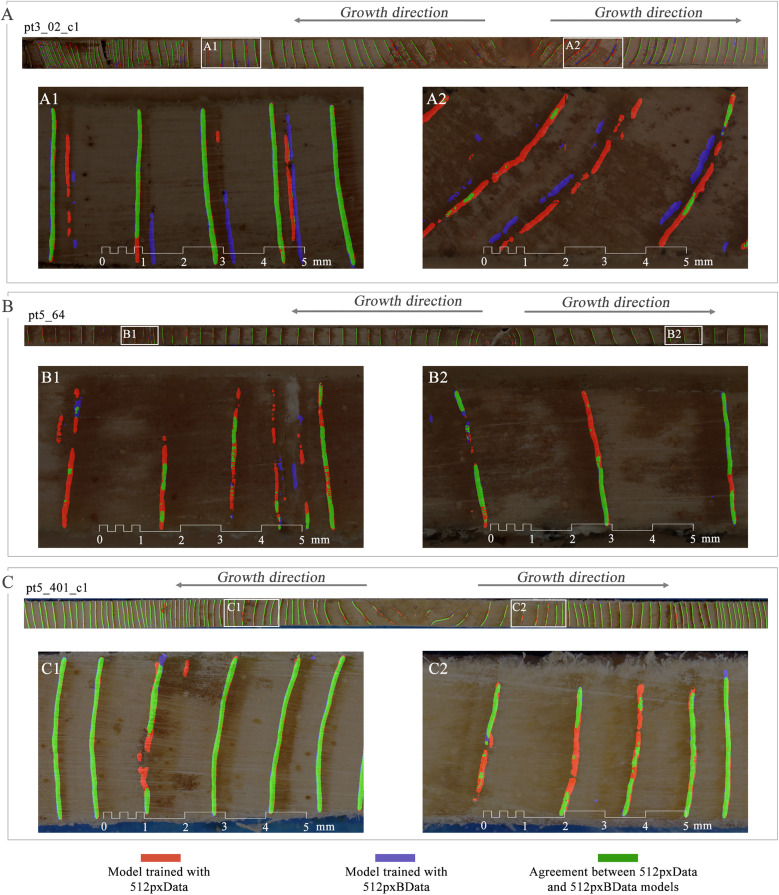
Examples of predictions from a model trained with 512pxData and another trained with 512pxBData. Comparison using one model per architecture-dataset combination as an example. The predictions were binarized using a 0.5 threshold. Green pixels indicate agreement between both models, red pixels correspond to regions detected only by the model trained on 512pxData, and blue pixels to regions detected exclusively by the model trained on 512pxBData. Predictions were generated without height-based filtering.

The fourth experiment was designed to test whether cellular resolution and tree-ring convexity could efficiently detect the growth direction of an increment core, an aspect directly connected to RQ2. Previous studies [12,35,36] have successfully segmented tree rings in tree cross-sections, where the direction of growth is multidirectional. In contrast, our models produced significantly worse results when growth-direction information for increment cores was not provided beforehand. We believe that the model of Ge et al. [12] achieved superior results because it used the central position of the pith as a reference for growth direction. This interpretation is supported by other studies [35,36], in which one of the CNNs in their intelligent system is tasked with detecting the pith in cross-sections. In our system, which relied on patches, the convexity of tree rings and the cellular detail were insufficient. Quantitatively, the use of 512pxBData led to notable reductions in segmentation performance (Tables 5 and 7). Fig 9 shows that the primary limitation of models trained with 512pxBData is their inability to fully segment tree-ring boundaries, resulting in discontinuities along the predicted rings. In addition to this main issue, these models also display a greater tendency—compared with those trained on 512pxData—to misclassify colour transitions, such as random dark stains in earlywood or the gradual transition from earlywood to latewood, as tree-ring borders.

### 4.5 Performance of CNN-based system in abnormal growth patterns

Except for Ge et al. [12], previous studies have not focused on tree-ring segmentation under growth anomalies. Our analysis in this section addressed RQ3 through the use of increment cores from trees affected by growth disturbances and the evaluation of the system using the focused evaluation dataset. Among these disturbances, very narrow rings were particularly challenging due to their proximity. With 512pxData, we obtained consistent predictions for inter-ring distances down to 200 μm. Below this threshold, errors progressively increased until 100 μm, where no accurate predictions were achieved. These issues have been reported in other studies [38–40], where different segmentation instances merged due to proximity. Since those studies did not provide quantitative distance measures, we are unable to compare their capabilities quantitatively.

These studies [38,40] used the Mask R-CNN architecture, which is not as specialized for fine pixel-level segmentation as U-Net architectures, and Fabijańska and Cahalan [39] had to merge small patch predictions in both directions due to these challenging situations. Wu et al. [37] did not analyse sets of narrow tree-ring packages, while Ge et al. [12] claimed to achieve separate segmentation; however, their figures show incorrectly merged tree-ring instances, likely due to the small image size used. The system proposed here, based on U-Net and a sliding window moving along a single axis, consistently segmented tree rings with distances above 200 μm, correctly identifying most closely spaced rings in this dataset. Thus, regarding growth disturbances, the proposed system performs well in cases of growth suppression, except in extreme cases involving very closely spaced rings. Additionally, the system performs well in detecting growth release, as it did not pose a technical challenge.

**Fig 10:**
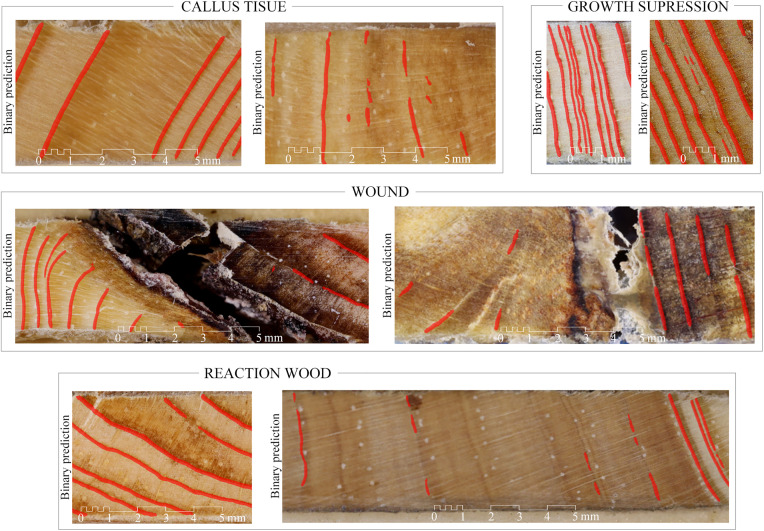
Examples of binary predictions produced by FTU-Net 512pxData for two samples of each growth-disturbance category in the focused evaluation dataset. Predictions were generated without height-based filtering.

Following the analysis of growth disturbances observed throughout our experiments, RW, CT, and IN exhibited much lower recall values than growth suppression, resulting in markedly worse Dice scores (Table 5). We found that when the termination of the RW or CT zones was well defined and the earlywood of the following year was clearly distinguishable, the tree-ring boundary was segmented accurately (Fig 10). Nonetheless, RW and CT typically extend over several years, and under these conditions the models performed poorly (Fig 10). This suggests that tree rings affected by these disturbances, characterised by changes in colour and texture, are more difficult for the models to detect completely. In the case of injuries, the challenge is even greater, as the disturbed tissue presents more pronounced and irregular patterns. Previous studies [35,37–39] reported issues with abnormal growth patterns or directly avoided labelling those tree-ring borders, while Ge et al. [12] claimed success, although concerns about segmentation under abnormal growth patterns arise when inspecting their figures.

Our study contributes to advancing the focused exploration of tree-ring segmentation in tissues affected by growth disturbances. One contribution is that growth suppression, which does not introduce additional noisy signals, can be effectively handled by our proposed system within our dataset, although testing in other regions or species remains unexplored. We also show that the relative proportion of tree rings affected by growth disturbances with respect to the total number of rings in a tree was insufficient to achieve satisfactory performance in RW, CT, and IN. Different studies work with different species that present varying textures and colouration. Yet, CNNs still struggle to adapt in cases where there is no clear colour gradient. We believe that a successful strategy for segmenting tree rings under abnormal growth patterns could involve training CNNs exclusively on sections of increment cores affected by growth disturbances, thereby allowing the network gradients to focus on minimizing this specific challenge. Another valid approach would be to use image patches extracted exclusively from rings exhibiting growth disturbances, increasing the proportion of affected rings relative to that used in the present study (Table 1).

## 5 Limitations and future perspectives

Different limitations were identified in this work, and several future research directions were also recognized. First, our proposed model does not incorporate common U-Net enhancements such as Attention Gates [33], residual convolutional blocks [58], transformer components [73], which could better exploit available information. Related work has also demonstrated that attention-driven encoder–decoder frameworks and refined feature-aggregation strategies can substantially improve segmentation robustness under complex imaging conditions [80]. Moreover, the simpler model proposed from scratch by Fabijańska and Cahalan [39] performed not far below our model, despite having a substantially smaller number of parameters. Future work could therefore focus on achieving higher performance either by slightly increasing the number of filters in the convolutional layers of the architecture proposed by Fabijańska and Cahalan [39], by integrating architectural modifications into our model [73], or by exploring instance-segmentation approaches such as Mask R-CNN [81]. Future work could also explore transformer-based architectures, such as Vision Transformers (ViT), which have shown strong potential for capturing long-range contextual information in image segmentation tasks [82].

Another limitation of this study is that, due to computational constraints, we did not further experiment with 1024pxData, which, according to the original image sizes (S1 Table), preserves the resolution of most images. This decision was made because the evaluation metrics did not show improved performance when using this dataset. Nonetheless, as noted previously, modifications to the U-Net architecture could potentially allow the model to better exploit the larger amount of information contained in higher-resolution images. One of our goals was to evaluate whether using increment cores containing growth disturbances would be sufficient for the model to perform well. As shown, the proportion of disturbed to regular tree rings plays an important role. A promising direction for future research would be to investigate whether incorporating image patches specifically containing disturbed rings could improve model performance, or even to develop a dedicated system designed exclusively for segmenting disturbed tree rings.

The annotation protocols reveal several open challenges that warrant further investigation, including: (a) evaluating model performance on broken tree rings; (b) detecting false tree rings; and (c) identifying extremely difficult tree rings obscured by colour variations in growth-disturbed sections. The first challenge could be addressed using a specific dataset designed for segmentation based on the position and size of the hole, while the latter two challenges could potentially be mitigated with the assistance of cross-dated series. Additionally, a limitation of our study is that manual labelling of tree rings was performed after downsampling the original resolution, which introduced artefacts at very low resolutions. Therefore, a fairer comparison remains pending, ideally involving manual annotation carried out directly at those low resolutions. Moreover, our study did not address the segmentation of very small-height tree rings, particularly those affected by breakage or highly horizontal orientations near the pith.

Although in this study we explored growth disturbances generated by most geomorphological processes occurring in medium- and high-mountain environments [4,5,7,8], another broad limitation concerns species generalization, since we examined only a single species. Previous literature has shown that model performance can vary across species [37,39,38]. Nonetheless, Poláček et al. [40] demonstrated that even when trained on a single species, the consistent anatomical structure of conifer wood can lead to comparable performance in other conifer species. These studies focused on non-disturbed tissues and included multiple species in their training and testing procedures, or at least in the testing phase. When considering the generalization of tree-ring segmentation in disturbed tissues, an additional source of variability emerges, as the species-specific response to a given disturbance may introduce further heterogeneity. Nonetheless, the majority of dendrogeomorphological studies rely on species that display clear and easily identifiable growth disturbances [9]. Incorporating these commonly used species in future work could help further assess the model’s capacity for generalization within this type of study. This remains an open question that warrants further investigation.

With respect to the use of standardised growth directions in a patch-based system, it would be valuable to explore growth-direction sensitivity in greater depth. In particular, future work could investigate the incorporation of simple priors, such as lightweight orientation classifiers or pith-localisation proxies, and assess whether the limited context window inherent to patchifying constrains the inference of growth direction by varying patch width and receptive field. As one of our main recommendations is to increase the number of tree rings from growth-disturbance tissues, we acknowledge that this may not be straightforward and that data scarcity could remain a challenge. In such a scenario, an interesting alternative would be to explore lightweight CNN architectures, traditional feature extraction combined with machine-learning methods, or architectures specifically designed for small datasets, such as Union-Net [83].

## 6 Conclusions

This work provides valuable insights into the field of tree-ring segmentation, particularly under growth-disturbance conditions, by analysing a highly disturbed dataset and evaluating different deep-learning architectures with varying levels of cellular detail. At a macro level, the findings show that U-Net struggles to delineate rings in disturbed tissues associated with colour changes and textures such as callus tissue, reaction wood, and injuries, regardless of network complexity or cellular detail. Nevertheless, in growth-suppression disturbances characterised by very closely spaced rings, the models perform successfully in areas free of other growth-disturbance noise, consistently detecting rings spaced approximately 200 µm apart in zones corresponding to the strongest growth reductions. This segmentation ability decreases as image resolution is reduced, lowering the model’s capacity to reliably detect rings with spacing up to 300 µm.

These findings indicate that, while the proposed system is suitable for addressing growth-suppression scenarios, additional or alternative approaches are required to fully capture other types of growth anomalies. For regular-growth tissues, segmentation performance remains resilient to image downsampling, as long as the colour gradient between rings remains clear and the models are optimized using downsampled images, while still achieving good results when the networks rely primarily on simple image features. This may be valuable for researchers who are not focused on densely packed tree rings, as they may save time by using lower-resolution images and reallocating resources to other tasks.

An additional finding is that, when working with increment cores and applying the patchify process, it was not possible to reliably infer the direction of growth, which in turn worsened overall system performance. This study provides a promising initial exploration of deep learning for segmenting tree rings in disturbed trees, revealing the potential of this approach while emphasizing the substantial work still needed to fully accomplish this task.

## Supporting information

S1 Fig Example of LEFT_PT5_401_C1 with different height resized. (a) 128-pixel height resized image. (b) 256-pixel height resized image (c) 512-pixel height resized image (d) 1024-pixel height resized image (e) 834-pixel height original image.
(ZIP)

S2 Fig Example of LEFT_PT4_02_C1 with different filters applied. (a) CLAHE filter. (b) Blur filter. (c) Raw image.
(PDF)

S3 Fig Experimental design scheme. In green, the preprocessing steps performed, and in purple, the partitioning strategies implemented.
(PDF)

S1 Table Summary characteristics of the increment cores in primary dataset. It contains quantiles of the original image dimensions (in pixel units) and the number of tree rings per core.
(XLSX)

S2 Table Measurements of tree ring distances in GS images of focused evaluation dataset. This file contains the processed measurements of tree-ring distances. All measurements are reported from the oldest tree ring to the youngest. Field descriptions: gs: Name of the corresponding image. id: Identifier assigned to each ring, numbered sequentially from the oldest year. dist_to_next: Distance from each ring to the next one in chronological order, measured in µm/10. mindist: Closest neighboring ring on either side, used for evaluation, measured in µm/10.
(XLSX)

S3 Table Preprocessing applied to the seven datasets used in the study. (XLSX)

S4 Table Table with percentile values of relative ring heights with respect to image height. Derived from the primary dataset. The mean and median values are 0.729 and 0.740 respectively.
(XLSX)

S5 Table Summary table of the parameter values adjusted for each algorithm-dataset combination. (XLSX)

S1 Appendix Graphic information about the fieldwork trip. (a) Zone map showing the sampled trees, including the 64 used for tree ring segmentation. (b) Sampled tree with a significant ramification near the soil. (c) Sampled tree with a scar on the trunk. (d) Sampled tree with trunk curvature.
(PDF)

S2 Appendix Expanded justification of the criteria used to select the parameters for the Seg-Grad-CAM algorithm. (PDF)

S3 Appendix Information on the number of epochs, prediction times, and training times required by the models. This supplementary folder provides the number of epochs, prediction times, and training times required by the models evaluated in the experiments. It is organized into the following subfolders: epochs_used folder-----------------. The file “epochs_by_partition” contains the number of epochs used by each model. The file “epochs_statistics_by_file” summarizes these values grouped by experiment–architecture–dataset combination. predict_times folder---------------. The file “prediction_times_summary” reports the prediction time for each model. The file “prediction_times_statistics” provides aggregated values grouped by experiment–architecture–dataset combination. The remaining files include additional comparative data used in the study. train_times folder----------------. The file “training_times_summary” reports the training time for each model. The file “training_times_statistics” provides aggregated values grouped by experiment–architecture–dataset combination. The remaining files include additional comparative data used in the study. The prediction times correspond to those obtained using a system equipped with a 13th Gen Intel® Core™ i7-13620H processor (2.40 GHz) and 16 GB of RAM.
(ZIP)

S4 Appendix Information on instance-level and pixel-level metrics for the models used. In this folder, the instance and pixel metrics are provided using the following codes: Models: m0 - CU-Net. m1 - CAU-Net. m2 - CARU-Net. m3 - TLU-Net. m4 - FTU-Net. Datasets: d0C - 512px Data. d1C - 512px GData. d2C - 512px CLData. d7C - 512px BData. d10C - 1024px Data. d11C - 256px Data. d14C - 128px Data.
(ZIP)

S5 Appendix Information on tree-ring prediction in growth suppressions. The supplementary material contains two main folders: -plots: This folder includes, for each image of the GS studied in the focused evaluation dataset, the original image, the labels, the probability prediction (mean value of the 20 trained models), and the binary prediction (results obtained by applying a 0.5 threshold to the probability prediction). -results: This folder contains two files. results_table: A table including the distance measures (measured in µm/10) described in S2_Table. The column “tp_512px” corresponds to the true positives for the 512pxData. A value of 1 indicates a true positive, and a value of 0 means the ring was not correctly detected. histogram_results: A table containing the data used to generate Fig 4b.
(ZIP)

S6 Appendix Results of Seg-Grad-CAM in GS_2. (ZIP)
