# Supplementary material for: Tree ring segmentation performance in highly disturbed trees using deep learning
Source: PLoS One. 2026 Jun 18;21(6):e0321841. doi: 10.1371/journal.pone.0321841 (PMC13278439; doi:10.1371/journal.pone.0321841)
Supplement: S1 Fig — (a) 128-pixel height resized image. (b) 256-pixel height resized image (c) 512-pixel height resized image (d) 1024-pixel height resized image (e) 834-pixel height original image. (ZIP) [file pone.0321841.s001.zip › S1_Figure.pdf]

Tree ring segmentation performance in highly disturbed trees using deep learning

Joe David Zambrano-Suárez[1,2], Jorge Pérez-Martín[3], Alberto Muñoz-Torrero Manchado[1], Juan Antonio Ballesteros Cánovas[1]**

1 Department of Geology, National Natural Science Museum, Spanish Research Council, Madrid, Spain
2 Research Institute of Water and Environmental Engineering, Universitat Politècnica de València, Spain
3 Department of Artificial Intelligence, National University of Distance Education, Madrid, Spain

**joe.zambrano@mncn.csic.es / juan.ballesteros@mncn.csic.es*

S1 FIGURE

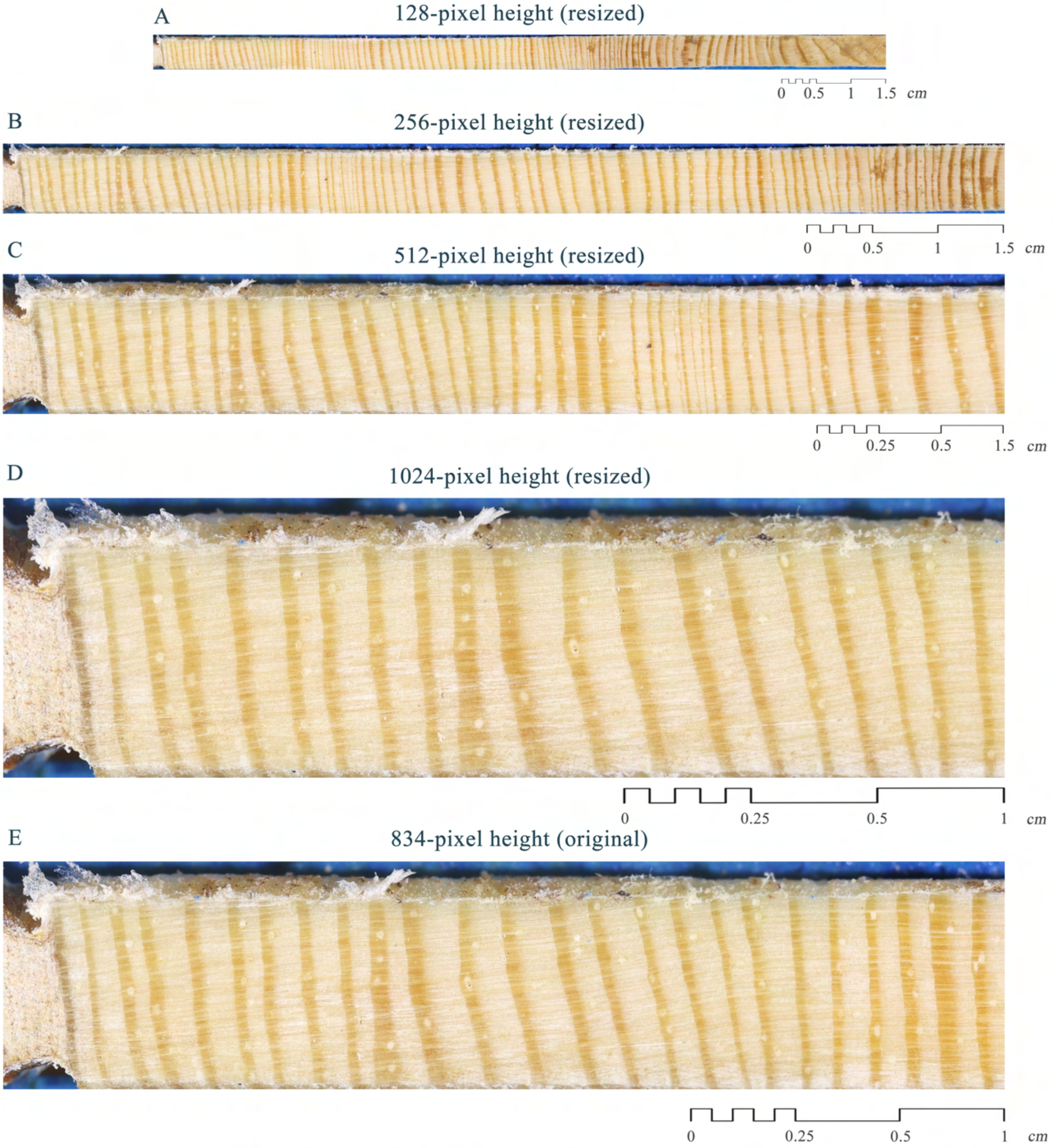

S1 Figure. Example of left_pt5_401_c1 with different height resized. (a) 128-pixel height resized image. (b) 256-pixel height dataset. (c) 512-pixel height resized image. (d) 1024-pixel height resized image. (e) 834-pixel height original image.
