# Supplementary figures and images for: Tree ring segmentation performance in highly disturbed trees using deep learning
Source: PLoS One. 2026 Jun 18;21(6):e0321841. doi: 10.1371/journal.pone.0321841 (PMC13278439; doi:10.1371/journal.pone.0321841)

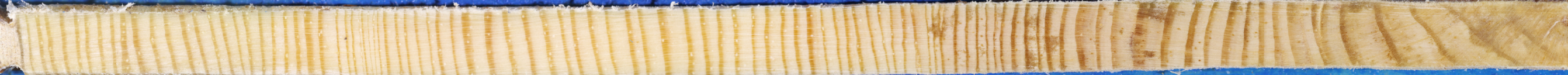

Supplement: S1 Fig — (a) 128-pixel height resized image. (b) 256-pixel height resized image (c) 512-pixel height resized image (d) 1024-pixel height resized image (e) 834-pixel height original image. (ZIP) [file pone.0321841.s001.zip › a.png]

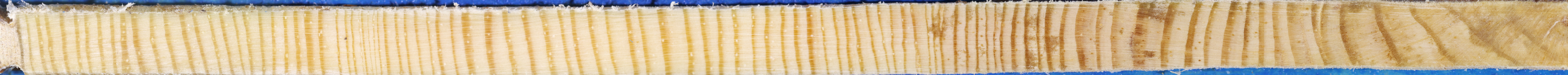

Supplement: S1 Fig — (a) 128-pixel height resized image. (b) 256-pixel height resized image (c) 512-pixel height resized image (d) 1024-pixel height resized image (e) 834-pixel height original image. (ZIP) [file pone.0321841.s001.zip › b.png]

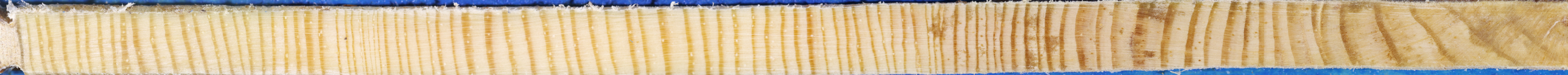

Supplement: S1 Fig — (a) 128-pixel height resized image. (b) 256-pixel height resized image (c) 512-pixel height resized image (d) 1024-pixel height resized image (e) 834-pixel height original image. (ZIP) [file pone.0321841.s001.zip › c.png]

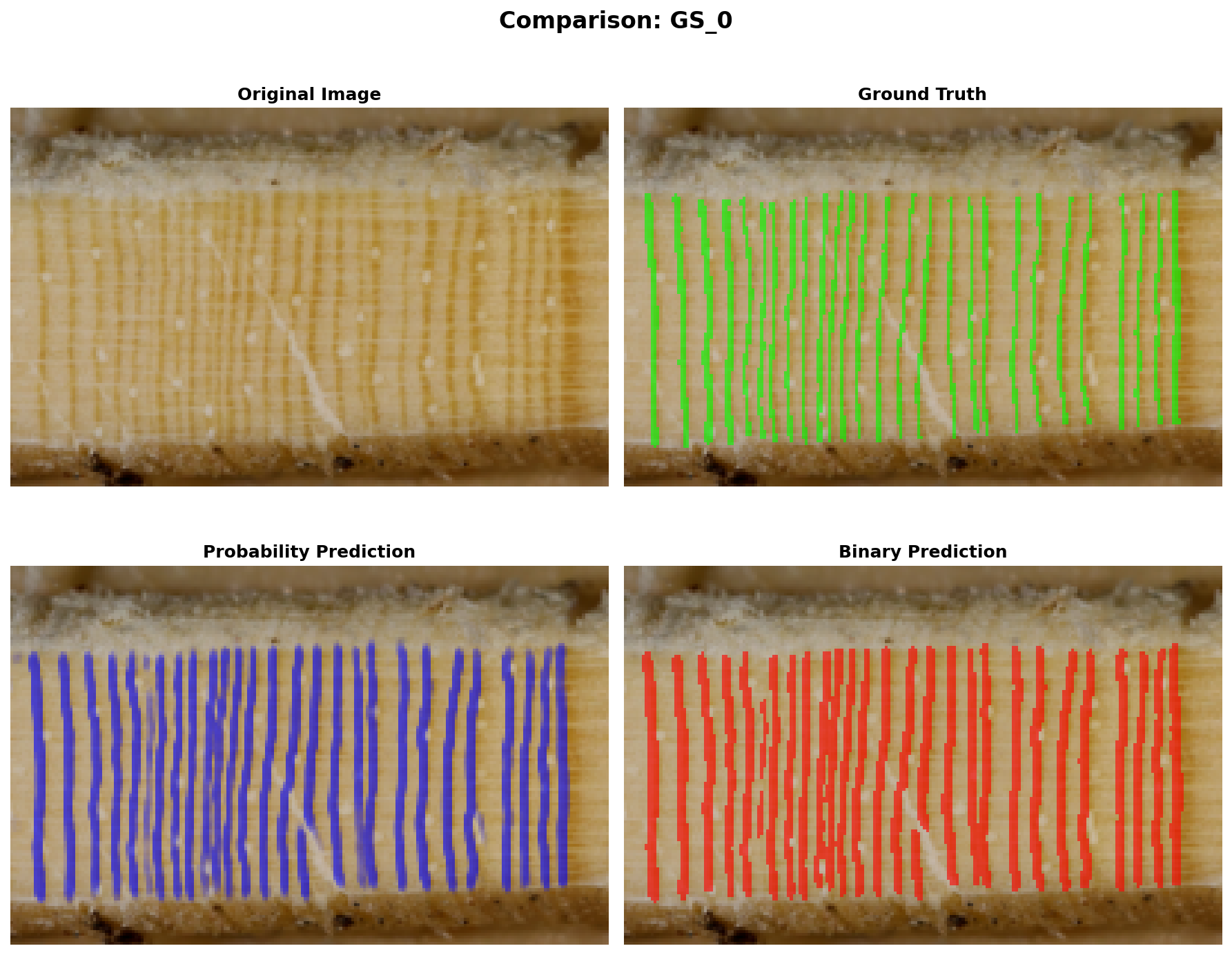

Supplement: S5 Appendix — The supplementary material contains two main folders: -plots: This folder includes, for each image of the GS studied in the focused evaluation dataset, the original image, the labels, the probability prediction (mean value of the 20 trained models), and the binary prediction (results obtained by applying a 0.5 threshold to the probability prediction). -results: This folder contains two files. results_table: A table including the distance measures (measured in µm/10) described in S2_Table. The column “tp_512px” corresponds to the true positives for the 512pxData. A value of 1 indicates a true positive, and a value of 0 means the ring was not correctly detected. histogram_results: A table containing the data used to generate Fig 4b. (ZIP) [file pone.0321841.s013.zip › plots/gs_plots_im_gt_predprob_predbin_128px/GS_0_composite.png]

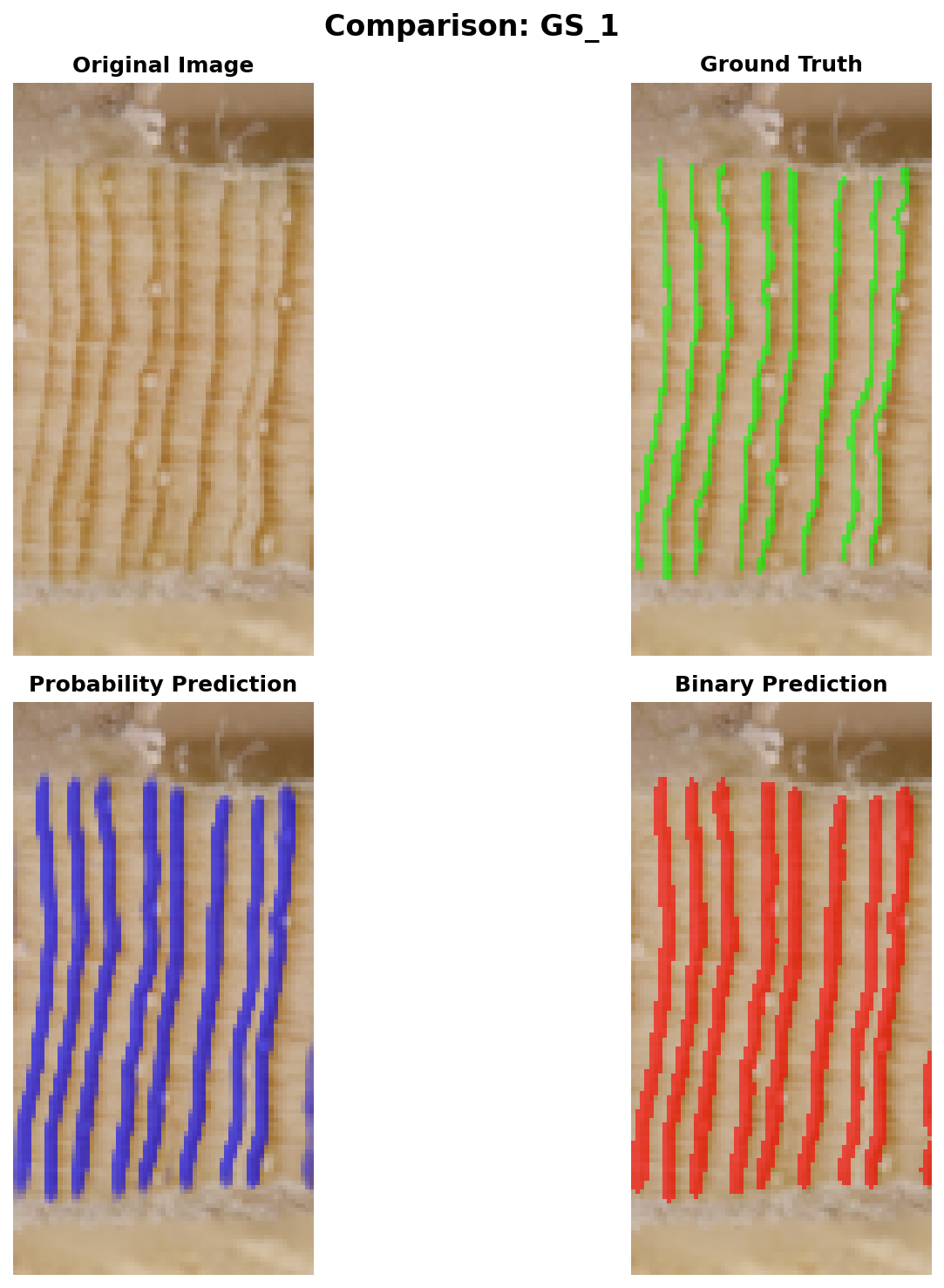

Supplement: S5 Appendix — The supplementary material contains two main folders: -plots: This folder includes, for each image of the GS studied in the focused evaluation dataset, the original image, the labels, the probability prediction (mean value of the 20 trained models), and the binary prediction (results obtained by applying a 0.5 threshold to the probability prediction). -results: This folder contains two files. results_table: A table including the distance measures (measured in µm/10) described in S2_Table. The column “tp_512px” corresponds to the true positives for the 512pxData. A value of 1 indicates a true positive, and a value of 0 means the ring was not correctly detected. histogram_results: A table containing the data used to generate Fig 4b. (ZIP) [file pone.0321841.s013.zip › plots/gs_plots_im_gt_predprob_predbin_128px/GS_1_composite.png]

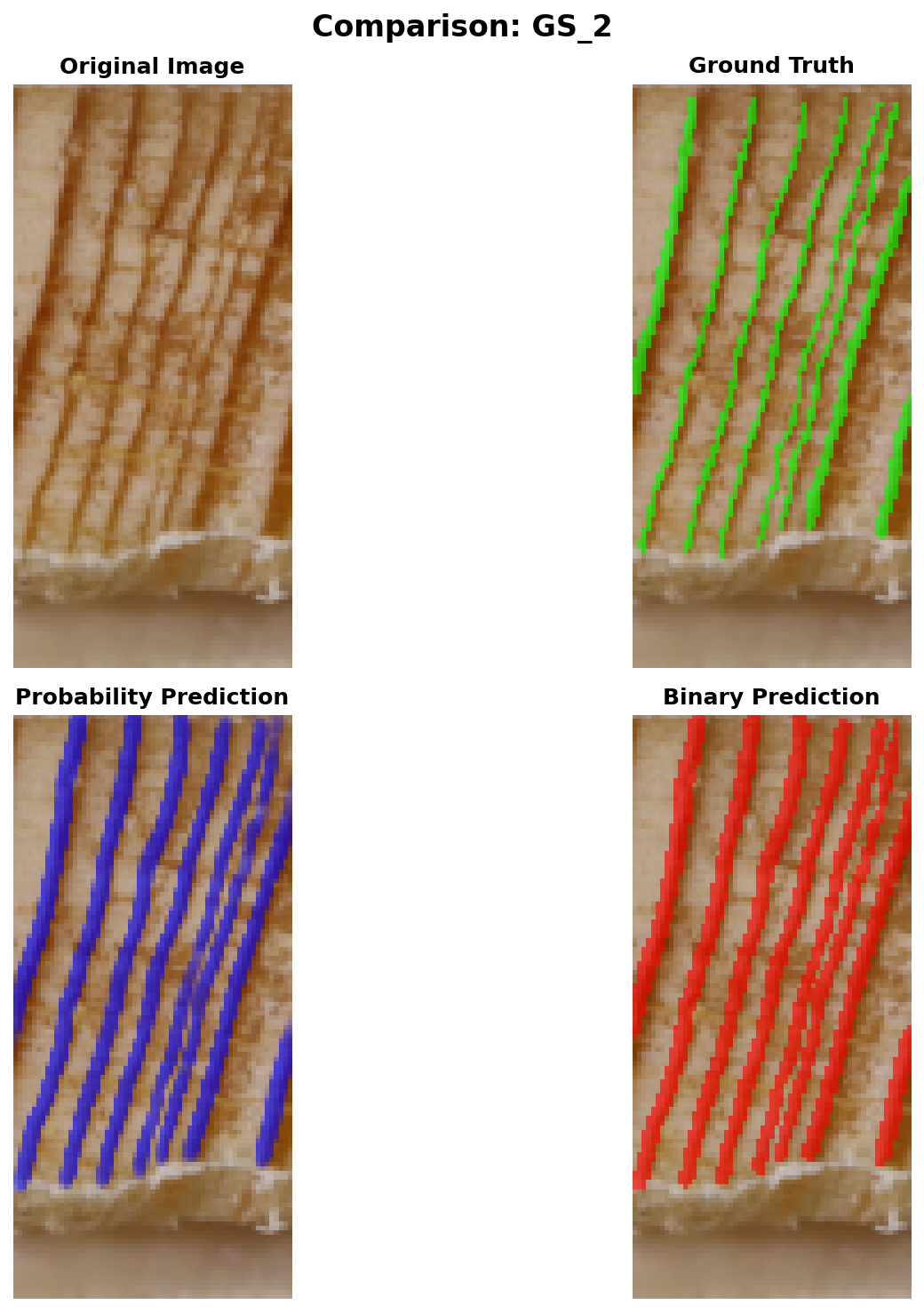

Supplement: S5 Appendix — The supplementary material contains two main folders: -plots: This folder includes, for each image of the GS studied in the focused evaluation dataset, the original image, the labels, the probability prediction (mean value of the 20 trained models), and the binary prediction (results obtained by applying a 0.5 threshold to the probability prediction). -results: This folder contains two files. results_table: A table including the distance measures (measured in µm/10) described in S2_Table. The column “tp_512px” corresponds to the true positives for the 512pxData. A value of 1 indicates a true positive, and a value of 0 means the ring was not correctly detected. histogram_results: A table containing the data used to generate Fig 4b. (ZIP) [file pone.0321841.s013.zip › plots/gs_plots_im_gt_predprob_predbin_128px/GS_2_composite.png]

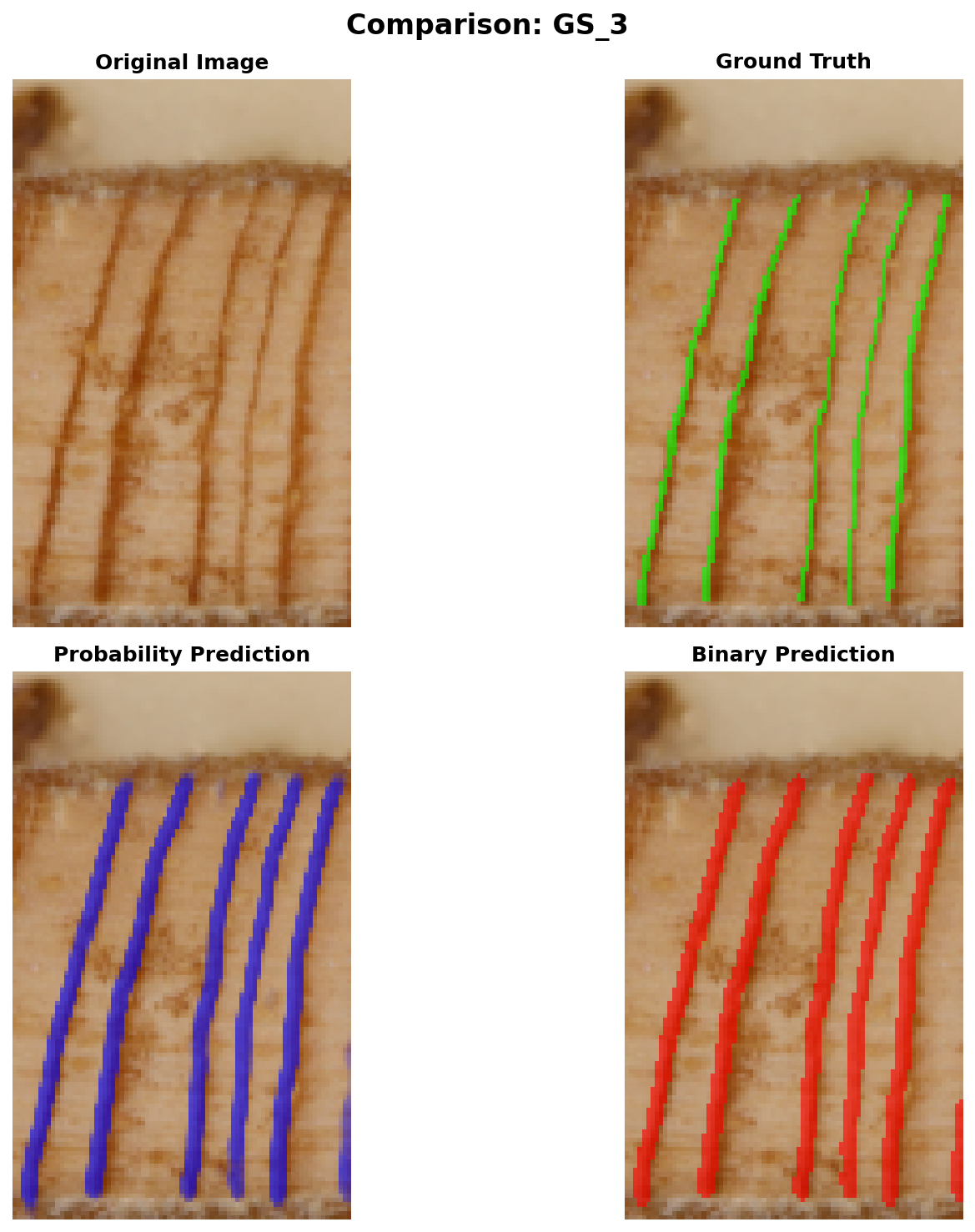

Supplement: S5 Appendix — The supplementary material contains two main folders: -plots: This folder includes, for each image of the GS studied in the focused evaluation dataset, the original image, the labels, the probability prediction (mean value of the 20 trained models), and the binary prediction (results obtained by applying a 0.5 threshold to the probability prediction). -results: This folder contains two files. results_table: A table including the distance measures (measured in µm/10) described in S2_Table. The column “tp_512px” corresponds to the true positives for the 512pxData. A value of 1 indicates a true positive, and a value of 0 means the ring was not correctly detected. histogram_results: A table containing the data used to generate Fig 4b. (ZIP) [file pone.0321841.s013.zip › plots/gs_plots_im_gt_predprob_predbin_128px/GS_3_composite.png]

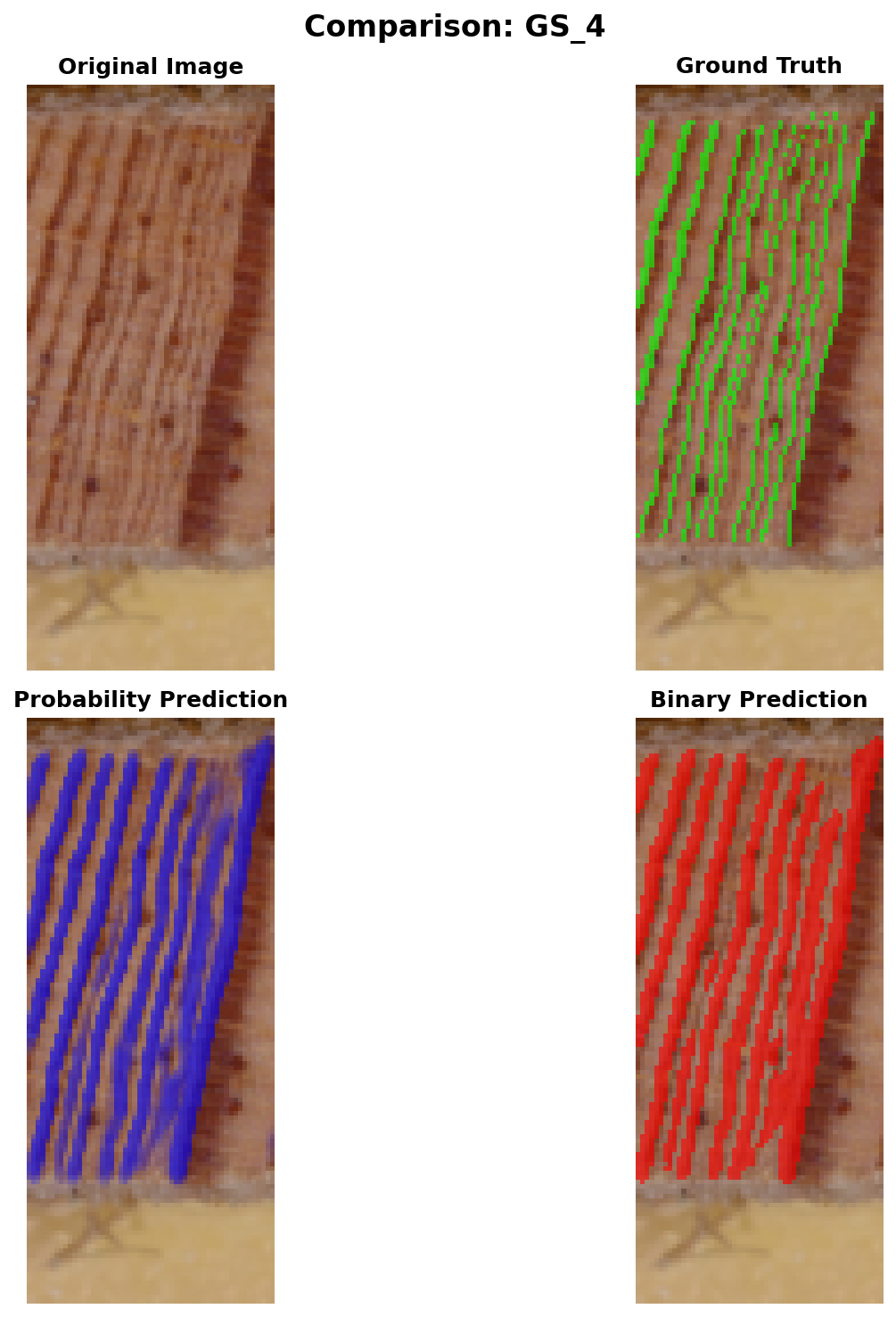

Supplement: S5 Appendix — The supplementary material contains two main folders: -plots: This folder includes, for each image of the GS studied in the focused evaluation dataset, the original image, the labels, the probability prediction (mean value of the 20 trained models), and the binary prediction (results obtained by applying a 0.5 threshold to the probability prediction). -results: This folder contains two files. results_table: A table including the distance measures (measured in µm/10) described in S2_Table. The column “tp_512px” corresponds to the true positives for the 512pxData. A value of 1 indicates a true positive, and a value of 0 means the ring was not correctly detected. histogram_results: A table containing the data used to generate Fig 4b. (ZIP) [file pone.0321841.s013.zip › plots/gs_plots_im_gt_predprob_predbin_128px/GS_4_composite.png]

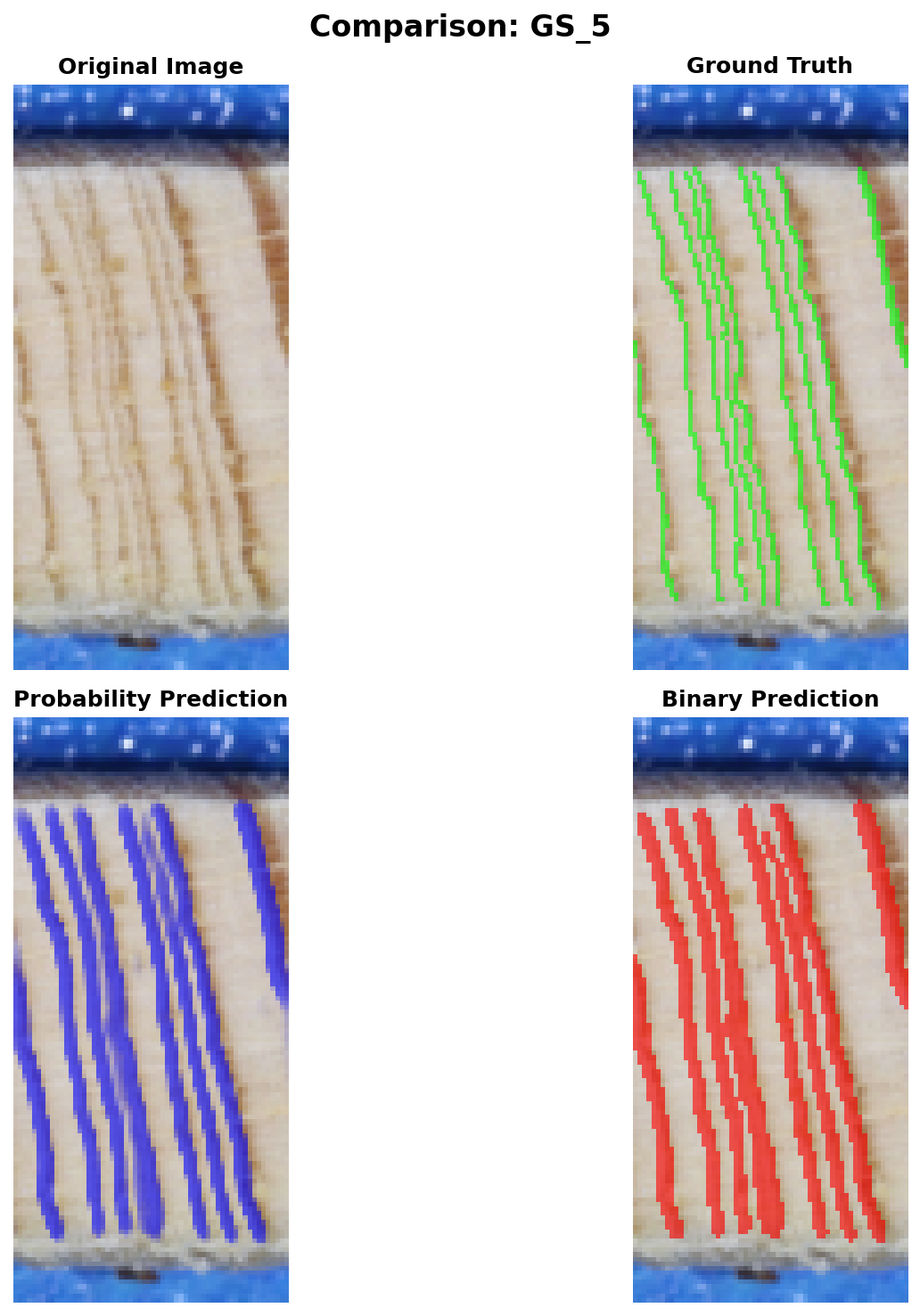

Supplement: S5 Appendix — The supplementary material contains two main folders: -plots: This folder includes, for each image of the GS studied in the focused evaluation dataset, the original image, the labels, the probability prediction (mean value of the 20 trained models), and the binary prediction (results obtained by applying a 0.5 threshold to the probability prediction). -results: This folder contains two files. results_table: A table including the distance measures (measured in µm/10) described in S2_Table. The column “tp_512px” corresponds to the true positives for the 512pxData. A value of 1 indicates a true positive, and a value of 0 means the ring was not correctly detected. histogram_results: A table containing the data used to generate Fig 4b. (ZIP) [file pone.0321841.s013.zip › plots/gs_plots_im_gt_predprob_predbin_128px/GS_5_composite.png]

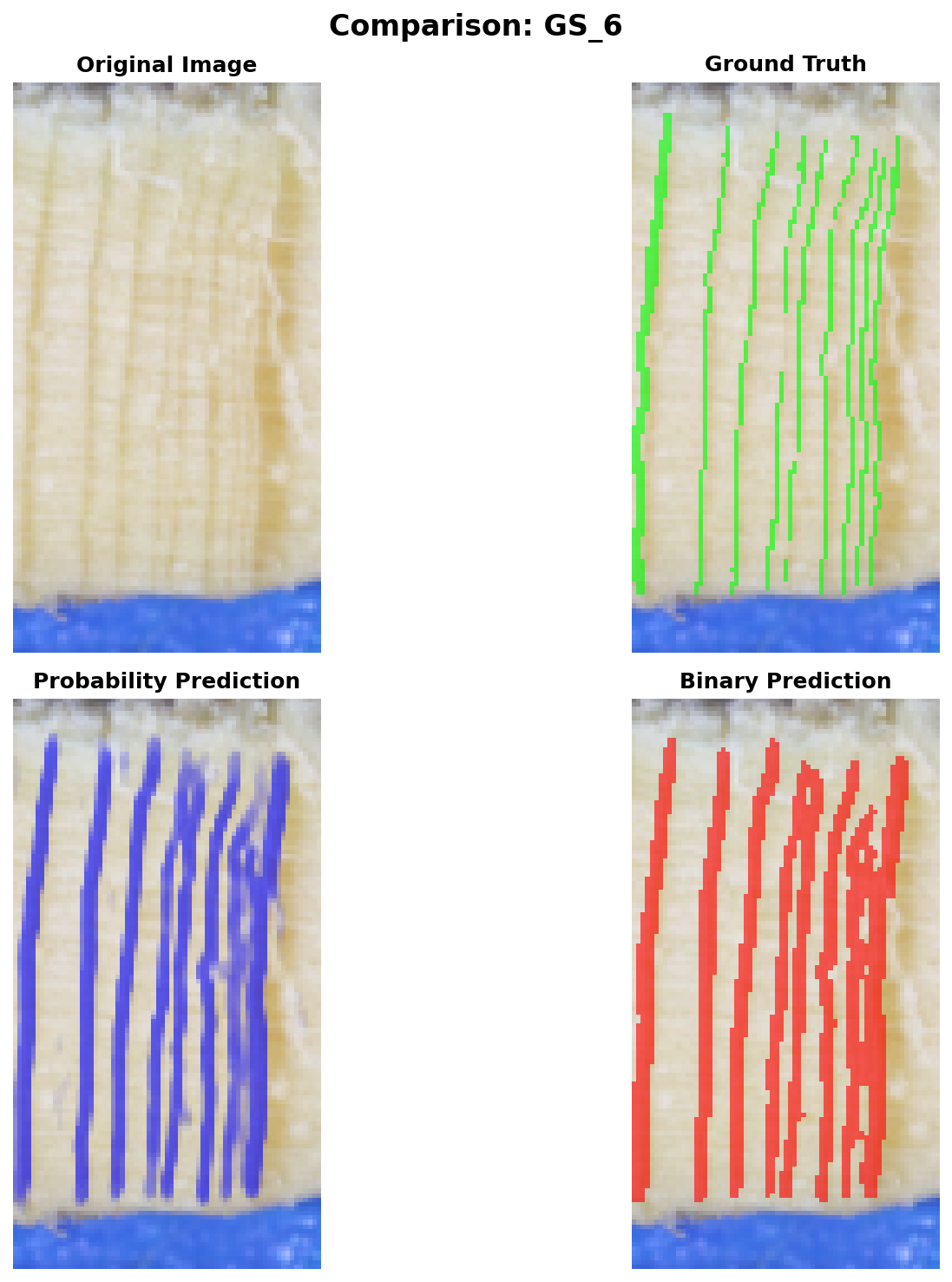

Supplement: S5 Appendix — The supplementary material contains two main folders: -plots: This folder includes, for each image of the GS studied in the focused evaluation dataset, the original image, the labels, the probability prediction (mean value of the 20 trained models), and the binary prediction (results obtained by applying a 0.5 threshold to the probability prediction). -results: This folder contains two files. results_table: A table including the distance measures (measured in µm/10) described in S2_Table. The column “tp_512px” corresponds to the true positives for the 512pxData. A value of 1 indicates a true positive, and a value of 0 means the ring was not correctly detected. histogram_results: A table containing the data used to generate Fig 4b. (ZIP) [file pone.0321841.s013.zip › plots/gs_plots_im_gt_predprob_predbin_128px/GS_6_composite.png]

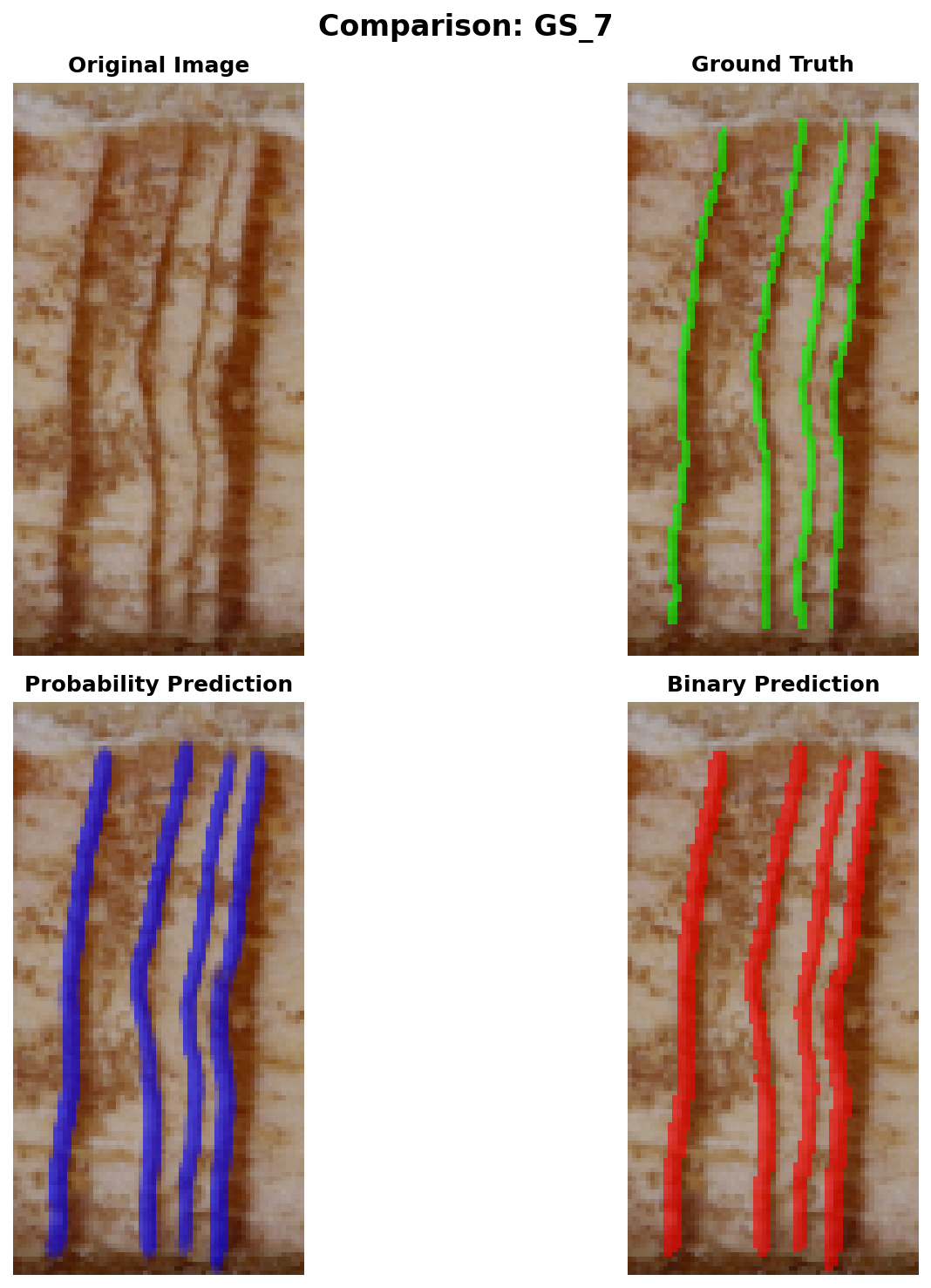

Supplement: S5 Appendix — The supplementary material contains two main folders: -plots: This folder includes, for each image of the GS studied in the focused evaluation dataset, the original image, the labels, the probability prediction (mean value of the 20 trained models), and the binary prediction (results obtained by applying a 0.5 threshold to the probability prediction). -results: This folder contains two files. results_table: A table including the distance measures (measured in µm/10) described in S2_Table. The column “tp_512px” corresponds to the true positives for the 512pxData. A value of 1 indicates a true positive, and a value of 0 means the ring was not correctly detected. histogram_results: A table containing the data used to generate Fig 4b. (ZIP) [file pone.0321841.s013.zip › plots/gs_plots_im_gt_predprob_predbin_128px/GS_7_composite.png]

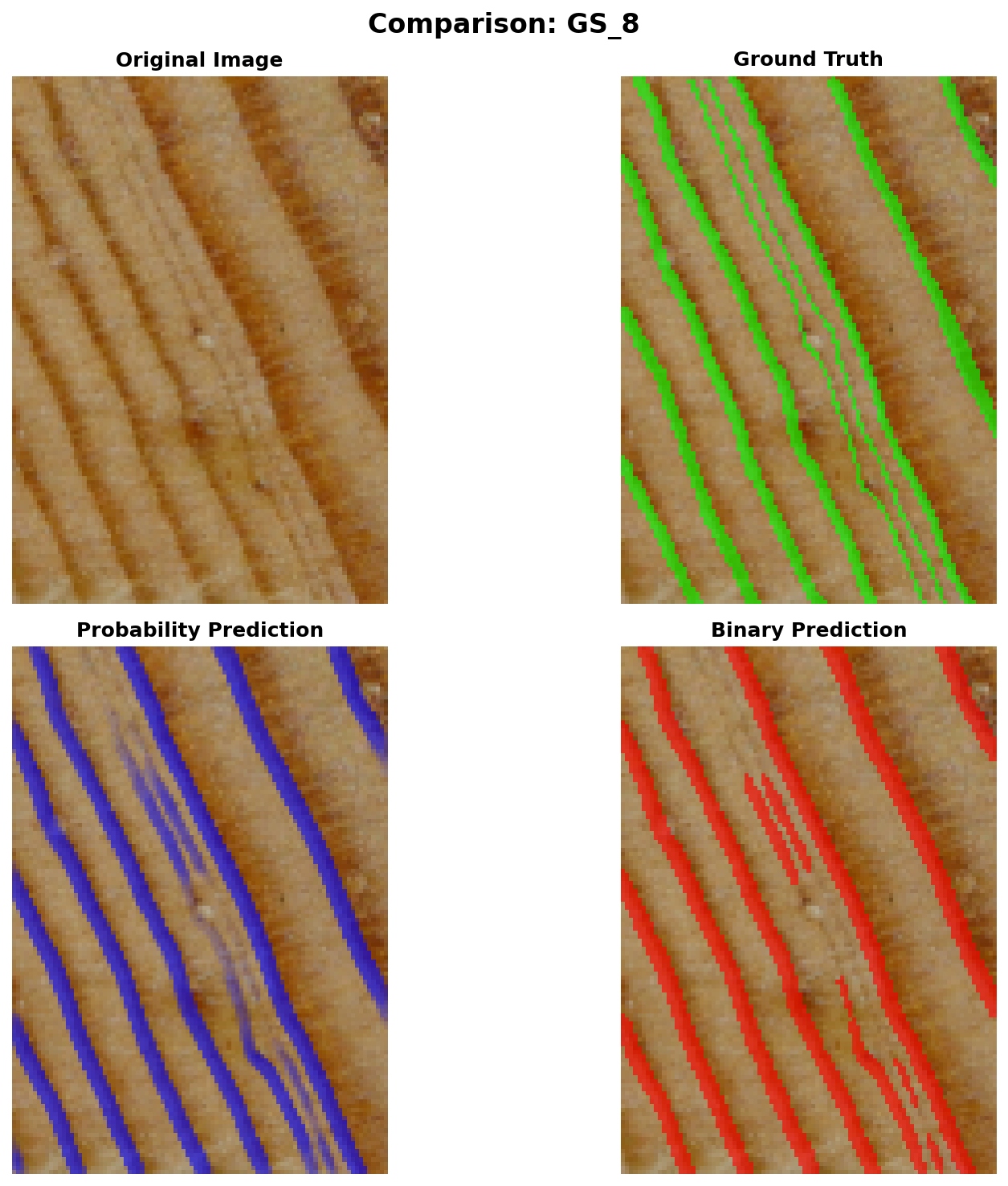

Supplement: S5 Appendix — The supplementary material contains two main folders: -plots: This folder includes, for each image of the GS studied in the focused evaluation dataset, the original image, the labels, the probability prediction (mean value of the 20 trained models), and the binary prediction (results obtained by applying a 0.5 threshold to the probability prediction). -results: This folder contains two files. results_table: A table including the distance measures (measured in µm/10) described in S2_Table. The column “tp_512px” corresponds to the true positives for the 512pxData. A value of 1 indicates a true positive, and a value of 0 means the ring was not correctly detected. histogram_results: A table containing the data used to generate Fig 4b. (ZIP) [file pone.0321841.s013.zip › plots/gs_plots_im_gt_predprob_predbin_128px/GS_8_composite.png]

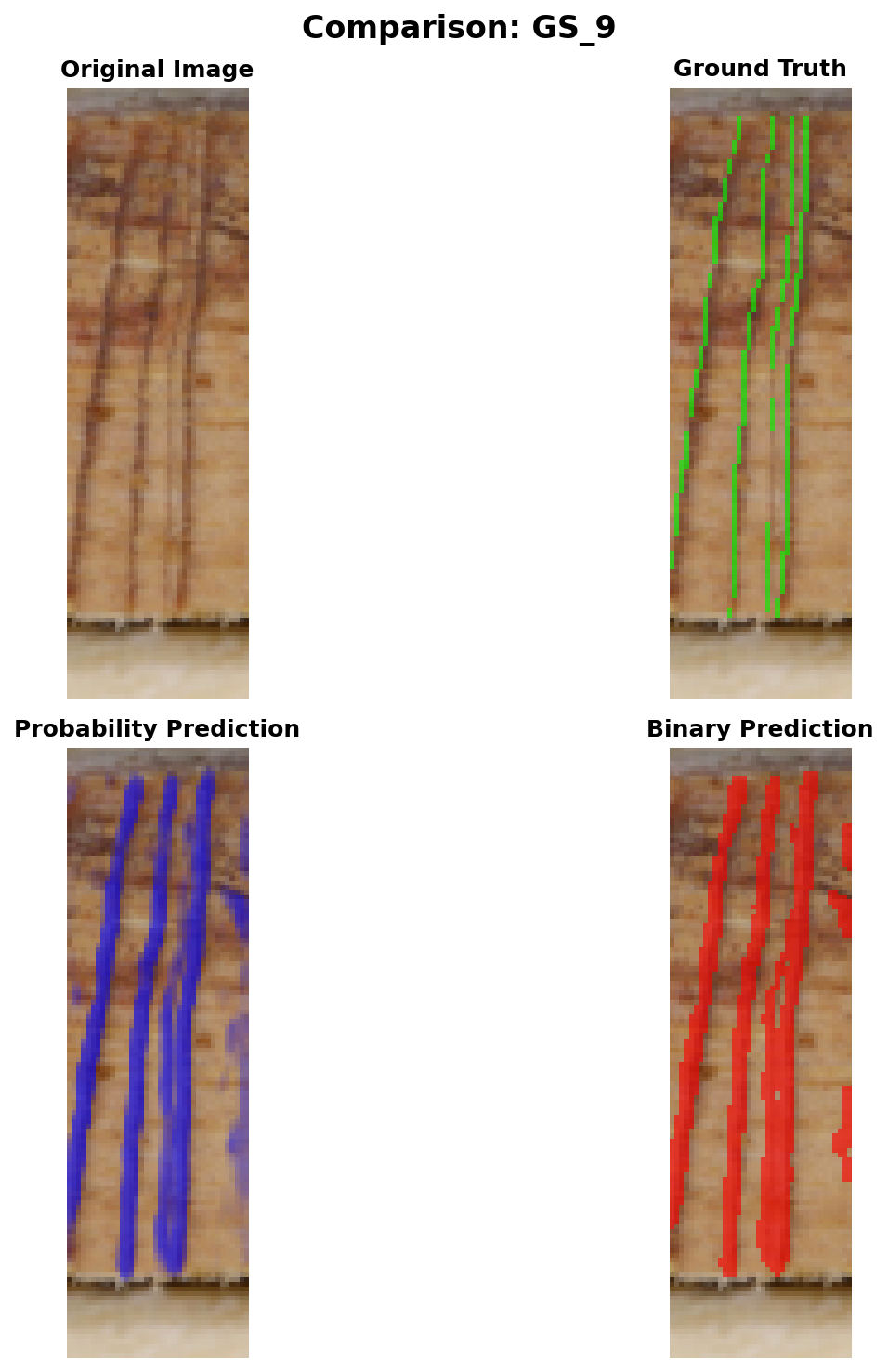

Supplement: S5 Appendix — The supplementary material contains two main folders: -plots: This folder includes, for each image of the GS studied in the focused evaluation dataset, the original image, the labels, the probability prediction (mean value of the 20 trained models), and the binary prediction (results obtained by applying a 0.5 threshold to the probability prediction). -results: This folder contains two files. results_table: A table including the distance measures (measured in µm/10) described in S2_Table. The column “tp_512px” corresponds to the true positives for the 512pxData. A value of 1 indicates a true positive, and a value of 0 means the ring was not correctly detected. histogram_results: A table containing the data used to generate Fig 4b. (ZIP) [file pone.0321841.s013.zip › plots/gs_plots_im_gt_predprob_predbin_128px/GS_9_composite.png]

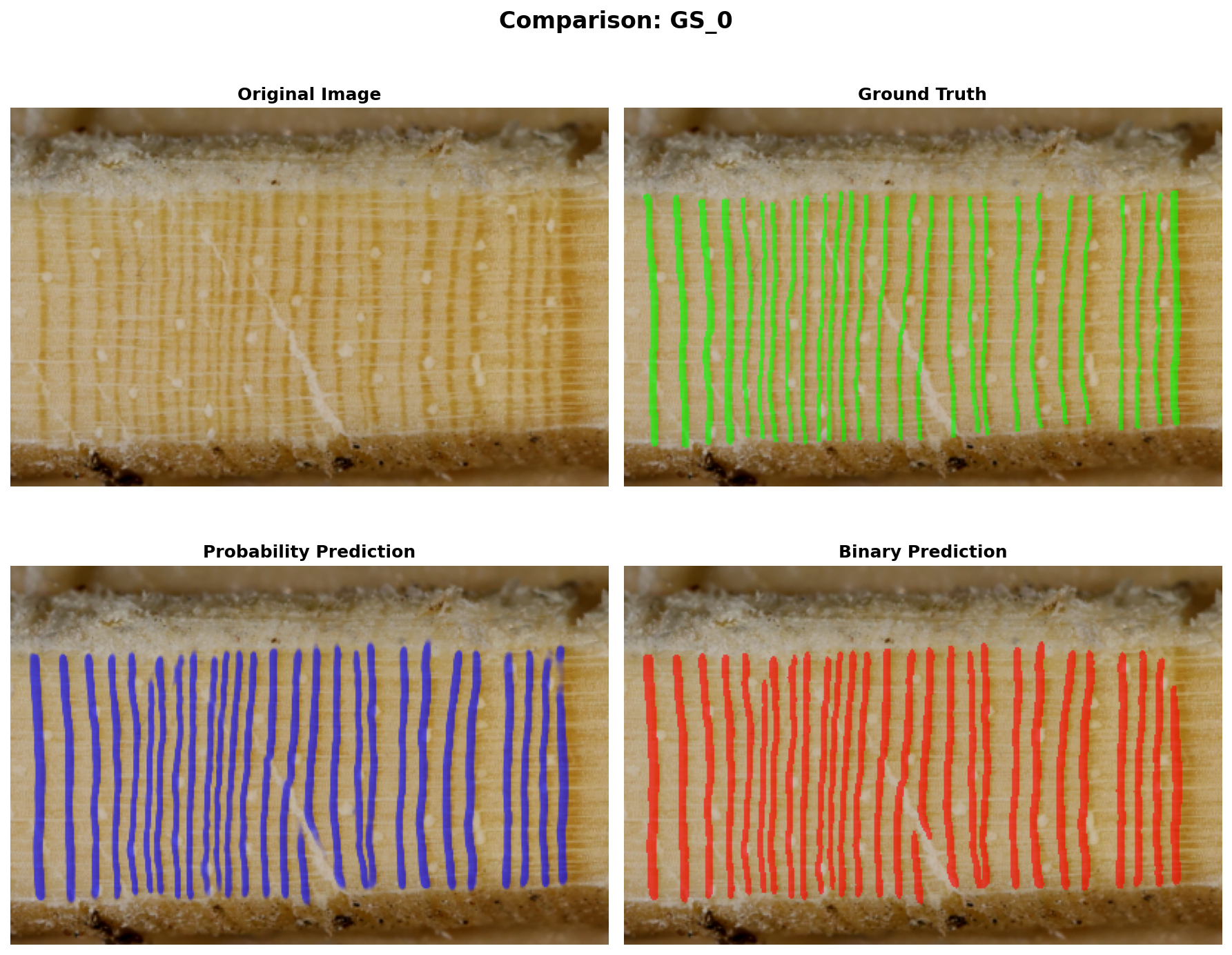

Supplement: S5 Appendix — The supplementary material contains two main folders: -plots: This folder includes, for each image of the GS studied in the focused evaluation dataset, the original image, the labels, the probability prediction (mean value of the 20 trained models), and the binary prediction (results obtained by applying a 0.5 threshold to the probability prediction). -results: This folder contains two files. results_table: A table including the distance measures (measured in µm/10) described in S2_Table. The column “tp_512px” corresponds to the true positives for the 512pxData. A value of 1 indicates a true positive, and a value of 0 means the ring was not correctly detected. histogram_results: A table containing the data used to generate Fig 4b. (ZIP) [file pone.0321841.s013.zip › plots/gs_plots_im_gt_predprob_predbin_256px/GS_0_composite.png]

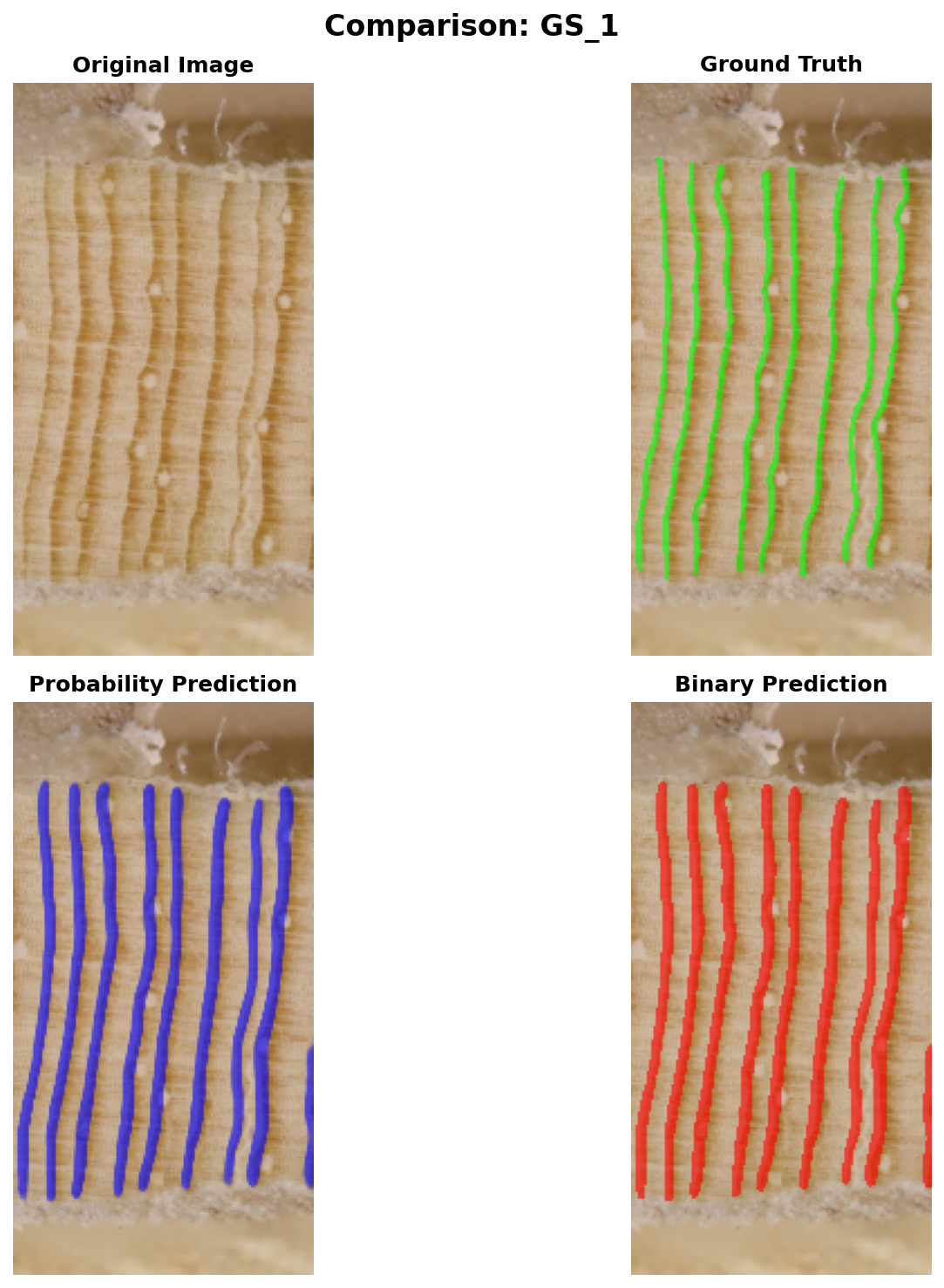

Supplement: S5 Appendix — The supplementary material contains two main folders: -plots: This folder includes, for each image of the GS studied in the focused evaluation dataset, the original image, the labels, the probability prediction (mean value of the 20 trained models), and the binary prediction (results obtained by applying a 0.5 threshold to the probability prediction). -results: This folder contains two files. results_table: A table including the distance measures (measured in µm/10) described in S2_Table. The column “tp_512px” corresponds to the true positives for the 512pxData. A value of 1 indicates a true positive, and a value of 0 means the ring was not correctly detected. histogram_results: A table containing the data used to generate Fig 4b. (ZIP) [file pone.0321841.s013.zip › plots/gs_plots_im_gt_predprob_predbin_256px/GS_1_composite.png]

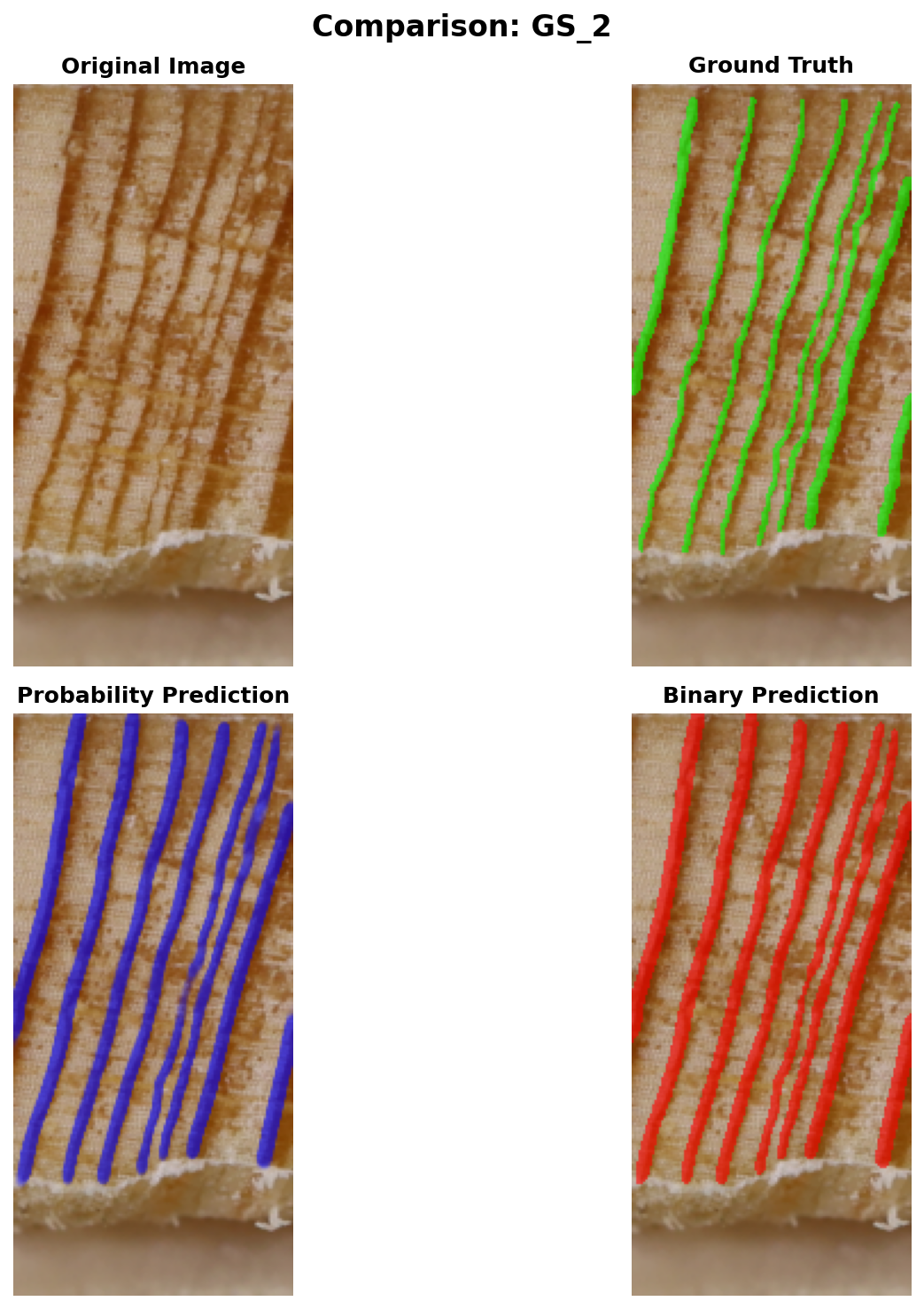

Supplement: S5 Appendix — The supplementary material contains two main folders: -plots: This folder includes, for each image of the GS studied in the focused evaluation dataset, the original image, the labels, the probability prediction (mean value of the 20 trained models), and the binary prediction (results obtained by applying a 0.5 threshold to the probability prediction). -results: This folder contains two files. results_table: A table including the distance measures (measured in µm/10) described in S2_Table. The column “tp_512px” corresponds to the true positives for the 512pxData. A value of 1 indicates a true positive, and a value of 0 means the ring was not correctly detected. histogram_results: A table containing the data used to generate Fig 4b. (ZIP) [file pone.0321841.s013.zip › plots/gs_plots_im_gt_predprob_predbin_256px/GS_2_composite.png]

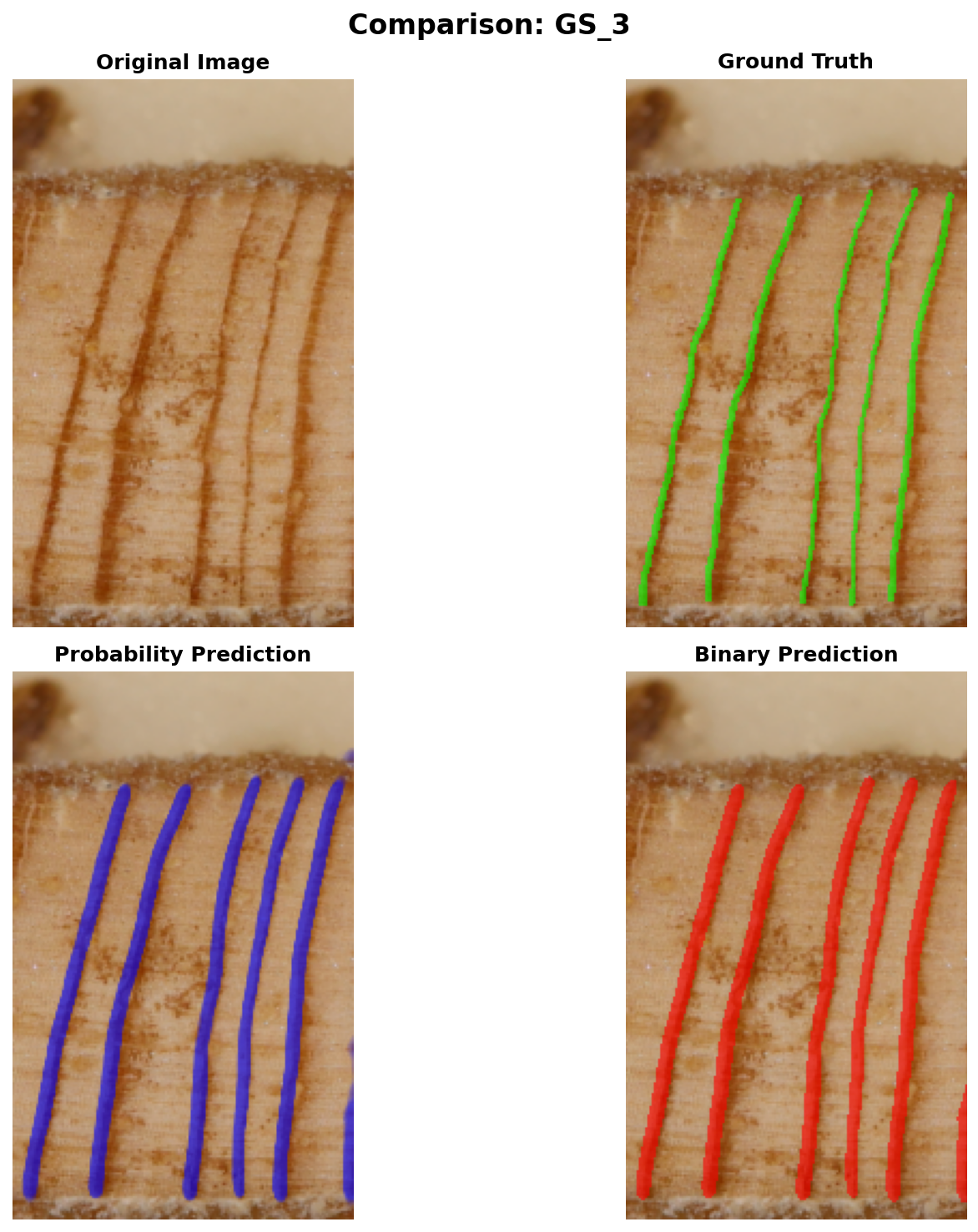

Supplement: S5 Appendix — The supplementary material contains two main folders: -plots: This folder includes, for each image of the GS studied in the focused evaluation dataset, the original image, the labels, the probability prediction (mean value of the 20 trained models), and the binary prediction (results obtained by applying a 0.5 threshold to the probability prediction). -results: This folder contains two files. results_table: A table including the distance measures (measured in µm/10) described in S2_Table. The column “tp_512px” corresponds to the true positives for the 512pxData. A value of 1 indicates a true positive, and a value of 0 means the ring was not correctly detected. histogram_results: A table containing the data used to generate Fig 4b. (ZIP) [file pone.0321841.s013.zip › plots/gs_plots_im_gt_predprob_predbin_256px/GS_3_composite.png]

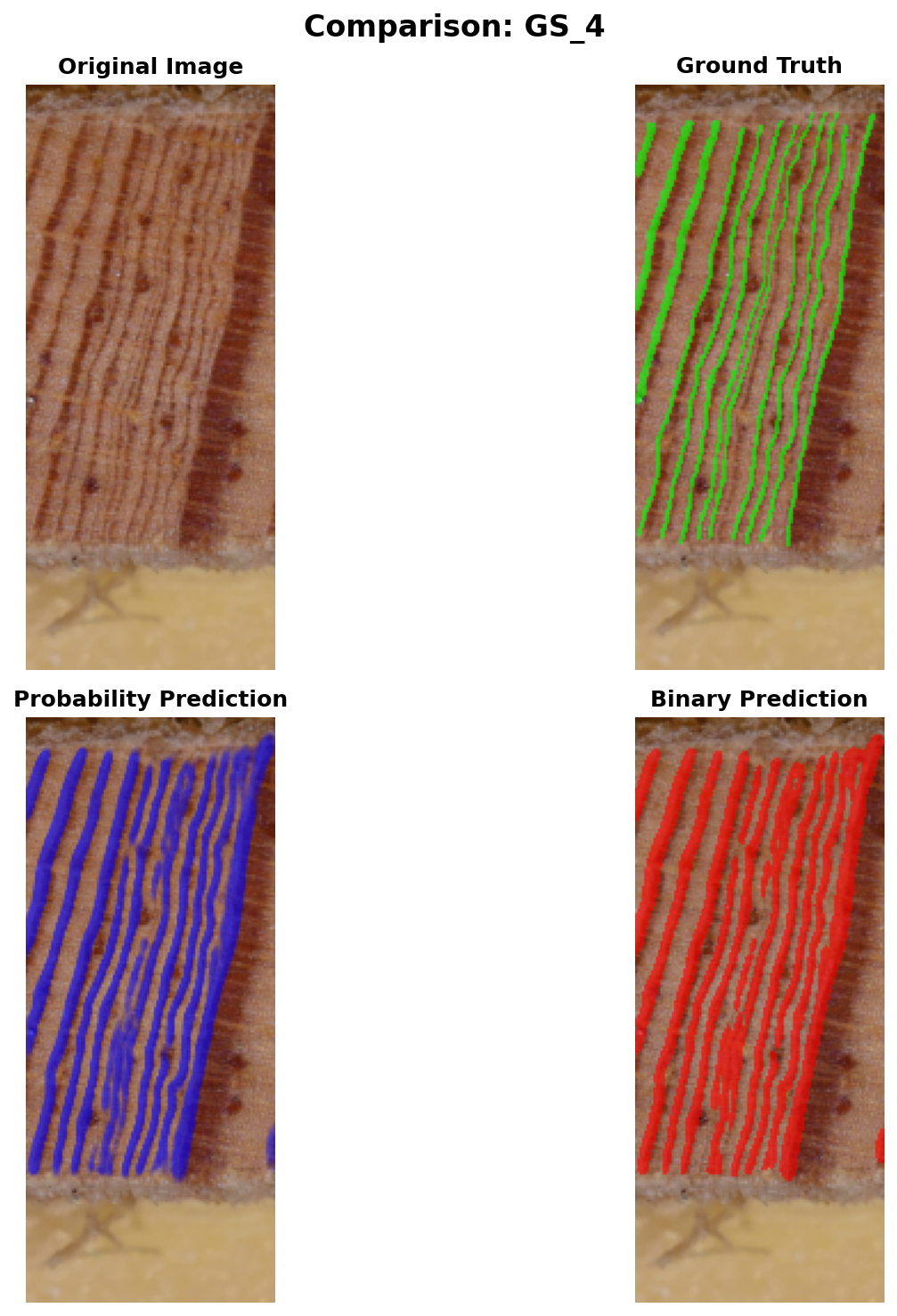

Supplement: S5 Appendix — The supplementary material contains two main folders: -plots: This folder includes, for each image of the GS studied in the focused evaluation dataset, the original image, the labels, the probability prediction (mean value of the 20 trained models), and the binary prediction (results obtained by applying a 0.5 threshold to the probability prediction). -results: This folder contains two files. results_table: A table including the distance measures (measured in µm/10) described in S2_Table. The column “tp_512px” corresponds to the true positives for the 512pxData. A value of 1 indicates a true positive, and a value of 0 means the ring was not correctly detected. histogram_results: A table containing the data used to generate Fig 4b. (ZIP) [file pone.0321841.s013.zip › plots/gs_plots_im_gt_predprob_predbin_256px/GS_4_composite.png]

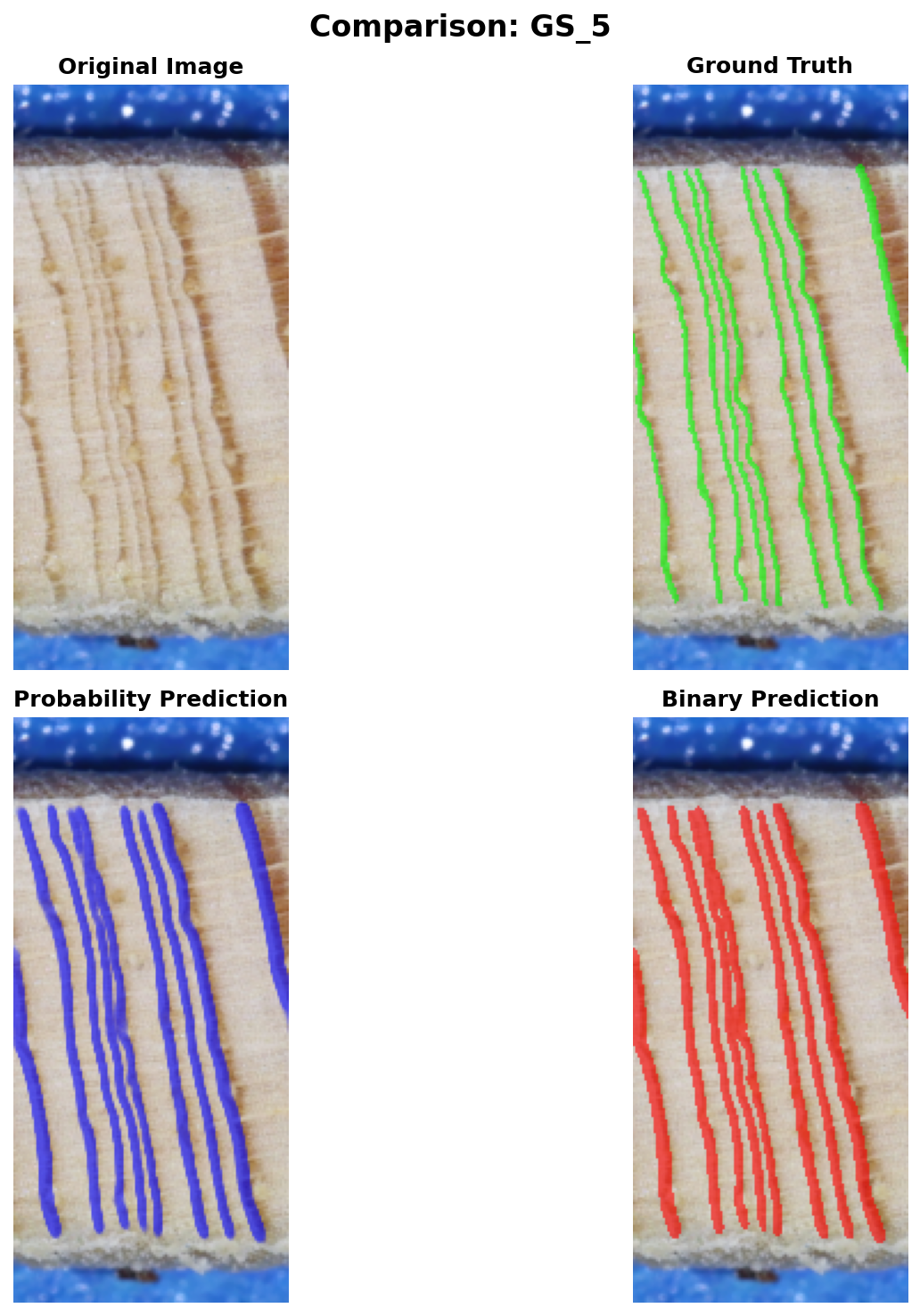

Supplement: S5 Appendix — The supplementary material contains two main folders: -plots: This folder includes, for each image of the GS studied in the focused evaluation dataset, the original image, the labels, the probability prediction (mean value of the 20 trained models), and the binary prediction (results obtained by applying a 0.5 threshold to the probability prediction). -results: This folder contains two files. results_table: A table including the distance measures (measured in µm/10) described in S2_Table. The column “tp_512px” corresponds to the true positives for the 512pxData. A value of 1 indicates a true positive, and a value of 0 means the ring was not correctly detected. histogram_results: A table containing the data used to generate Fig 4b. (ZIP) [file pone.0321841.s013.zip › plots/gs_plots_im_gt_predprob_predbin_256px/GS_5_composite.png]

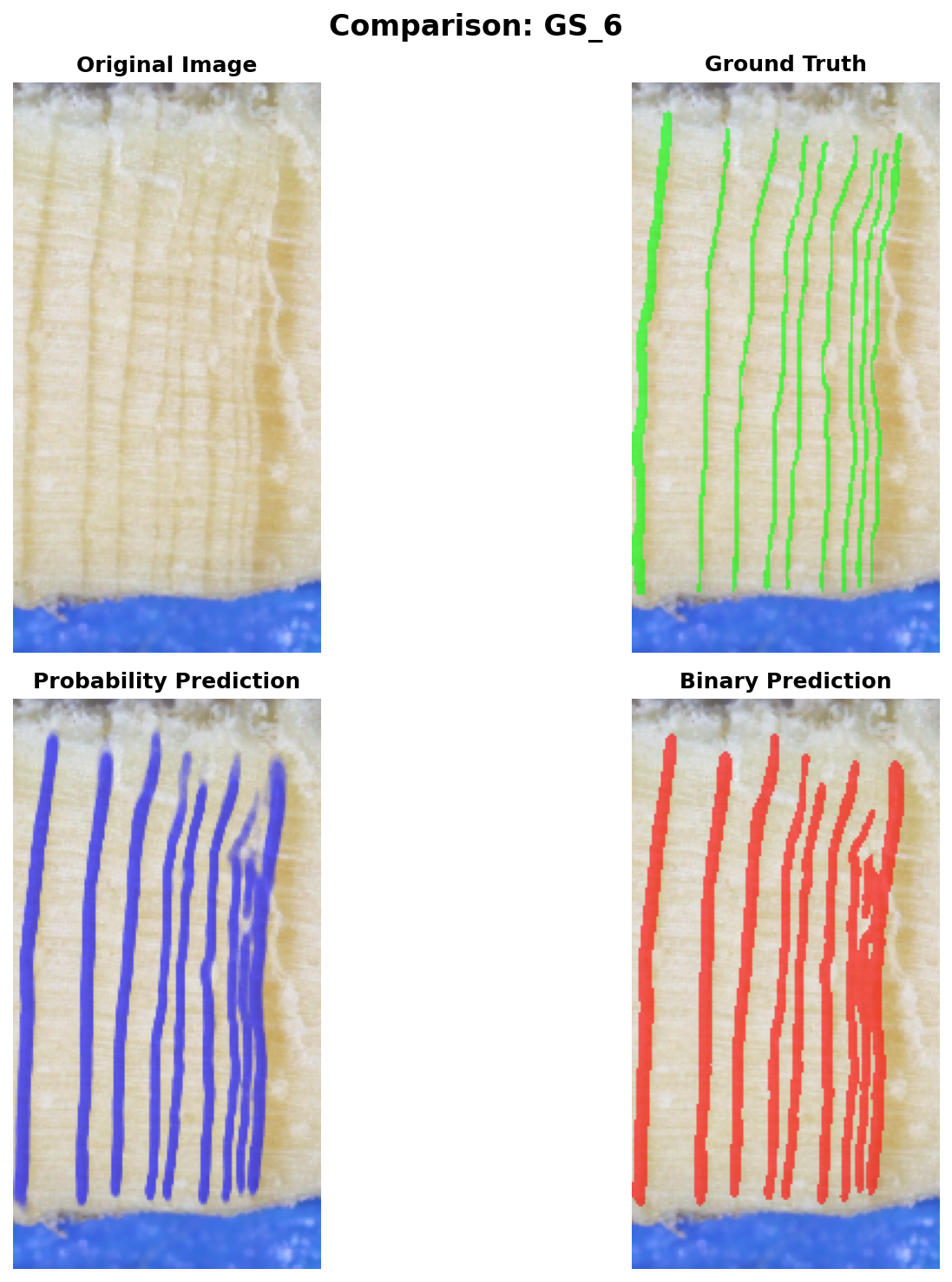

Supplement: S5 Appendix — The supplementary material contains two main folders: -plots: This folder includes, for each image of the GS studied in the focused evaluation dataset, the original image, the labels, the probability prediction (mean value of the 20 trained models), and the binary prediction (results obtained by applying a 0.5 threshold to the probability prediction). -results: This folder contains two files. results_table: A table including the distance measures (measured in µm/10) described in S2_Table. The column “tp_512px” corresponds to the true positives for the 512pxData. A value of 1 indicates a true positive, and a value of 0 means the ring was not correctly detected. histogram_results: A table containing the data used to generate Fig 4b. (ZIP) [file pone.0321841.s013.zip › plots/gs_plots_im_gt_predprob_predbin_256px/GS_6_composite.png]

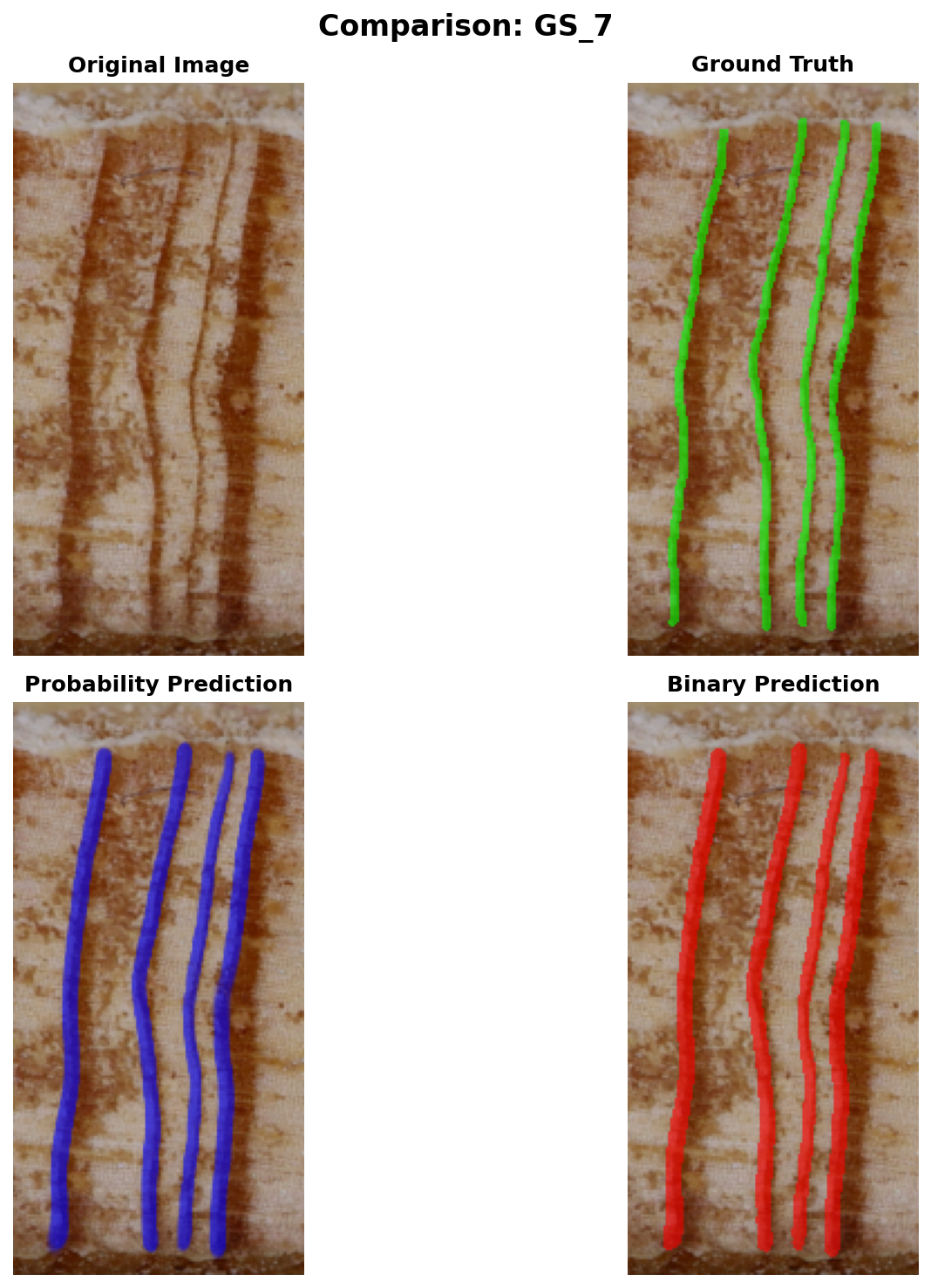

Supplement: S5 Appendix — The supplementary material contains two main folders: -plots: This folder includes, for each image of the GS studied in the focused evaluation dataset, the original image, the labels, the probability prediction (mean value of the 20 trained models), and the binary prediction (results obtained by applying a 0.5 threshold to the probability prediction). -results: This folder contains two files. results_table: A table including the distance measures (measured in µm/10) described in S2_Table. The column “tp_512px” corresponds to the true positives for the 512pxData. A value of 1 indicates a true positive, and a value of 0 means the ring was not correctly detected. histogram_results: A table containing the data used to generate Fig 4b. (ZIP) [file pone.0321841.s013.zip › plots/gs_plots_im_gt_predprob_predbin_256px/GS_7_composite.png]

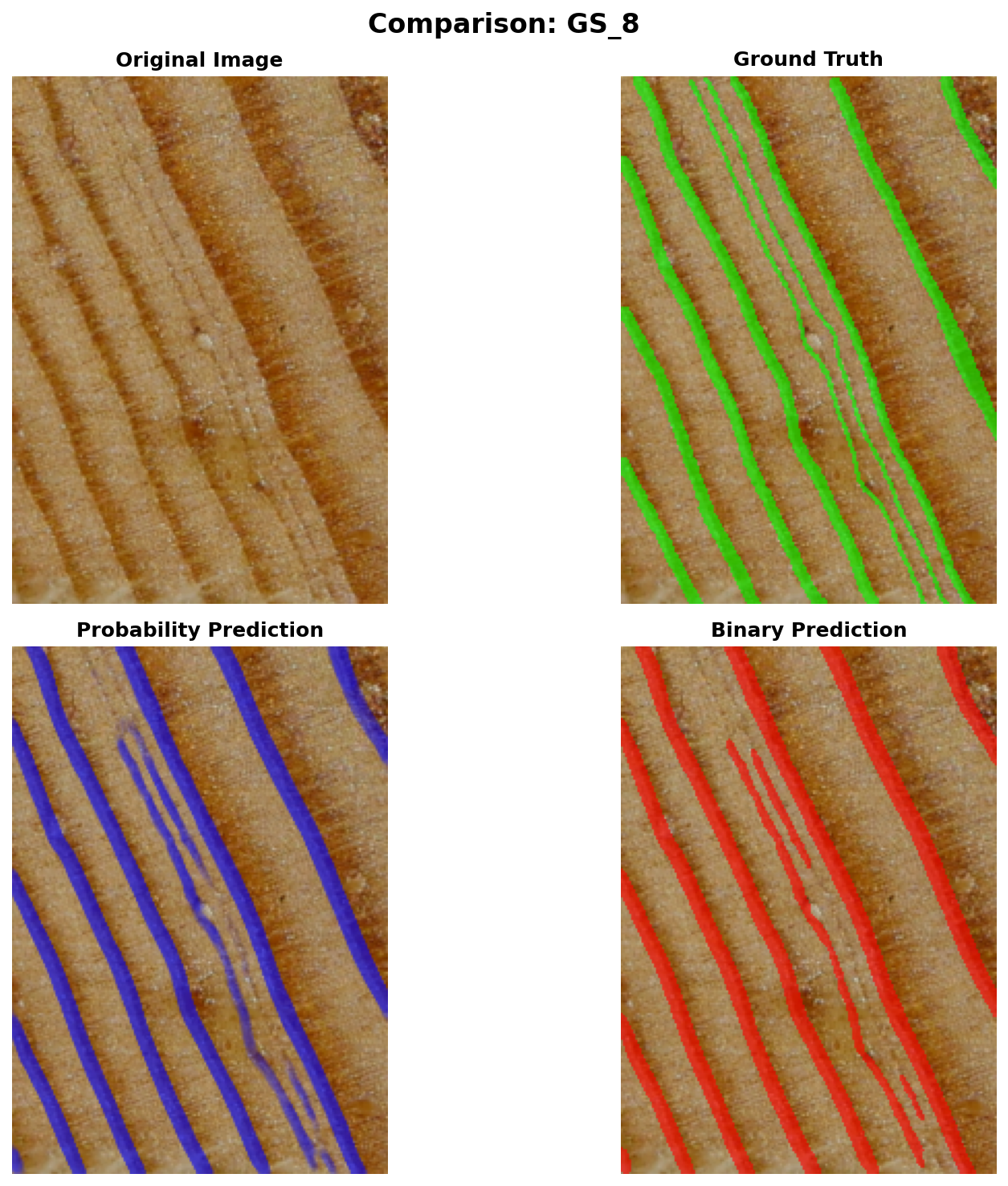

Supplement: S5 Appendix — The supplementary material contains two main folders: -plots: This folder includes, for each image of the GS studied in the focused evaluation dataset, the original image, the labels, the probability prediction (mean value of the 20 trained models), and the binary prediction (results obtained by applying a 0.5 threshold to the probability prediction). -results: This folder contains two files. results_table: A table including the distance measures (measured in µm/10) described in S2_Table. The column “tp_512px” corresponds to the true positives for the 512pxData. A value of 1 indicates a true positive, and a value of 0 means the ring was not correctly detected. histogram_results: A table containing the data used to generate Fig 4b. (ZIP) [file pone.0321841.s013.zip › plots/gs_plots_im_gt_predprob_predbin_256px/GS_8_composite.png]

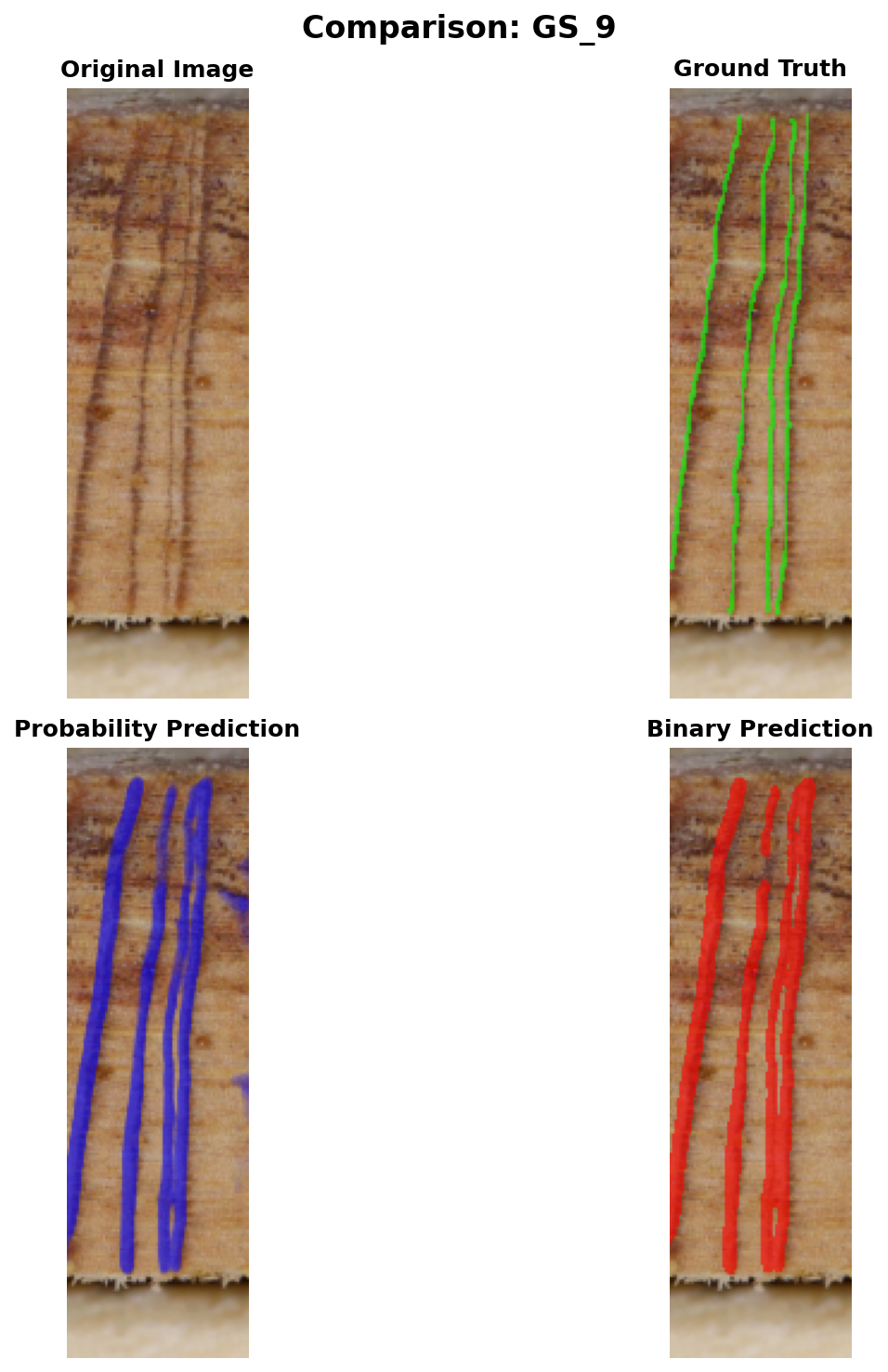

Supplement: S5 Appendix — The supplementary material contains two main folders: -plots: This folder includes, for each image of the GS studied in the focused evaluation dataset, the original image, the labels, the probability prediction (mean value of the 20 trained models), and the binary prediction (results obtained by applying a 0.5 threshold to the probability prediction). -results: This folder contains two files. results_table: A table including the distance measures (measured in µm/10) described in S2_Table. The column “tp_512px” corresponds to the true positives for the 512pxData. A value of 1 indicates a true positive, and a value of 0 means the ring was not correctly detected. histogram_results: A table containing the data used to generate Fig 4b. (ZIP) [file pone.0321841.s013.zip › plots/gs_plots_im_gt_predprob_predbin_256px/GS_9_composite.png]

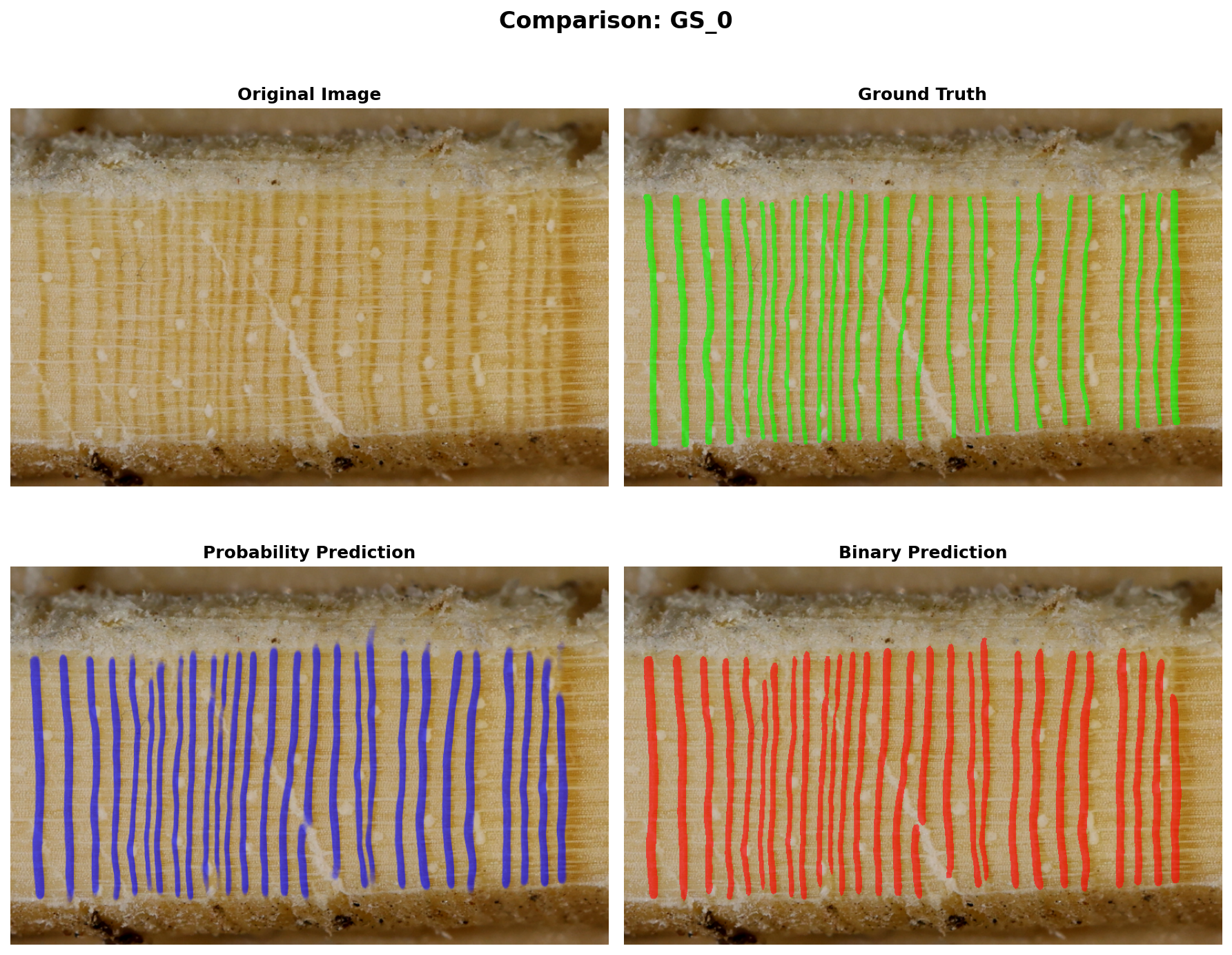

Supplement: S5 Appendix — The supplementary material contains two main folders: -plots: This folder includes, for each image of the GS studied in the focused evaluation dataset, the original image, the labels, the probability prediction (mean value of the 20 trained models), and the binary prediction (results obtained by applying a 0.5 threshold to the probability prediction). -results: This folder contains two files. results_table: A table including the distance measures (measured in µm/10) described in S2_Table. The column “tp_512px” corresponds to the true positives for the 512pxData. A value of 1 indicates a true positive, and a value of 0 means the ring was not correctly detected. histogram_results: A table containing the data used to generate Fig 4b. (ZIP) [file pone.0321841.s013.zip › plots/gs_plots_im_gt_predprob_predbin_512px/GS_0_composite.png]

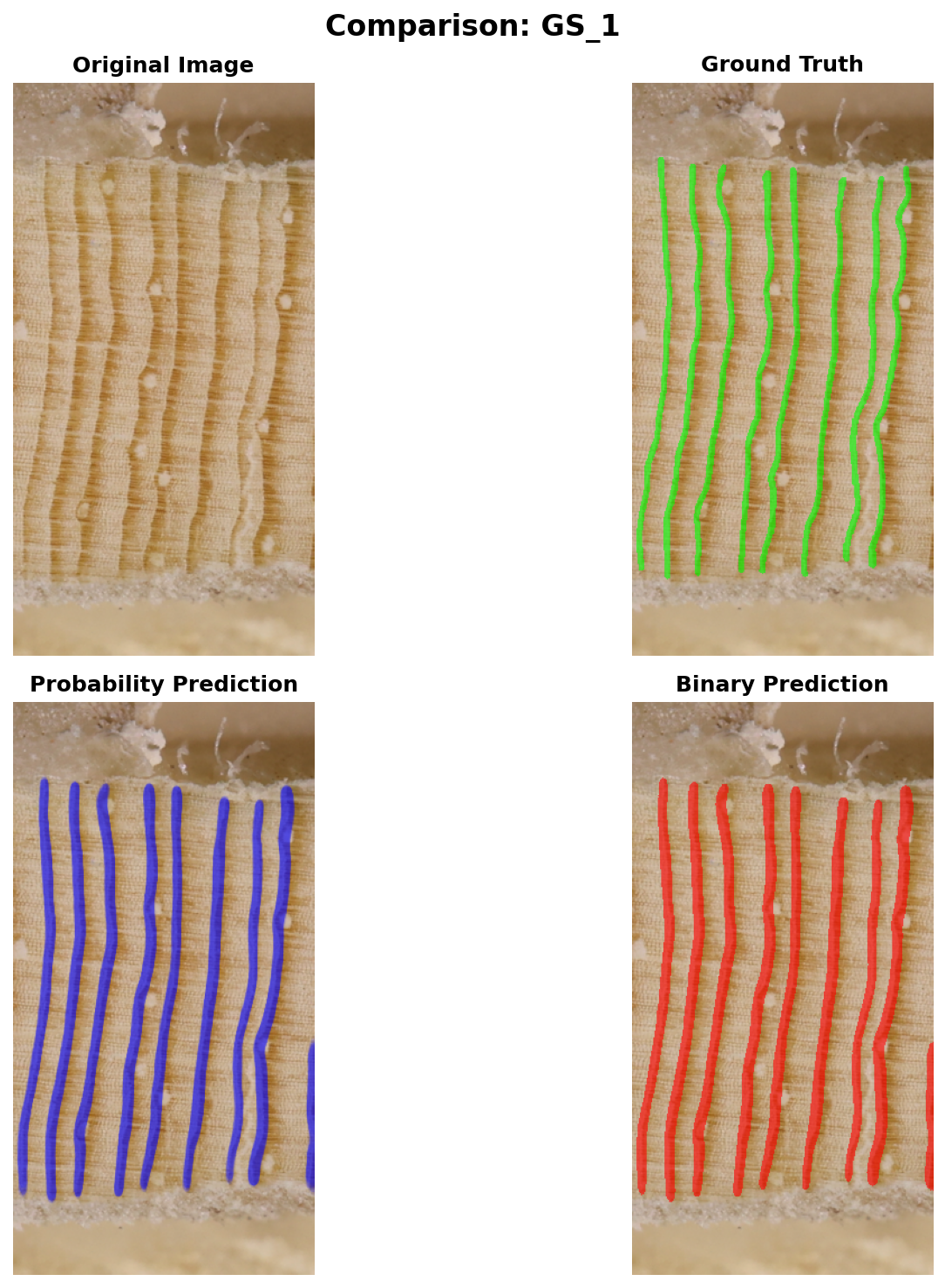

Supplement: S5 Appendix — The supplementary material contains two main folders: -plots: This folder includes, for each image of the GS studied in the focused evaluation dataset, the original image, the labels, the probability prediction (mean value of the 20 trained models), and the binary prediction (results obtained by applying a 0.5 threshold to the probability prediction). -results: This folder contains two files. results_table: A table including the distance measures (measured in µm/10) described in S2_Table. The column “tp_512px” corresponds to the true positives for the 512pxData. A value of 1 indicates a true positive, and a value of 0 means the ring was not correctly detected. histogram_results: A table containing the data used to generate Fig 4b. (ZIP) [file pone.0321841.s013.zip › plots/gs_plots_im_gt_predprob_predbin_512px/GS_1_composite.png]

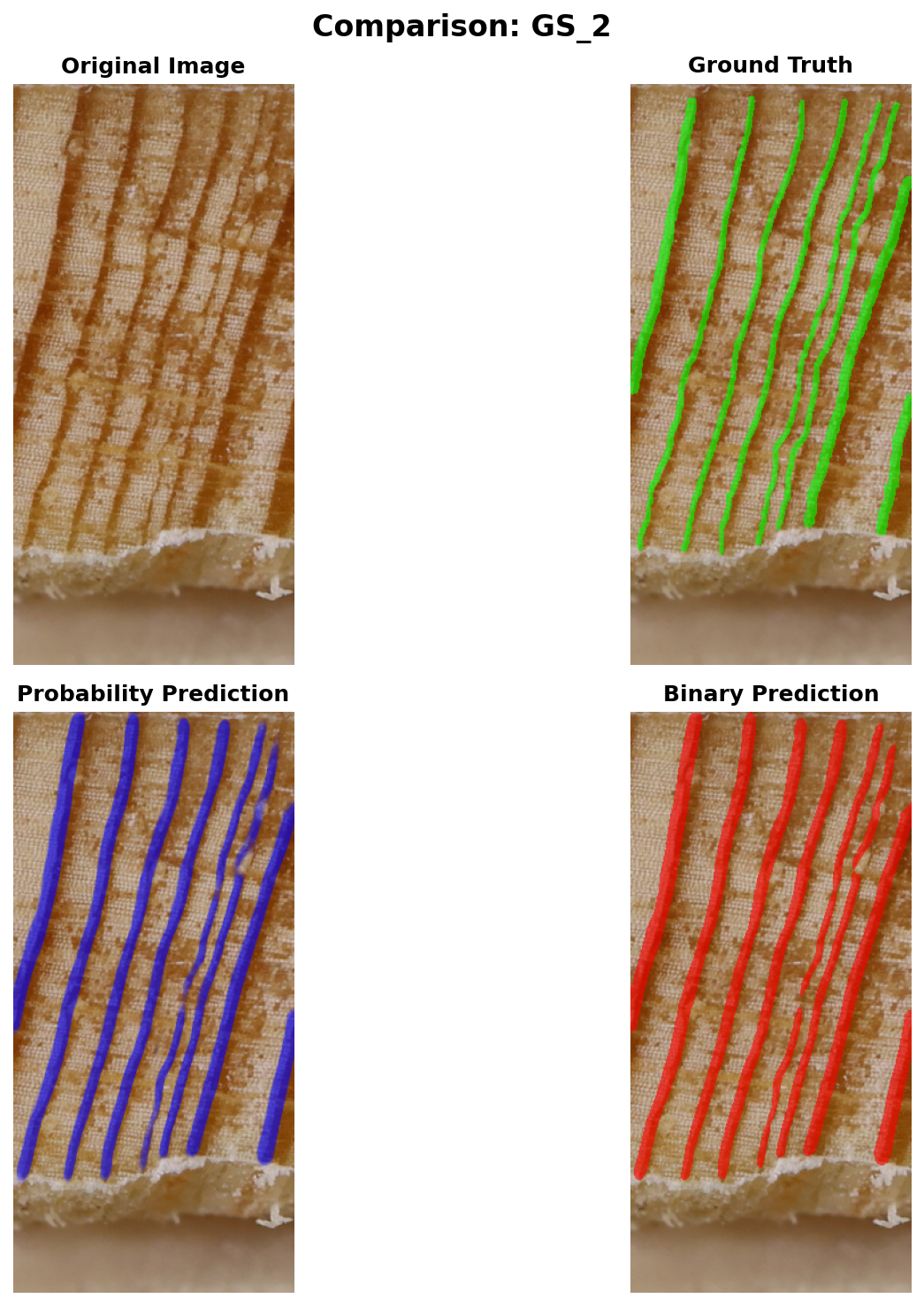

Supplement: S5 Appendix — The supplementary material contains two main folders: -plots: This folder includes, for each image of the GS studied in the focused evaluation dataset, the original image, the labels, the probability prediction (mean value of the 20 trained models), and the binary prediction (results obtained by applying a 0.5 threshold to the probability prediction). -results: This folder contains two files. results_table: A table including the distance measures (measured in µm/10) described in S2_Table. The column “tp_512px” corresponds to the true positives for the 512pxData. A value of 1 indicates a true positive, and a value of 0 means the ring was not correctly detected. histogram_results: A table containing the data used to generate Fig 4b. (ZIP) [file pone.0321841.s013.zip › plots/gs_plots_im_gt_predprob_predbin_512px/GS_2_composite.png]

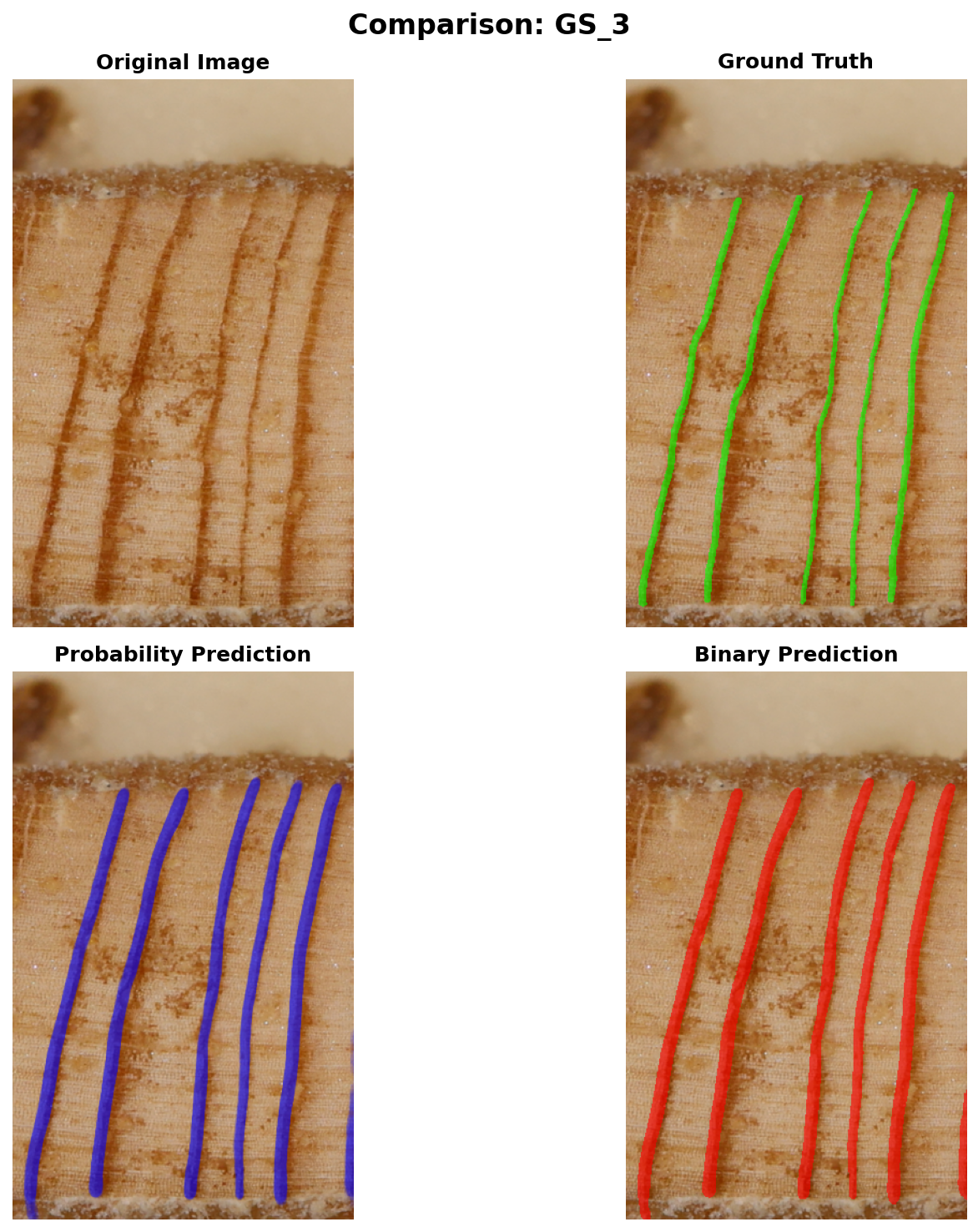

Supplement: S5 Appendix — The supplementary material contains two main folders: -plots: This folder includes, for each image of the GS studied in the focused evaluation dataset, the original image, the labels, the probability prediction (mean value of the 20 trained models), and the binary prediction (results obtained by applying a 0.5 threshold to the probability prediction). -results: This folder contains two files. results_table: A table including the distance measures (measured in µm/10) described in S2_Table. The column “tp_512px” corresponds to the true positives for the 512pxData. A value of 1 indicates a true positive, and a value of 0 means the ring was not correctly detected. histogram_results: A table containing the data used to generate Fig 4b. (ZIP) [file pone.0321841.s013.zip › plots/gs_plots_im_gt_predprob_predbin_512px/GS_3_composite.png]

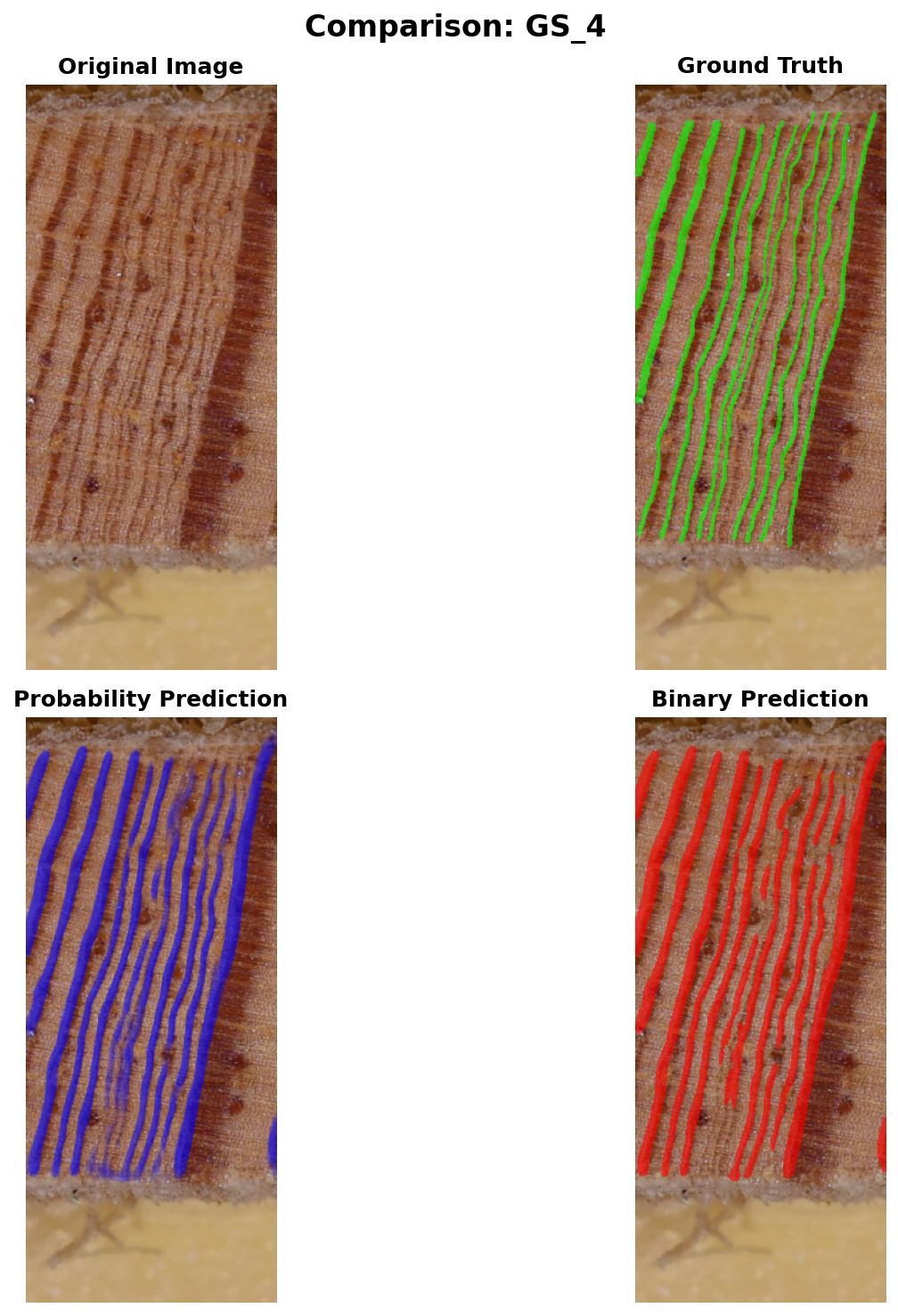

Supplement: S5 Appendix — The supplementary material contains two main folders: -plots: This folder includes, for each image of the GS studied in the focused evaluation dataset, the original image, the labels, the probability prediction (mean value of the 20 trained models), and the binary prediction (results obtained by applying a 0.5 threshold to the probability prediction). -results: This folder contains two files. results_table: A table including the distance measures (measured in µm/10) described in S2_Table. The column “tp_512px” corresponds to the true positives for the 512pxData. A value of 1 indicates a true positive, and a value of 0 means the ring was not correctly detected. histogram_results: A table containing the data used to generate Fig 4b. (ZIP) [file pone.0321841.s013.zip › plots/gs_plots_im_gt_predprob_predbin_512px/GS_4_composite.png]

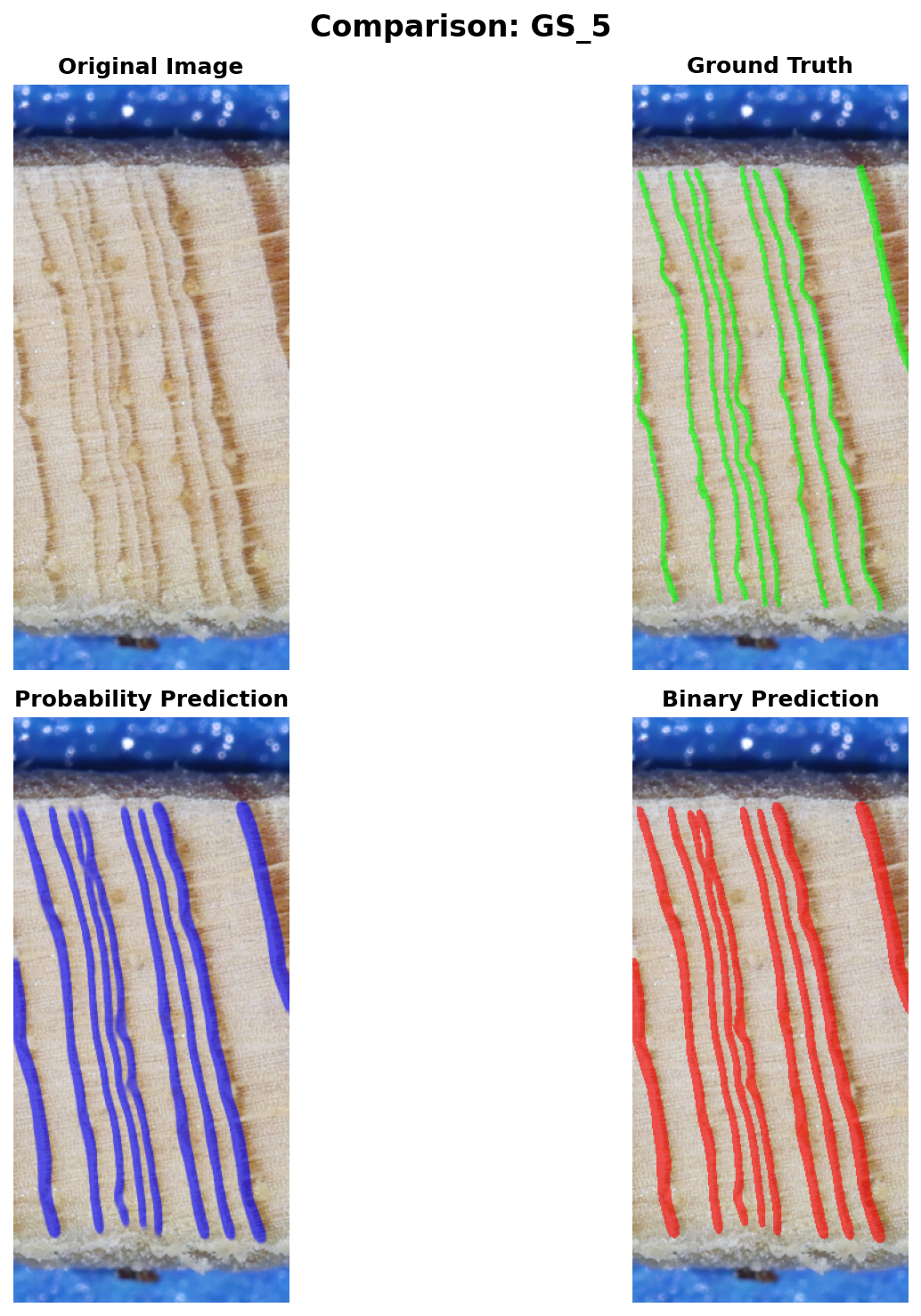

Supplement: S5 Appendix — The supplementary material contains two main folders: -plots: This folder includes, for each image of the GS studied in the focused evaluation dataset, the original image, the labels, the probability prediction (mean value of the 20 trained models), and the binary prediction (results obtained by applying a 0.5 threshold to the probability prediction). -results: This folder contains two files. results_table: A table including the distance measures (measured in µm/10) described in S2_Table. The column “tp_512px” corresponds to the true positives for the 512pxData. A value of 1 indicates a true positive, and a value of 0 means the ring was not correctly detected. histogram_results: A table containing the data used to generate Fig 4b. (ZIP) [file pone.0321841.s013.zip › plots/gs_plots_im_gt_predprob_predbin_512px/GS_5_composite.png]

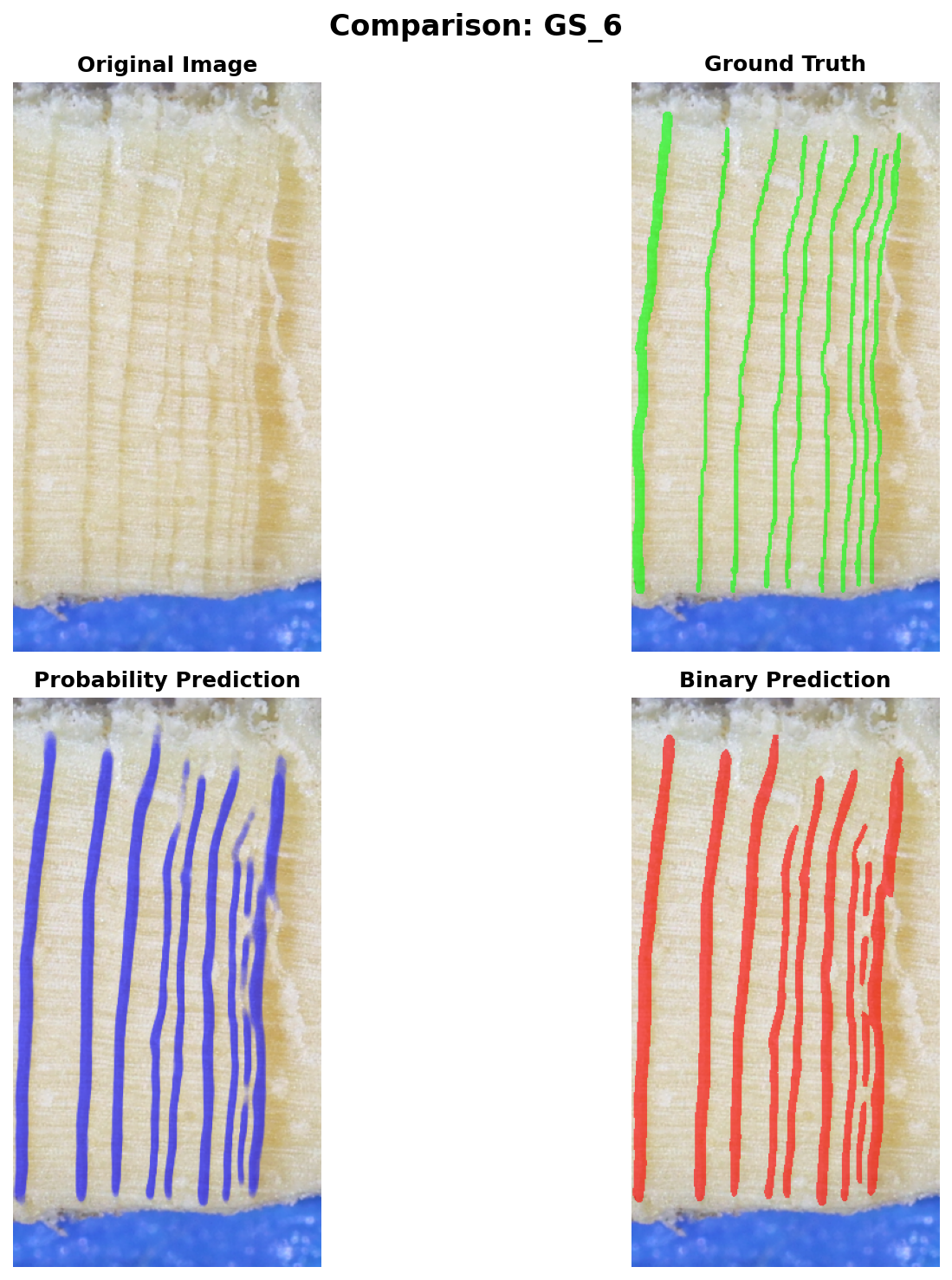

Supplement: S5 Appendix — The supplementary material contains two main folders: -plots: This folder includes, for each image of the GS studied in the focused evaluation dataset, the original image, the labels, the probability prediction (mean value of the 20 trained models), and the binary prediction (results obtained by applying a 0.5 threshold to the probability prediction). -results: This folder contains two files. results_table: A table including the distance measures (measured in µm/10) described in S2_Table. The column “tp_512px” corresponds to the true positives for the 512pxData. A value of 1 indicates a true positive, and a value of 0 means the ring was not correctly detected. histogram_results: A table containing the data used to generate Fig 4b. (ZIP) [file pone.0321841.s013.zip › plots/gs_plots_im_gt_predprob_predbin_512px/GS_6_composite.png]

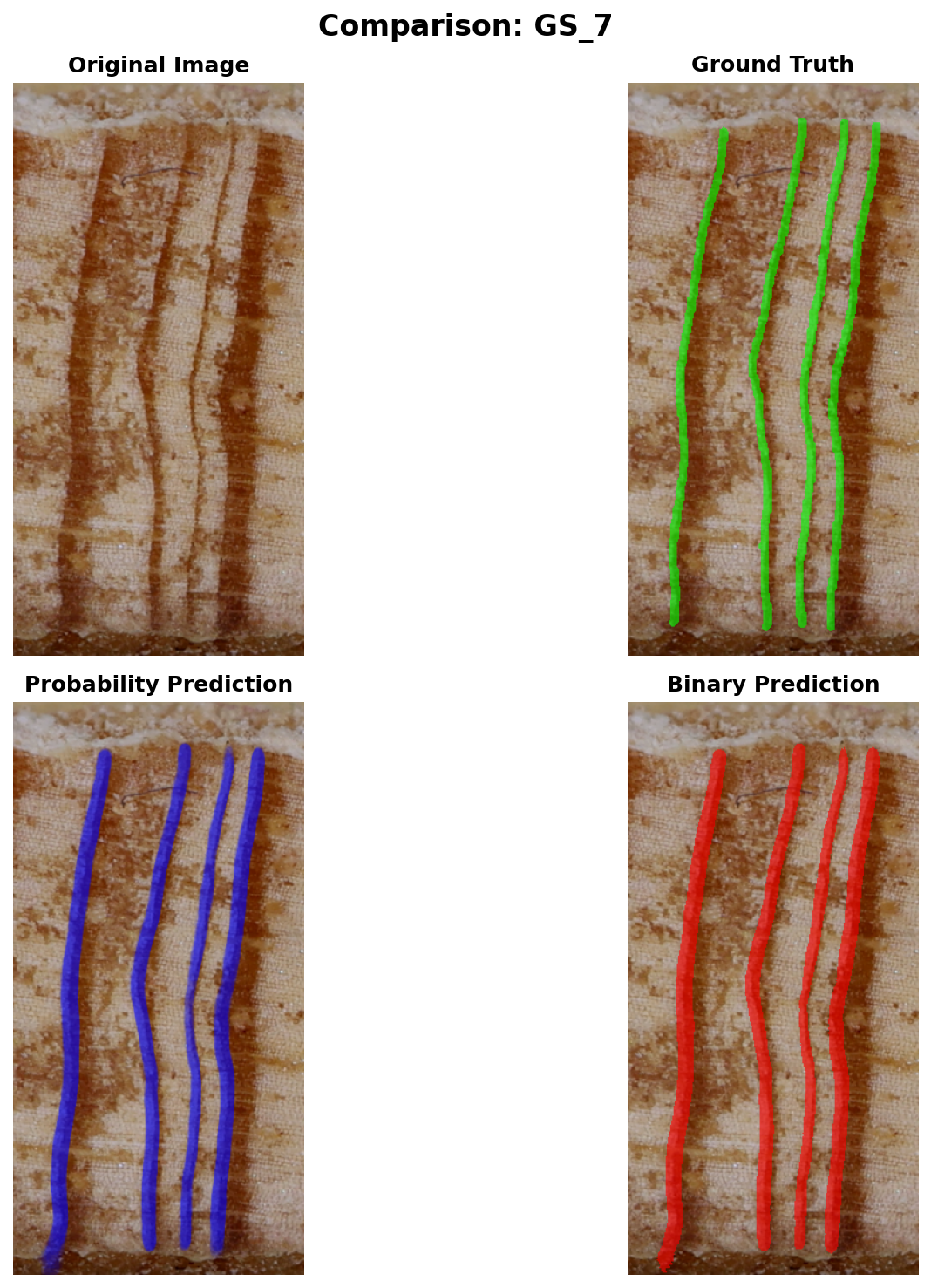

Supplement: S5 Appendix — The supplementary material contains two main folders: -plots: This folder includes, for each image of the GS studied in the focused evaluation dataset, the original image, the labels, the probability prediction (mean value of the 20 trained models), and the binary prediction (results obtained by applying a 0.5 threshold to the probability prediction). -results: This folder contains two files. results_table: A table including the distance measures (measured in µm/10) described in S2_Table. The column “tp_512px” corresponds to the true positives for the 512pxData. A value of 1 indicates a true positive, and a value of 0 means the ring was not correctly detected. histogram_results: A table containing the data used to generate Fig 4b. (ZIP) [file pone.0321841.s013.zip › plots/gs_plots_im_gt_predprob_predbin_512px/GS_7_composite.png]

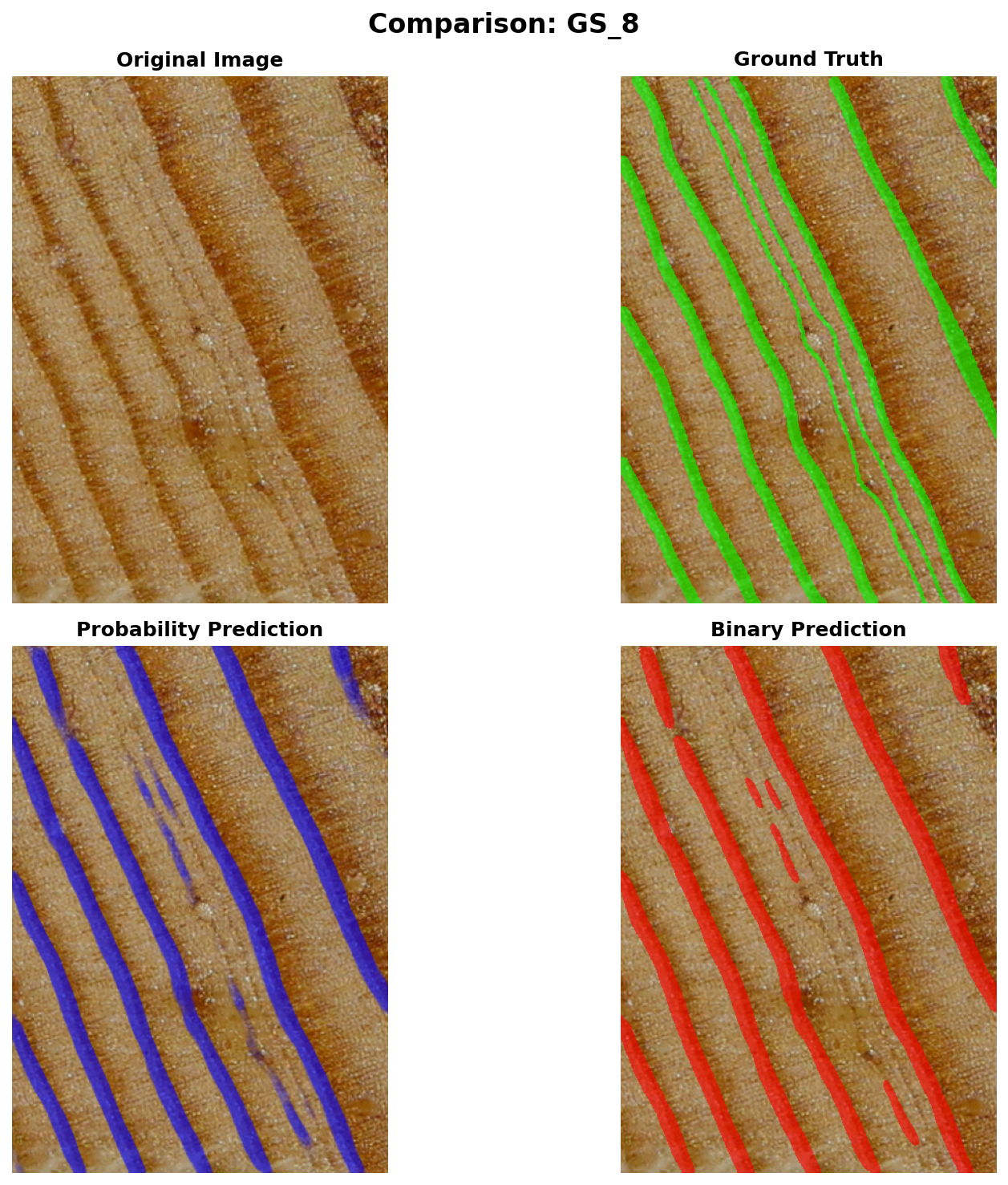

Supplement: S5 Appendix — The supplementary material contains two main folders: -plots: This folder includes, for each image of the GS studied in the focused evaluation dataset, the original image, the labels, the probability prediction (mean value of the 20 trained models), and the binary prediction (results obtained by applying a 0.5 threshold to the probability prediction). -results: This folder contains two files. results_table: A table including the distance measures (measured in µm/10) described in S2_Table. The column “tp_512px” corresponds to the true positives for the 512pxData. A value of 1 indicates a true positive, and a value of 0 means the ring was not correctly detected. histogram_results: A table containing the data used to generate Fig 4b. (ZIP) [file pone.0321841.s013.zip › plots/gs_plots_im_gt_predprob_predbin_512px/GS_8_composite.png]

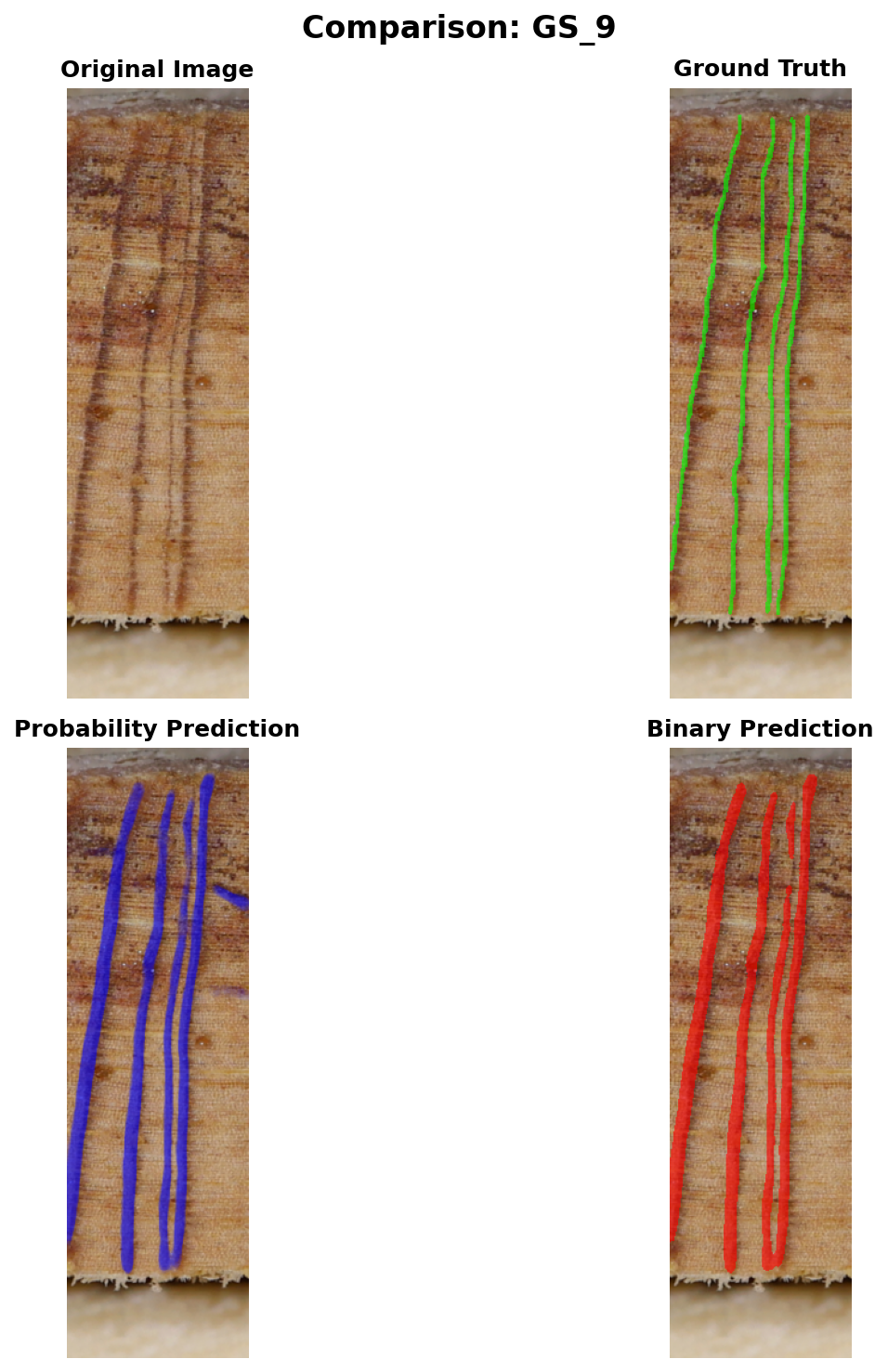

Supplement: S5 Appendix — The supplementary material contains two main folders: -plots: This folder includes, for each image of the GS studied in the focused evaluation dataset, the original image, the labels, the probability prediction (mean value of the 20 trained models), and the binary prediction (results obtained by applying a 0.5 threshold to the probability prediction). -results: This folder contains two files. results_table: A table including the distance measures (measured in µm/10) described in S2_Table. The column “tp_512px” corresponds to the true positives for the 512pxData. A value of 1 indicates a true positive, and a value of 0 means the ring was not correctly detected. histogram_results: A table containing the data used to generate Fig 4b. (ZIP) [file pone.0321841.s013.zip › plots/gs_plots_im_gt_predprob_predbin_512px/GS_9_composite.png]

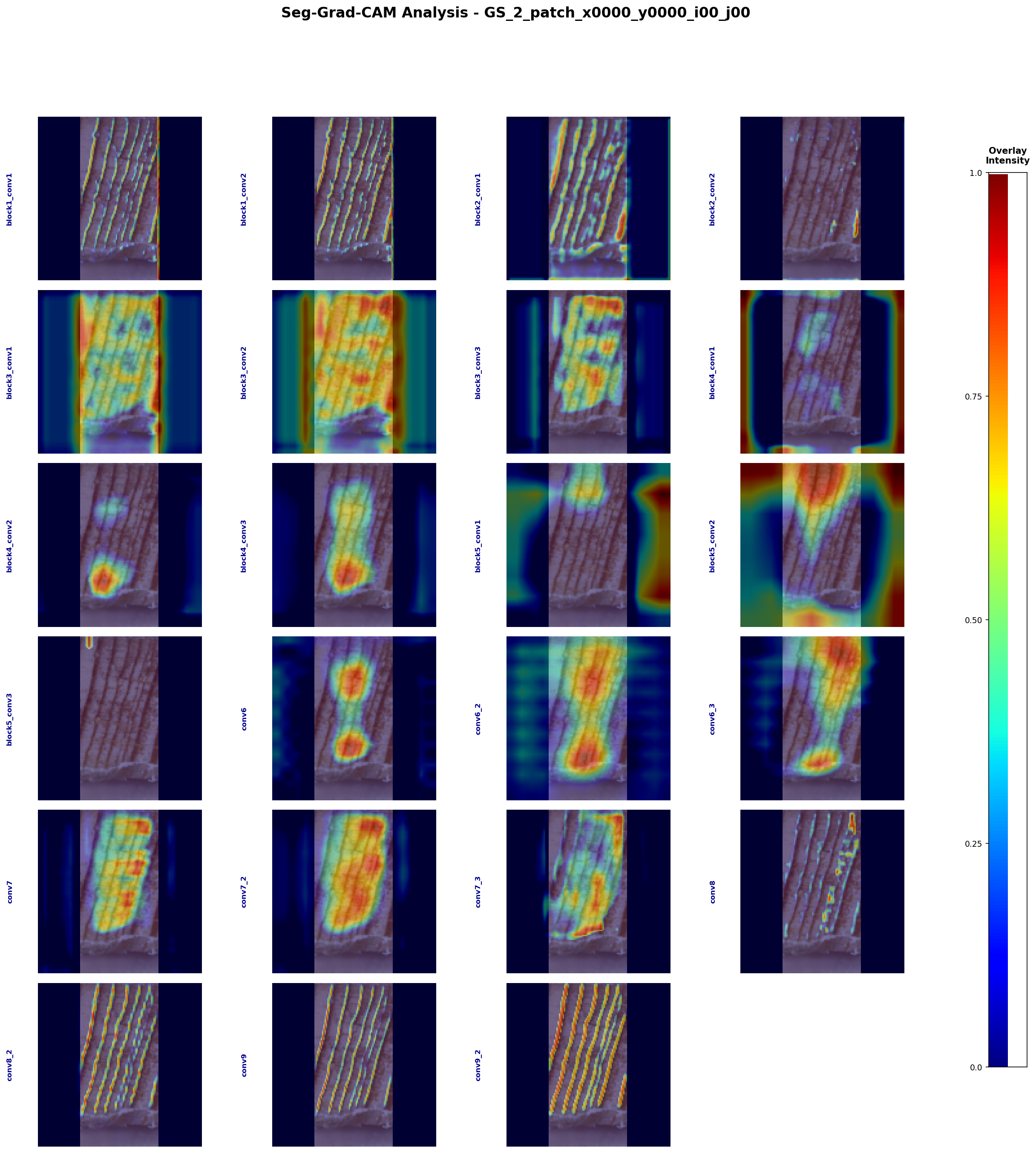

Supplement: S6 Appendix — (ZIP) [file pone.0321841.s014.zip › FTU-Net 128pxData.png]

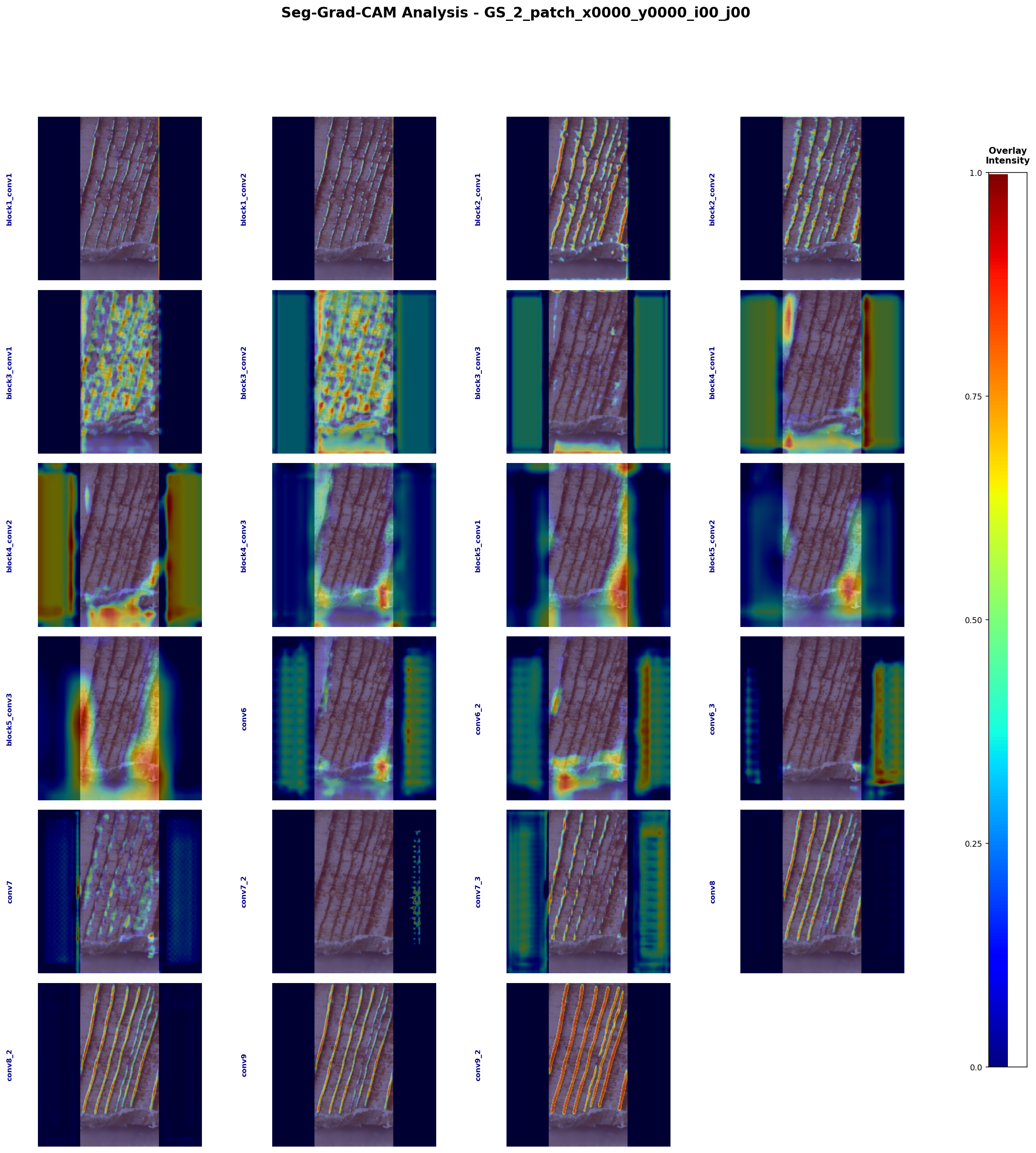

Supplement: S6 Appendix — (ZIP) [file pone.0321841.s014.zip › FTU-Net 246pxData.png]

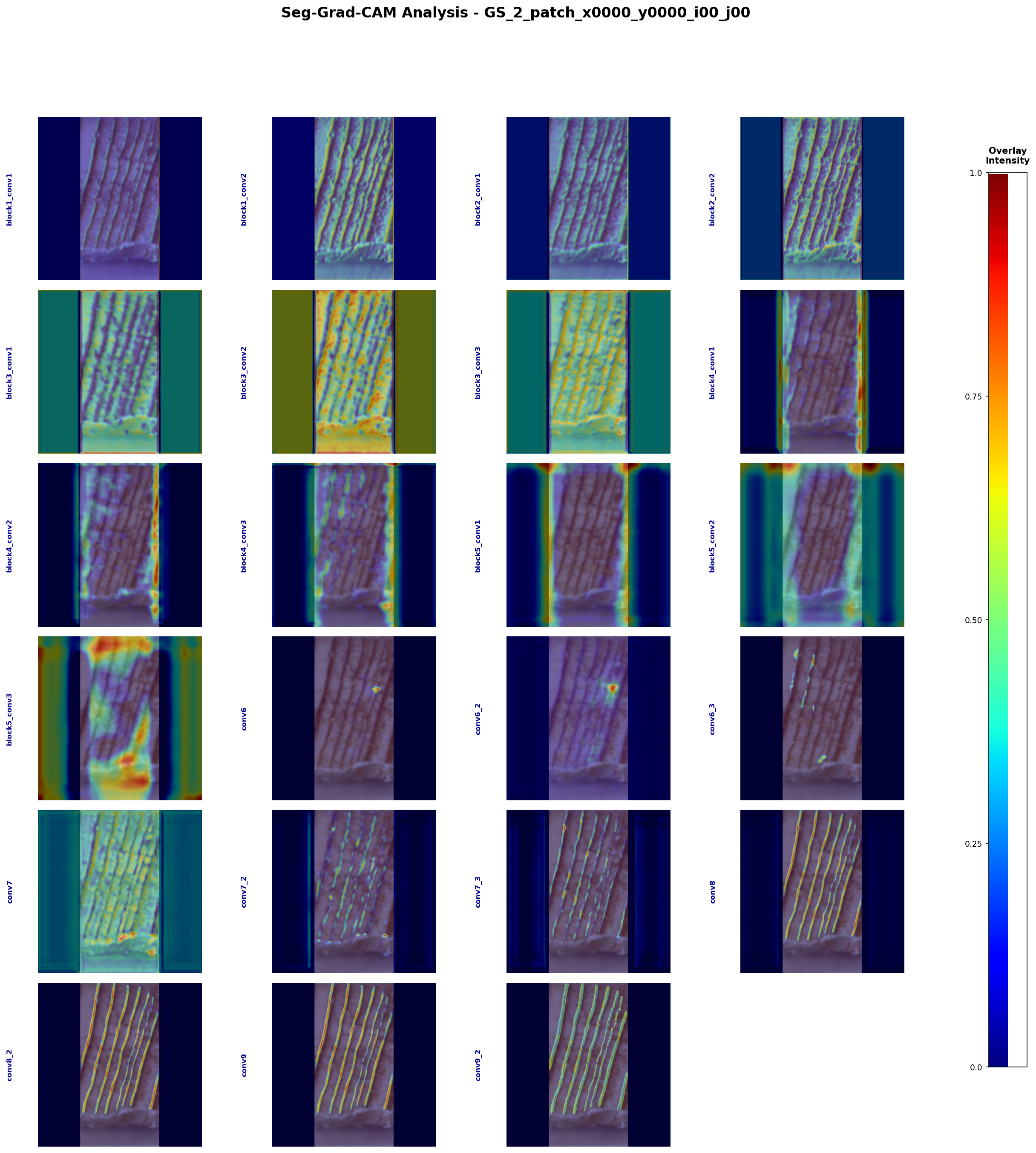

Supplement: S6 Appendix — (ZIP) [file pone.0321841.s014.zip › FTU-Net 512pxBData.png]

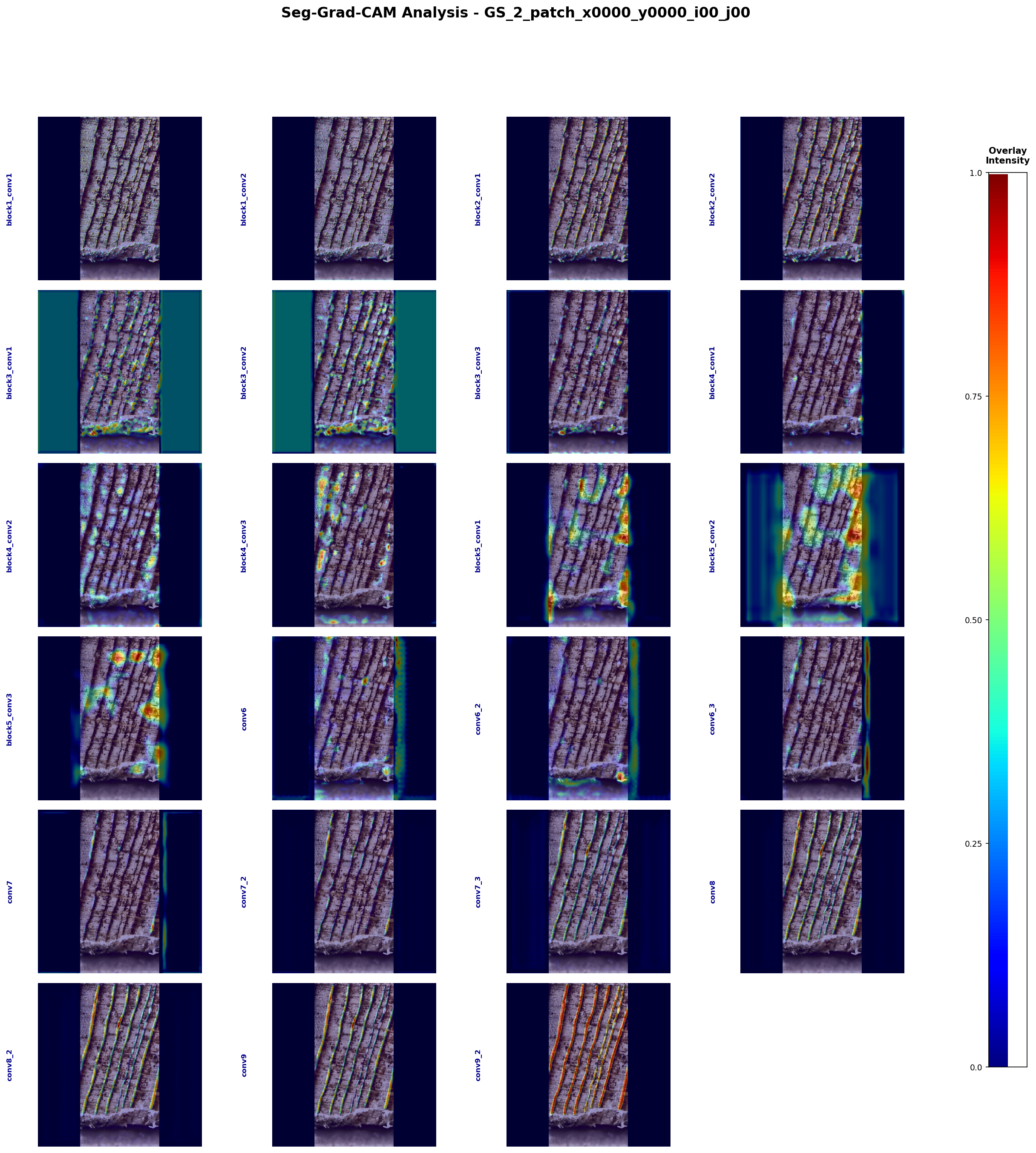

Supplement: S6 Appendix — (ZIP) [file pone.0321841.s014.zip › FTU-Net 512pxCLData.png]

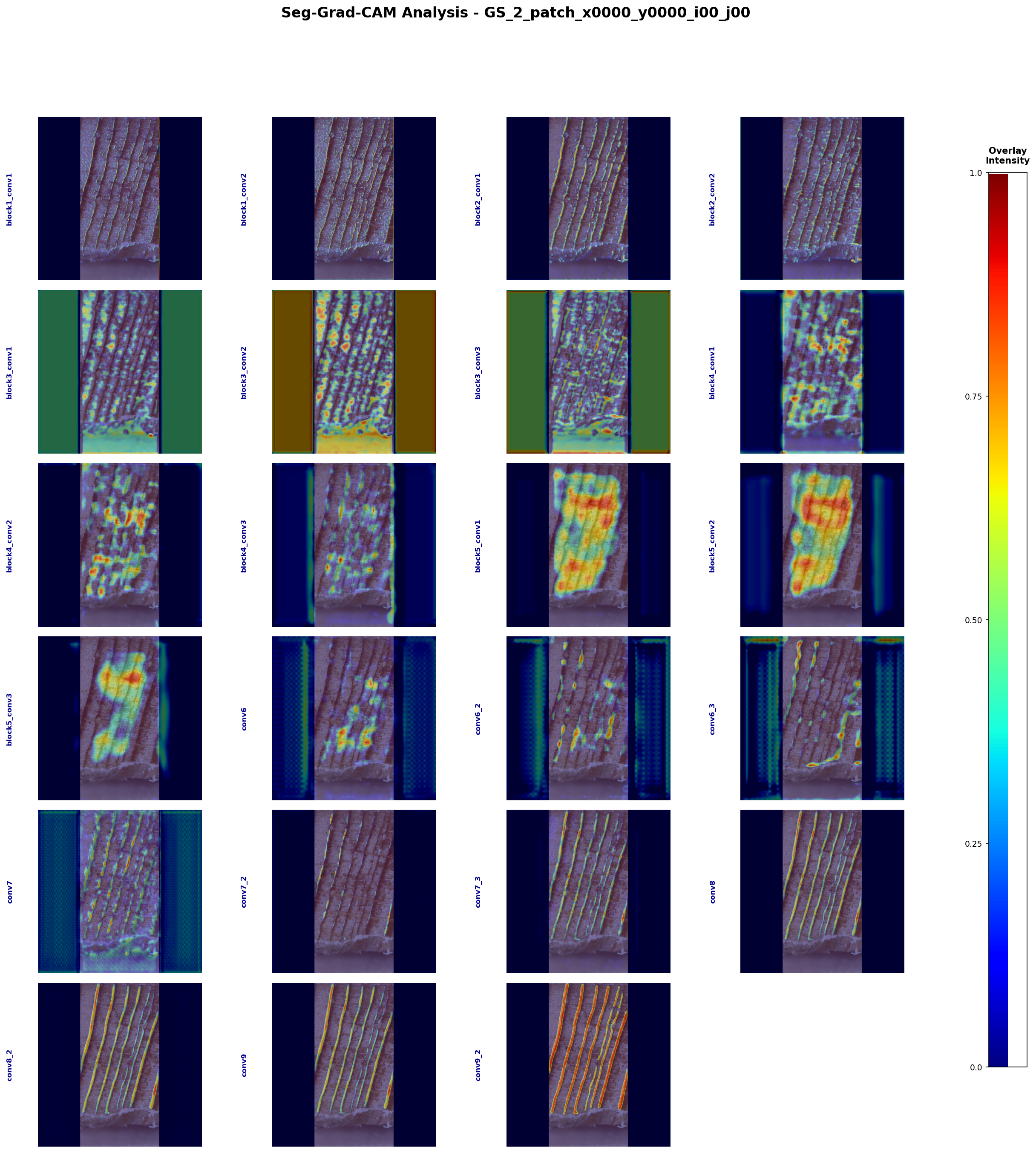

Supplement: S6 Appendix — (ZIP) [file pone.0321841.s014.zip › FTU-Net 512pxData.png]
