# Supplementary material for: Tree ring segmentation performance in highly disturbed trees using deep learning
Source: PLoS One. 2026 Jun 18;21(6):e0321841. doi: 10.1371/journal.pone.0321841 (PMC13278439; doi:10.1371/journal.pone.0321841)
Supplement: S3 Fig — In green, the preprocessing steps performed, and in purple, the partitioning strategies implemented. (PDF) [file pone.0321841.s003.pdf]

Tree ring segmentation performance in highly disturbed trees using deep learning

Joe David Zambrano-Suárez1,2, Jorge Pérez-Martín3, Alberto Muñoz-Torrero Manchado1, Juan Antonio Ballesteros Cánovas1**

1 Department of Geology, National Natural Science Museum, Spanish Research Council, Madrid, Spain
2 Research Institute of Water and Environmental Engineering, Universitat Politècnica de València, Spain
3 Department of Artificial Intelligence, National University of Distance Education, Madrid, Spain

**joe.zambrano@mncn.csic.es / juan.ballesteros@mncn.csic.es*

S3 FIGURE

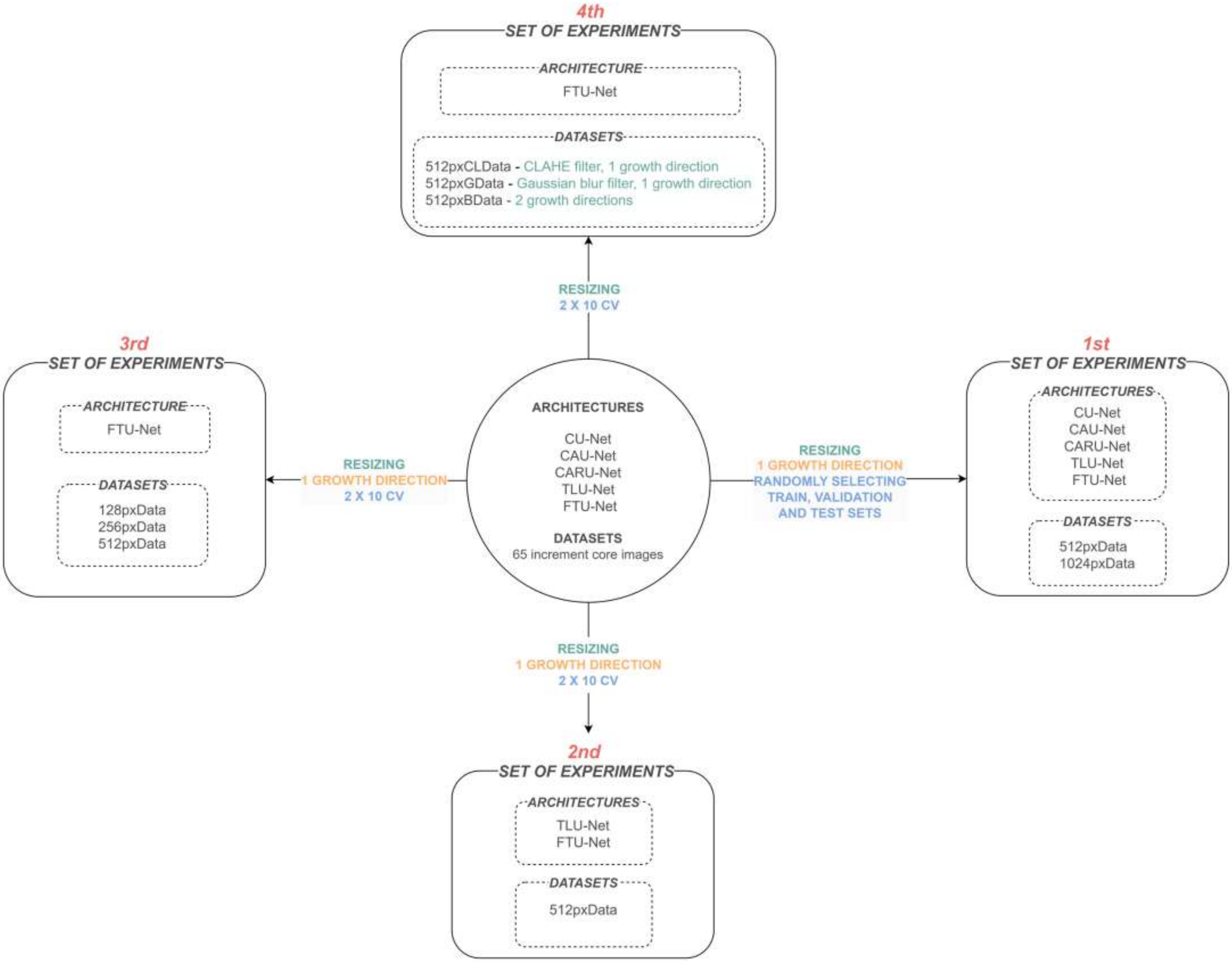

S3 Figure. Experimental design scheme. In green, the preprocessing steps performed, and in purple, the partitioning strategies implemented.
