# Supplementary material for: Tree ring segmentation performance in highly disturbed trees using deep learning
Source: PLoS One. 2026 Jun 18;21(6):e0321841. doi: 10.1371/journal.pone.0321841 (PMC13278439; doi:10.1371/journal.pone.0321841)

Tree ring segmentation performance in highly disturbed trees using deep learning

Joe David Zambrano-Suárez1,2, Jorge Pérez-Martín3, Alberto Muñoz-Torrero Manchado1, Juan Antonio Ballesteros Cánovas1**

1 Department of Geology, National Natural Science Museum, Spanish Research Council, Madrid, Spain
2 Research Institute of Water and Environmental Engineering, Universitat Politècnica de València, Spain
3 Department of Artificial Intelligence, National University of Distance Education, Madrid, Spain

**joe.zambrano@mncn.csic.es / juan.ballesteros@mncn.csic.es*

S1 Appendix. Graphic information about the fieldwork trip. (a) Zone map showing the sampled trees, including the 64 used for tree ring segmentation. (b) Sampled tree with a significant ramification near the soil. (c) Sampled tree with a scar on the trunk. (d) Sampled tree with trunk curvature.

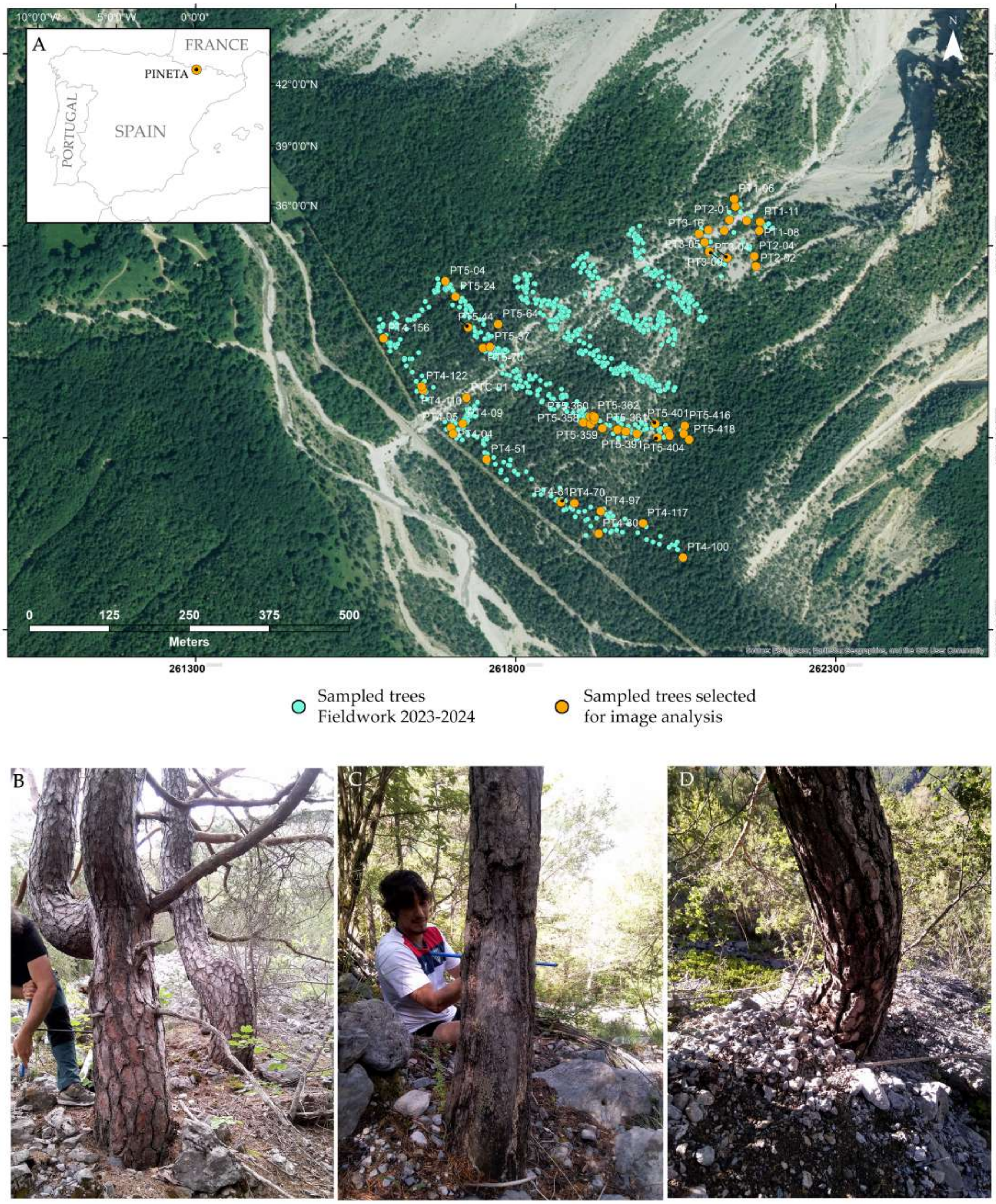

Supplement: S1 Appendix — (a) Zone map showing the sampled trees, including the 64 used for tree ring segmentation. (b) Sampled tree with a significant ramification near the soil. (c) Sampled tree with a scar on the trunk. (d) Sampled tree with trunk curvature. (PDF) [file pone.0321841.s009.pdf]
