# Supplementary material for: Tree ring segmentation performance in highly disturbed trees using deep learning
Source: PLoS One. 2026 Jun 18;21(6):e0321841. doi: 10.1371/journal.pone.0321841 (PMC13278439; doi:10.1371/journal.pone.0321841)
Supplement: S2 Appendix — (PDF) [file pone.0321841.s010.pdf]

Tree ring segmentation performance in highly disturbed trees using deep learning

Joe David Zambrano-Suárez1,2, Jorge Pérez-Martín3, Alberto Muñoz-Torrero Manchado1, Juan Antonio Ballesteros Cánovas1**

1 Department of Geology, National Natural Science Museum, Spanish Research Council, Madrid, Spain
2 Research Institute of Water and Environmental Engineering, Universitat Politècnica de València, Spain
3 Department of Artificial Intelligence, National University of Distance Education, Madrid, Spain

**joe.zambrano@mncn.csic.es / juan.ballesteros@mncn.csic.es*

S2 Appendix

Two parameter choices were made in this study for the application of Seg-Grad-CAM:

• Definition of the target concept: The target concept in Seg-Grad-CAM may correspond to a single pixel, a region of interest, or the entire image [1]. Because our objective was to understand why the model predicts certain pixels as belonging to tree rings, we selected the pixels predicted as tree rings as the target concept. This choice is consistent with common practice in the literature [2–4].

• Selection of the layer to which gradients are propagated: In the original Seg-Grad-CAM study [1], the authors proposed analyzing only the bottleneck layer, assuming it would be the most representative because it encodes the most abstract and complex features. However, subsequent work [4] demonstrated that examining multiple layers of the U-Net architecture provides deeper insight into how the model transforms its internal representations and how different regions of the input image contribute to the final segmentation. For this reason, Seg-Grad-CAM was applied across all layers of the model.

1. *Vinogradova K, Dibrov A, Myers G. Towards Interpretable Semantic Segmentation via Gradient-Weighted Class Activation Mapping (Student Abstract). Proc AAAI Conf Artif Intell. 3 de abril de 2020;34(10):13943-4.*

2. *Chung CT, Ying JJC. Seg-Eigen-CAM: Eigen-Value-Based Visual Explanations for Semantic Segmentation Models. Appl Sci. enero de 2025;15(13):7562.*

3. *Hasany SN, Petitjean C, Mériaudeau F. Seg-XRes-CAM: Explaining Spatially Local Regions in Image Segmentation. En: 2023 IEEE/CVF Conference on Computer Vision and Pattern Recognition Workshops (CVPRW) [Internet]. 2023 [citado 19 de noviembre de 2025]. p. 3733-8. Disponible en: https://ieeexplore.ieee.org/document/10208849*

4. *Hasany SN, Meriaudeau F, Petitjean C. The Do's and Don'ts of Grad-CAM in Image Segmentation as demonstrated on the Synapse multi-organ CT Dataset. En 2024 [citado 19 de noviembre de 2025]. Disponible en: https://openreview.net/forum?id=rnQUJLbODk*
